# Indole-3-Acetic Acid: Promising Protective Agent Against Methotrexate-Induced Liver Injury via Modulation of TLR4/NF-κB/Caspase-3 Pathway

**DOI:** 10.3390/ph18060828

**Published:** 2025-06-01

**Authors:** Sumayya A. Alturaif, Ahlam Alhusaini, Wedad Sarawi, Iman Hasan, Juman Alsaab, Rehab Ali, Raeesa Mohammed, Sahar S. Alotaibi, Faris Almutairi, Shaikha Alsaif, Ebtesam Alsultan, Ebtesam Aljasas, Sary Alsanea

**Affiliations:** 1 Department of Pharmacology and Toxicology, College of Pharmacy, King Saud University, P.O. Box 2457, Riyadh 11451, Saudi Arabia; 443204515@student.ksu.edu.sa (S.A.A.); wsarawi@ksu.edu.sa (W.S.); ihasan@ksu.edu.sa (I.H.); 443203447@student.ksu.edu.sa (J.A.); reali@ksu.edu.sa (R.A.); 445205548@student.ksu.edu.sa (S.S.A.); faralmutairi@ksu.edu.sa (F.A.); salsanea@ksu.edu.sa (S.A.); 2 Department of Anatomy, College of Medicine, King Saud University, P.O. Box 2925, Riyadh 11461, Saudi Arabia; rmohammad@ksu.edu.sa; 3 Department of Training Unit, College of Pharmacy, King Saud University, P.O. Box 2457, Riyadh 11451, Saudi Arabia; salsaif@ksu.edu.sa; 4 Department of Pharmacognosy, College of Pharmacy, King Saud University, P.O. Box 2457, Riyadh 11451, Saudi Arabia; ealsultan@ksu.edu.sa (E.A.); ealjassas@ksu.edu.sa (E.A.)

**Keywords:** methotrexate, indole 3-acetic acid, oxidative stress, inflammation, apoptosis

## Abstract

**Background and Purpose:** Methotrexate (MTX) is a widely used therapeutic agent for inflammatory and malignant diseases; however, its prolonged use is associated with hepatotoxicity through mechanisms that remain inadequately understood. This study aims to elucidate these mechanisms and assess the hepatoprotective potential of indole-3-acetic acid (IAA). **Methods:** Rats were allocated into five groups: control (group 1), IAA-treated (group 2), MTX-treated (group 3), quercetin (QUR) + MTX (group 4), and IAA + MTX (group 5). Hepatic function was assessed through the evaluation of serum liver enzymes, oxidative stress, and inflammatory and apoptotic markers using biochemical, molecular, histopathological, and immunohistochemical analyses. **Results:** The MTX-treated group demonstrated a significant increase in hepatic oxidative stress, inflammation, and apoptotic markers. Co-administration of IAA or QUR with MTX markedly reduced malondialdehyde (MDA) levels, while enhancing glutathione (GSH) levels and superoxide dismutase (SOD) activity. Moreover, hepatic inflammatory markers, including TNF-α, IL-6, and IL-1β, were significantly decreased in the IAA- and QUR-treated groups. Immunohistochemical analysis further revealed a reduced expression of NF-κB, TLR4, and caspase-3 in hepatic tissues following QUR-MTX or IAA-MTX treatments. **Conclusions:** IAA exhibited hepatoprotective effects against MTX-induced liver injury, comparable to QUR, by modulating the TLR4/NF-κB/caspase-3 pathway. These findings highlight its potential clinical application in reducing MTX-associated hepatic complications.

## 1. Introduction

Methotrexate (MTX) is an immunosuppressant and chemotherapeutic agent that is widely used for the treatment of multiple disorders such as rheumatoid arthritis, psoriatic arthritis, and cancer [1]. MTX exerts its effects by inhibiting the enzyme dihydrofolate reductase (DHFR); this enzyme catalyzes the conversion of dihydrofolate into tetrahydrofolate, the active form of folic acid, which is necessary for the synthesis of the nucleotides of both DNA and RNA [2]. Nowadays, MTX is considered an essential medication for patients suffering from rheumatic arthritis [3]. Despite its usefulness, preclinical and clinical studies have reported that the long-term use of MTX is associated with multiorgan toxicities, including hepatic injury, nephrotoxicity, acute lung injury, and the dysregulation of gut microbiota [4,5]. The exact mechanism of the liver toxicity caused by MTX remains unclear. Nevertheless, certain studies suggest that MTX metabolites may lead to oxidative stress, resulting in damage to liver cells, which can subsequently trigger inflammation and apoptosis [6]. The MTX metabolite is implicated in the onset of intracellular oxidative stress, which causes an overproduction of ROS, causing a depletion in mitochondrial enzymatic and non-enzymatic antioxidant machinery [7]. Inflammation can be associated with increased ROS production as a body defense mechanism [8].

Furthermore, studies have shown that the generation of ROS by MTX metabolite causes an upregulation of pro-inflammatory markers, such as nuclear factor-kappa B (NF-κB) expression, which triggers inflammatory responses in the affected cells [7,9]. NF-ĸB is a transcription factor that regulates many genes involved in inflammatory responses to toxins. It promotes the production of pro-inflammatory cytokines from macrophages such as interleukins (IL-1 and IL-6) and tumor necrosis factor-α (TNF-α) [10,11]. It has been shown that the activation of NF-κB signaling cascades is initiated by toll-like receptor 4 (TLR4), which detects any invaders and activates innate and adaptive immune responses, such as the secretion of inflammatory mediators [11].

Recently, an increasing body of research has focused on plant-based antioxidants, highlighting their natural origin, reduced side effects, and enhanced effectiveness [12]. Indole-3-acetic acid (IAA) is the main hormone of auxin, which possesses antioxidant and anti-inflammatory properties [13]. Although IAA has been extensively studied in plants, its effects in vivo have not been thoroughly investigated. Additionally, the hepatoprotective potential of IAA against MTX-induced liver injury in rats remains unexplored. Therefore, it is essential to find an effective strategy to protect the liver during MTX therapy. This study examined the role of oxidative stress, inflammation, and apoptosis, with a focus on the involvement of the TLR4/NF-κB/caspase-3 signaling pathway, in the modulatory mechanisms by which IAA protects against MTX-induced liver injury in a rat model.

## 2. Results

### 2.1. IAA Restored the Levels of Serum ALT and AST

**Figure 1:**
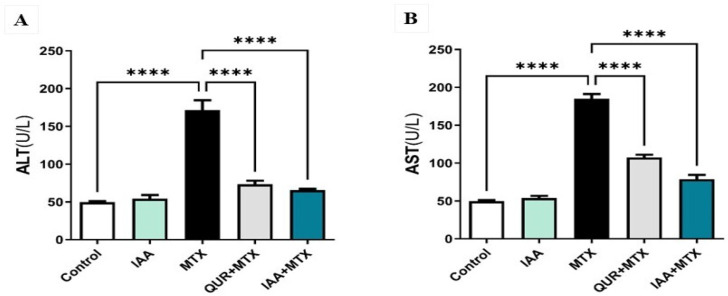
Effects of QUR and IAA on serum activity of (**A**) ALT and (**B**) AST in MTX-induced liver injury in rats. Data are expressed as mean ± SEM (n = 6). **** *p* < 0.0001.

As shown in Figure 1A, serum ALT activity significantly increased in the MTX-treated group compared to the control group (*p* < 0.0001). The co-treatment of either QUR or IAA with MTX significantly reduced the ALT activity level compared to the MTX-treated group (*p* < 0.0001). Similarly, as demonstrated in Figure 1B, serum AST activity was significantly increased in the MTX-treated group compared to the control group (*p* < 0.0001). Co-treatment with either QUR or IAA with MTX significantly reduced AST activity compared to the group treated with MTX (*p* < 0.0001).

### 2.2. IAA Restored the Hepatic Levels of MDA, GSH, and SOD

**Figure 2:**
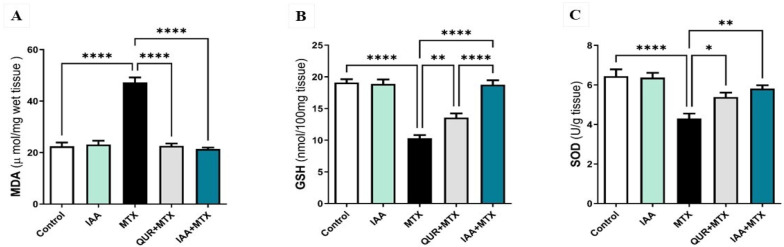
Effects of QUR and IAA on the levels of (**A**) MDA, (**B**) GSH, and (**C**) SOD in MTX- induced liver injury in rats. Data are expressed as mean ± SEM (n = 6). * *p* < 0.05, ** *p* < 0.01, **** *p* < 0.0001.

As illustrated in Figure 2A, MDA levels were significantly increased in the MTX group compared to the control group (*p* < 0.0001). Treatment with either QUR or IAA significantly decreased the levels of MDA compared to the MTX group (*p* < 0.0001). In addition, as shown in Figure 2B, GSH levels were significantly decreased in the MTX group compared to the control group (*p* < 0.0001). Rats treated with either QUR or IAA combined with MTX showed a significant elevation in GSH levels when compared to the MTX-treated group of (*p* < 0.01) or (*p* < 0.0001), respectively. Interestingly, IAA produced a significant increase in GSH levels compared to QUR treatment; the IAA + MTX group showed a significant restoration in GSH levels compared with QUR (*p* < 0.0001). Figure 2C demonstrates that the activity levels of SOD were markedly diminished in the MTX-treated group when compared to the control group (*p* < 0.0001). The rats treated with QUR or IAA exhibited a significant increase in SOD activity levels when compared to the MTX group (*p* < 0.05 and *p* < 0.01).

### 2.3. IAA Downregulated the Expression Levels of Hepatic TNF-α, IL-1β, and IL-6

**Figure 3:**
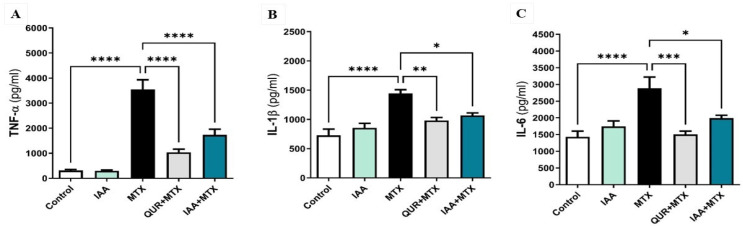
Effects of QUR and IAA on the expression levels of (**A**) TNF-α, (**B**) IL-β, and (**C**) IL-6 level in MTX-induced hepatotoxicity in rats. Data are expressed as mean ± SEM (n = 6). * *p* < 0.05, ** *p* < 0.01, *** *p* < 0.001, **** *p* < 0.0001.

As shown in Figure 3A, the administration of a high dose of MTX caused a significant upregulation of TNF-α compared to the control group (*p* < 0.0001). Treatment with either QUR or IAA markedly downregulated the expression of TNF-α in comparison to MTX-only. Figure 3B illustrates that the expression levels of hepatic IL-1β were significantly upregulated in the MTX-treated group compared to the control group (*p* < 0.0001). When the MTX rats were treated with either QUR or IAA, the hepatic levels of IL-1β markedly declined in comparison to the MTX group by (*p* < 0.01) or (*p* < 0.05), respectively. Additionally, as shown in Figure 3C, the level of IL-6 was markedly elevated in the MTX group compared to the control group (*p* < 0.0001). When the MTX-treated rats were pretreated with either QUR or IAA, the expression level of IL-6 was significantly decreased by (*p* < 0.001) or (*p* < 0.05), respectively, in comparison to MTX only.

### 2.4. IAA Improved the Morphological Changes in Liver Sections Induced by MTX

**Figure 4:**
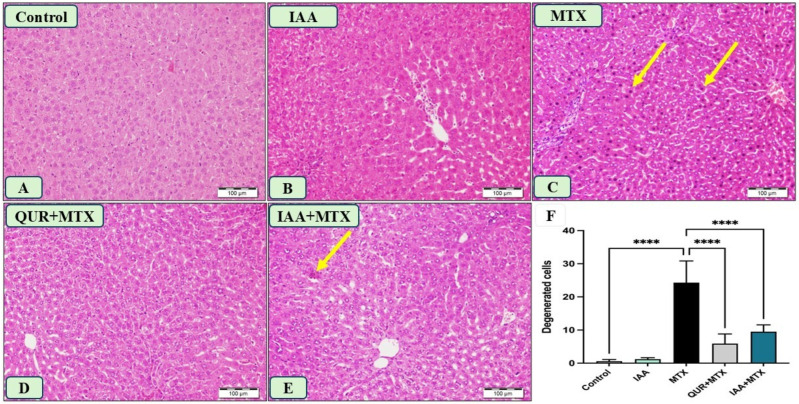
Photomicrograph of liver sections stained with H&E (n = 6), scale bar 100 µm. (**A**) The control liver section exhibits normal hepatic lobules with normal hepatocytes and sinusoids. (**B**) The liver section from the rat that received IAA also shows a normal architecture. (**C**) The liver section from rats that received MTX shows many hepatocytes with pyknotic nuclei and degenerated cytoplasm (arrows). (**D**) The liver section of rats that received QUR + MTX displays normal hepatic features. (**E**) The liver section from rats that received IAA + MTX shows a marked decrease in hepatocyte degeneration (arrow). (**F**) A quantitative analysis revealed a significant decrease in the degenerated hepatocytes in the groups treated with either QUR or IAA compared to MTX-intoxicated group. **** *p* < 0.0001.

The liver sections obtained from the control and IAA-treated rats showed normal hepatocytes, as seen in Figure 4A,B. Panel C represents a liver section from the rats that received MTX, displaying many hepatocytes with pyknotic nuclei and a degenerated cytoplasm. In panel D, the liver section of a rat that received QUR + MTX exhibits normal hepatic features. Moreover, panel E represents a liver section from a rat that received IAA + MTX, which shows a marked decrease in hepatocyte degeneration compared to panel C. Figure 4F shows a significant reduction in the degenerated hepatocytes of the groups treated with either QUR or IAA compared to the MTX group.

### 2.5. IAA Reduced the Positive Immunoreactivity of TLR4, NF-κB, and Caspase-3 in the Hepatocytes Induced by MTX

**Figure 5:**
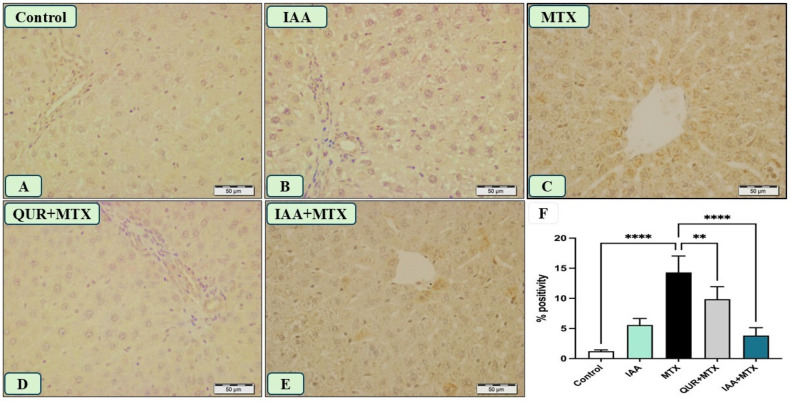
Photomicrograph of TLR4 immunostained liver sections (n = 6), scale bar 50 µm. (**A**,**B**) Control and IAA show the absence of immune positivity. (**C**) Liver sections from rats that received MTX show patches of hepatocytes with the intense immune-stained cytoplasm of hepatocytes. (**D**) The liver sections from the rats that received QUR + MTX show an absence of immune reaction. (**E**) The liver sections from rats who received IAA + MTX show a marked decrease in the number and intensity of immune reactive hepatocyte cytoplasm. (**F**) A quantitative analysis revealed a significant decrease in the TLR4 expression in the groups treated with either QUR or IAA compared to the MTX-intoxicated group. ** *p* < 0.01, **** *p* < 0.0001.

Figure 5A,B displays the negative immunoreactivity of TLR4 in the hepatocytes of the rats that received the vehicle and IAA, respectively. The administration of MTX induced a strong immune reaction for TLR4 compared to the control, as shown in Figure 5C. Treatment with either QUR or IAA combined with MTX markedly reduced the expression of TLR4, as shown in panels D and E. As shown in Figure 5F, the protein expression of TLR4 in liver tissue was significantly reduced in rats that received either QUR or IAA compared to MTX-intoxicated rats.

**Figure 6:**
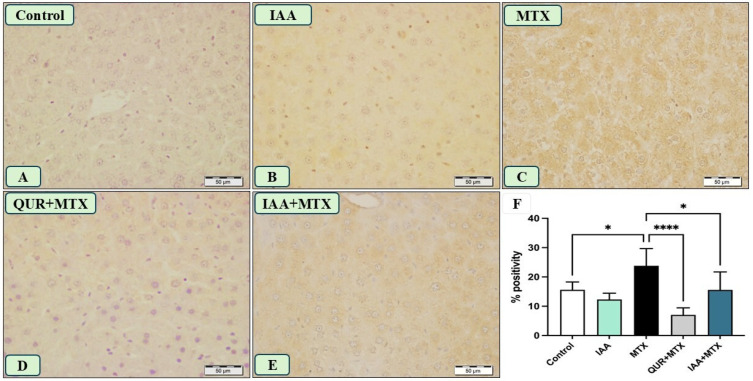
Photomicrograph of NF-κB immunostained liver sections (n = 6), scale bar 50 µm. (**A**,**B**) Control and IAA show the absence of immune positivity. (**C**) Liver sections from rats receiving MTX show large patches of hepatocytes with the intense immune-stained cytoplasm of hepatocytes. (**D**) The liver sections from the rats that received QUR + MTX show an absence of immune reaction. (**E**) The liver sections from rats who received IAA + MTX show a marked decrease in the number and intensity of immune reactive hepatocyte cytoplasm. (**F**) Aquantitative analysis revealed a marked reduction in the NF-κB expression in the groups treated with either QUR or IAA compared to the MTX-intoxicated group. * *p* < 0.05, **** *p* < 0.0001.

Likewise, administering a high dose of MTX caused a strong immune reaction for hepatic NF-κB, as shown in Figure 6C, compared with the reactions observed in panels A and B, which represent liver sections obtained from the control and IAA groups, respectively. Panel D represents a liver section obtained from the QUR + MTX group, which shows an absence of an immune reaction. Lastly, panel E displays a liver section from a rat that received IAA + MTX, and it shows a marked decrease in the intensity of immune reactivity compared to the MTX-treated group (panel C). Figure 6F shows a marked reduction in the expression of hepatic NF-κB in rats treated with either QUR or IAA compared to MTX-intoxicated rats.

**Figure 7:**
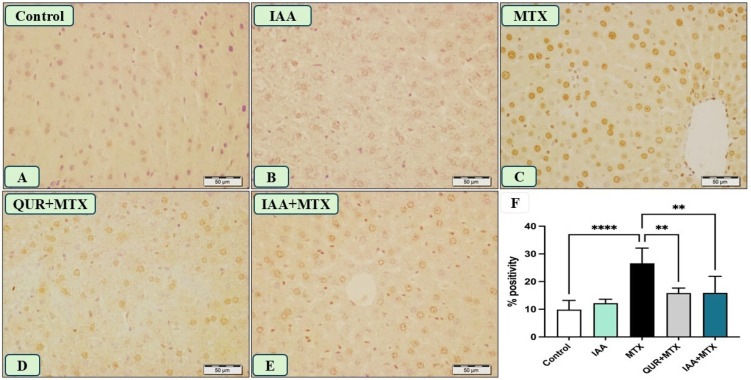
Photomicrograph of caspase-3 immunostained liver sections (n = 6), scale bar 50 µm. (**A**,**B**) Control and IAA show the absence of immune positivity. (**C**) The liver sections from the rats that received MTX show many hepatocytes nuclei with strong immune reactions. (**D**) The liver sections from the rats that received QUR + MTX show a decreased number of positive immune-stained nuclei with a low density. (**E**) The liver section from rats that received IAA + MTX shows a marked reduction in the number of immune reactive nuclei that appear faintly stained. (**F**) A quantitative analysis revealed a marked decline in the hepatic caspase-3 expression in the groups treated with either QUR or IAA compared to the MTX-intoxicated group. ** *p* < 0.01, **** *p* < 0.0001.

As presented in Figure 7, panel C shows many hepatocytes’ nuclei, displaying strong positive immune reactions to caspase-3 in a liver section obtained from a rat receiving MTX; this is in comparison to the reactions observed in panels A and B, which represent liver sections from the control and IAA groups, respectively. Panels D and E display liver sections from the rats that received MTX and were treated with QUR and IAA, respectively, showing a reduction in positive immunoreactivity to caspase-3 in comparison to that shown in panel C. The quantification analysis of the expression of caspase-3 in the liver tissues of all the groups revealed a significant decline in its expression in the QUR- and IAA- treated groups compared to the MTX group (Figure 7F).

## 3. Discussion

Hepatotoxicity is a significant global issue in healthcare foundations, and it is one of the major complications following MTX treatment [14]. MTX is well tolerated; however, it has the potential to induce liver toxicity [15]. This adverse effect limits the clinical use of MTX [16]. The exact molecular mechanism of MTX-induced hepatotoxicity is not well defined; however, several studies have implied the possibility of oxidative stress, which triggers the release of inflammatory cytokines [14]. Recently, natural products have gained significant attention among researchers as a protective strategy against the toxicity induced by the long-term use of therapeutic agents. They are preferable due to their natural origin and fewer side effects [12]. IAA is a plant-based antioxidant that regulates various aspects of plant growth and development [13]. QUR, a plant flavonoid, is a well-known and powerful antioxidant with ROS-scavenging activity [17]. Additionally, QUR has well-documented anti-inflammatory and antiapoptotic properties, and it has shown protective effects against various toxicities, particularly MTX-induced liver damage [18,19]. While indole-3-carbinol (I3C) also exhibits pharmacological benefits, including senolytic activity and protection against drug-induced toxicity [20], QUR was selected in this study as the reference compound due to its widely accepted use as a potent antioxidant in hepatotoxicity models [21]. Thus, the protective effectiveness of QUR is both relevant and worthy of remark in this experimental model. There are a few published studies that have investigated the effects of IAA on animal models, but to the best of our knowledge, no studies have investigated the protective effects of IAA against MTX-induced liver injury. Hence, the current study aims to investigate the molecular mechanisms involved in liver injury induced by MTX and the protective action of IAA, focusing on oxidative stress, inflammation, and apoptosis mechanisms.

The current study used a single intraperitoneal MTX dose of 20 mg/kg to induce hepatic injury. The significant increase in the serum levels of ALT and AST proved the toxicity of MTX. These results are in agreement with those of several studies investigating the hepatic toxicity induced by MTX in rats [14,22,23,24,25]. AST and ALT enzymes are considered sensitive and initial predictors of liver function [26]. As a cytotoxic drug, MTX enters the cell and is then converted to MTX-PG metabolite, which inhibits the DHFR enzyme; this leads to the suppression of the synthesis of DNA and repair mechanisms [14]. This toxic mechanism leads to the destruction of hepatocyte membranes and organelles, which further causes the leakage of liver enzymes [2,14]. The current study showed that treatment with either QUR or IAA significantly decreased the serum levels of ALT and AST compared to those of the MTX group. In agreement with our results, a recent study revealed a reduction in AST and ALT activity levels after using IAA to combat high-fat diet (HFD)-induced nonalcoholic fatty liver disease (NAFLD) in C57BL/6 mice [27].

In the current work, we have shown that oxidative stress was implicated in MTX-induced liver injury. The MTX-treated group exhibited increased MDA levels and decreased GSH levels and SOD activity in liver tissues compared to the control group. A recent study reported an elevation in MDA levels in rat liver and kidney tissues and a remarkable decrease in SOD activity levels [24]. Another study revealed a reduction in NADPH and GSH with the ROS elevation in MTX-treated groups, resulting in hepatotoxic manifestations such as oxidative damage [6]. A significant depletion in GSH levels following MTX treatment was evident in two in vitro studies [28,29]. It was assumed that MTX-PG is conserved in cells for a long time, causing a depletion in mitochondrial enzymatic and non-enzymatic antioxidant machinery [7]. It then triggers several intracellular pathological events, resulting in the generation of ROS [30]. The excessive ROS generation caused by MTX metabolites leads to increased lipid peroxidation, which contributes to further hepatic cell membrane damage [7,14].

Treatment with either IAA or QUR modulated the oxidative stress induced by MTX; this was confirmed by decreasing MDA levels and a restoration to normal levels of antioxidant scavengers, including GSH and SOD. Interestingly, treatment with IAA showed a superior antioxidant effect by increasing GSH levels compared to QUR. To the best of our knowledge, this is the first study exploring IAA’s antioxidant effectiveness against MTX-induced hepatotoxicity in a rat model. A recent study confirmed the antioxidant activity of IAA in macrophage cell lines [31]. Another study revealed the antioxidant effects of IAA through a reduction in MDA levels and a notable elevation in GSH levels and SOD activity in a nonalcoholic fatty liver disease (NAFLD) mouse model [27]. The antioxidant properties of IAA could be due to it being one of the tryptophan (Trp) metabolites. Trp is an essential amino acid that interacts with gut microflora to produce its metabolites [31]. Trp and its metabolites, including IAA, could attenuate oxidative stress through their free radical-scavenging activity [32,33]. QUR is another powerful antioxidant used in our study, which exerts an antioxidant effect by maintaining oxidative balance. It has been reported in a previous study that QUR has a protective effect by enhancing the expression levels of defense antioxidants such as SOD and GSH [34].

Another mechanism contributing to the hepatoprotective effects of IAA and QUR is the reduction in excessive inflammatory mediator production. Therefore, this study examined the anti-inflammatory effects of IAA and QUR in the context of MTX-induced liver injury. While MTX is recognized for its anti-inflammatory properties in the treatment of rheumatoid arthritis, its impact on liver health is also of interest [3]. MTX is known to inhibit the deamination of adenosine, leading to its accumulation, which diminishes inflammation [7,29]. However, both an overdose of MTX and chronic treatment can lead to the accumulation of its metabolites in hepatocytes, causing oxidative stress, which is highly implicated in the release of pro-inflammatory cytokines that worsen the injury [7,23]. In the present study, the MTX group showed an upregulation of pro-inflammatory cytokines, including TNF-α, IL-1β, and IL-6. Our results agree with those of previous studies, which revealed the upregulation of the hepatic levels of TNF-α, IL-1β, and IL-6 after treatment with MTX [9,14,27]. The administration of either IAA or QUR was able to attenuate the inflammatory response induced by MTX’s administration via reducing the levels of TNF-α, IL-1β, and IL-6. It has been revealed that IAA can attenuate inflammatory responses in vitro, as evidenced in hepatocytes and macrophages, by reducing the expression levels of TNF-α and IL-1β [35]. Moreover, a previous study on macrophage cell lines provided similar evidence of the anti-inflammatory action of IAA by highlighting the attenuated expressions of IL-1β and IL-6 [31]. A recent in vivo study revealed the anti-inflammatory effects of IAA in valproate (VPA)-induced liver toxicity in rats through the downregulation of TNF-α and IL-1β [36].

To further study the inflammatory mechanisms induced by the administration of a high dose of MTX, we investigated the expressions of TLR4 and NF-κB using immunohistochemistry. We found that the immunoreactions of TLR4 and NF-κB in liver tissues were strongly positive following MTX treatment, and their immunoreactivity was reduced after the administration of either QUR or IAA. The activation of TLR4 causes the phosphorylation of IκB and translocation of NF-κB to the nucleus. This pathway leads to the activation of NF-κB and the further release of pro-inflammatory cytokines [11,37]. In addition, it has been reported that NF-κB is upregulated in liver and kidney tissue after MTX treatment [9]. As suggested by Ezhilarasan, MTX metabolites cause the activation of NF-κB expression, which triggers an inflammatory response by releasing inflammatory cytokines during excessive ROS generation [7]. In agreement with our results, a recent study revealed the protective effects of IAA through a decrease in the expression of TLR4 in a VPA-induced liver injury rat model [36].

The involvement of the apoptosis mechanism in the liver injury induced by MTX is another signaling mechanism studied in the current work. The immunohistochemical analysis of the expression of caspase-3 in the liver section obtained from the group that received MTX showed many hepatocyte nuclei with strong immunoreactive signals. Arab et al. proved that MTX upregulates the expression of the proapoptotic factor and downregulates the expression of the antiapoptotic factor [38]. In addition, more evidence has revealed the proapoptotic effects of MTX by demonstrating an increase in the expression of caspase-3 in hepatorenal toxicity [39]. Furthermore, a recent study has agreed with this evidence on the activation of MTX in the caspase-3 cascade in hepatorenal toxicity [9]. As MTX metabolites generate ROS, this leads to the activation of the intrinsic pathway of apoptosis and eventually causes cell death [7]. Treatment with either IAA or QUR caused the downregulation of caspase-3 expression, which was seen as a marked reduction in the numbers of positive immunoreactive nuclei in the hepatocytes. Our results confirmed the antiapoptotic effects of IAA via a decrease in the expression of caspase-3, which attenuated the proapoptotic effects of MTX. Papi et al. reported the antiapoptotic effects of IAA via the inhibition of the caspase cascade in a renal ischemic injury rat model [40].

The histopathological changes in the liver tissues of the MTX-treated group were affirmably conclusive, as the tissues exhibited many hepatocytes with pyknotic nuclei and a degenerated cytoplasm. These findings were confirmed in several studies after a single intraperitoneal dose of MTX (20 mg/kg) in rats [14,24]. Treatment with either IAA or QUR markedly reduced the number of degenerative hepatocytes. Parallel to our results, Aljarboa and colleagues (2023) have revealed the pronounced protection of IAA against VPA-induced liver injury via improvements in the liver tissue architecture [36].

Overall, MTX-induced liver toxicity is linked with serial deleterious events, including oxidative stress, inflammation, and apoptosis. The excessive production of MTX-PG metabolites can deplete mitochondrial antioxidants and induce lipid peroxidation, leading to oxidative stress. The generation of ROS can mediate the release of inflammatory cytokines and activate pro-inflammatory signaling pathways. These events collectively trigger the intrinsic pathway of apoptosis and ultimately result in hepatic cell death. On the other hand, the protective effect of IAA is mainly due to its antioxidant activity, which may be attributed to it being one of the Trp metabolites. By scavenging free radicals, IAA effectively attenuates oxidative stress, thereby reducing inflammatory and apoptotic responses.

## 4. Materials and Methods

### 4.1. Drugs and Chemicals

MTX isotonic liquid vials were obtained from King Saud Hospital in Unaizah. IAA raw powder (ab146402) was purchased from Abcam^®^ (Waltham, MA, USA) and freshly prepared in 1% carboxymethylcellulose (CMC) solution. QUR (used as a reference drug) was purchased from a local pharmacy. Alanine aminotransferase (ALT) and aspartate aminotransferase (AST) were purchased from Linear (Barcelona, Spain). TNF-α (SEKR-0009), IL-1β (SEKR-0002), and IL-6 (SEKR-0005) ELISA kits were purchased from Solarbio Life Sciences, Beijing, China. Primary polyclonal TLR4 rabbit (bs-20594R), NF-κB rabbit (ab16502), and caspase-3 rabbit (ab4051) antibodies were purchased from Bioss Antibodies Inc.^®^ (Woburn, MA, USA). Biotinylated anti-rabbit IgG (H + L) (BA-1000) secondary antibody was purchased from Vector Laboratories, Inc. (Burlingame, CA, USA).

### 4.2. Experimental Animals and Treatment

Adult male Wistar albino rats (200–250 g) were obtained from the Animal Care Center at the College of Pharmacy, King Saud University, Riyadh, Saudi Arabia. The animals were housed in standard polypropylene cages (3 rats/cage) with free access to food and water. The rats were maintained in a controlled environment at 25 °C under a 12 h dark/light cycle and acclimated for one week before the experiments. All the experiments were carried out in accordance with the recommendations of the Research Ethics Committee and the Institutional Animal Care and Use Committee (IACUC), and the study protocol was approved by the Research Ethics Committee, King Saud University (Ethics Approval No. KSU-SE-23-08).

**Figure 8:**
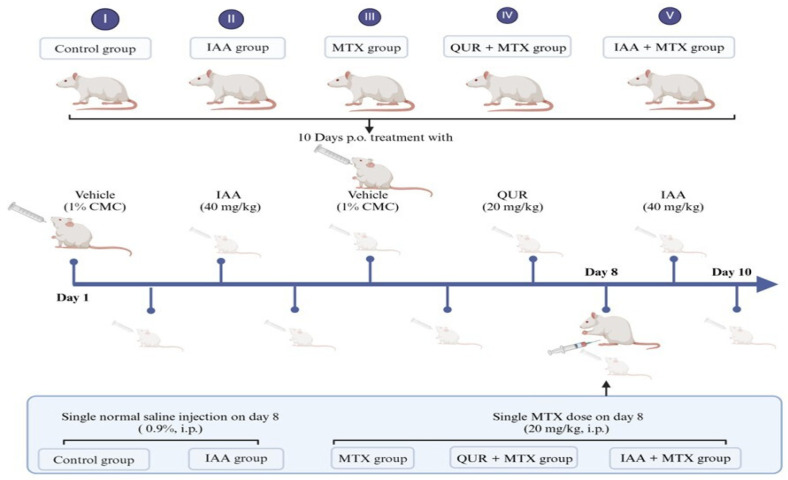
The experimental design and animal grouping. (I) Control group: Rats were given an equivalent volume of 1% CMC orally and a single i.p. dose of normal saline (0.9%) on day 8. (II) IAA group: Rats were given IAA (40 mg/kg, p.o.) for 10 days and a single i.p. dose of normal saline (0.9%) on day 8. (III) MTX group: Rats received an equivalent volume of 1% CMC daily for 10 days and a single MTX dose (20 mg/kg, i.p.) on day 8. (IV) QUR + MTX group: Rats were given QUR (20 mg/kg, p.o.) for 10 days and a single MTX dose on day 8. (V) IAA + MTX group: Rats were given IAA for 10 days and a single MTX dose on day 8. This figure was illustrated using (BioRender.com).

Thirty Wistar albino rats were randomly divided into 5 groups (six rats/group). Control group: Rats were given an equivalent volume of 1% CMC orally. IAA group: Rats were given IAA (40 mg/kg, p.o.) [40] for 10 days. MTX group: Rats received a single MTX dose (20 mg/kg, i.p.) [41] on day 8. QUR + MTX group: Rats were given QUR (20 mg/kg, p.o.) [42] for 10 days, and a single MTX dose on day 8. IAA + MTX group: Rats were given IAA for 10 days, and a single MTX dose on day 8 (Figure 8).

On day 11, all the rats were subjected to a gradual increase in the concentration of carbon dioxide (CO_2_) and then sacrificed via decapitation. Blood samples were collected, centrifuged to obtain clear sera, and then stored at −80 °C. Liver tissues were rapidly dissected and washed with ice-cold phosphate-buffered saline (PBS). Liver tissue was cut and divided into parts; one part was immersed in 10% formalin for histopathological and immunohistochemical analyses. The remaining parts were homogenized (20% *w*/*v*) in normal saline and centrifuged at 3000 rpm for 30 min. The supernatants were collected and stored at −80 °C for further analyses.

### 4.3. Liver Function Tests in Serum

The serum activity levels of ALT and AST were measured spectrophotometrically using standard diagnostic kits and calculated utilizing specific formula according to the manufacturer’s instructions.

### 4.4. Examination of Oxidative Stress in Liver Tissue

The hepatic levels of malondialdehyde (MDA) were examined spectrophotometrically by measuring a component of thiobarbituric acid reactive substances (TBARSs) according to the method of Ohkawa [43]. Reduced glutathione (GSH) levels were determined by using 5, 5′-dithio-bis -2-nitrobenzoic acid (DTNB) (Ellman’s reagent). This assay is based on the reaction between GSH and DTNB, which produces an oxidized glutathione–TNB adduct (GS-TNB). The formation of TNB is proportional to the concentration of GSH in the liver sample [44]. The activity of superoxide dismutase (SOD) in the liver tissue was determined using the method of Marklund [45]. This assay is based on competitive action on the O_2_^−^ between pyrogallol and SOD. Univalent SOD reduces oxygen to oxygen peroxide, as shown in the following chemical reaction: 2O_2_^−^ + 2H^+^ → O_2_ + H_2_O_2_.

### 4.5. Determination of Inflammatory Responses in Liver Tissue

The hepatic levels of TNF-α, IL-1β, and IL-6 were analyzed using specific ELISA kits, and these analyses were performed according to the manufacturer’s instructions. All markers’ optical densities were read at 450 nm using a Biotek microplate (Biotek, Winooski, VT, USA).

### 4.6. Histopathological and Immunohistochemical Studies

Parts of the liver tissue samples were cut and fixed in 10% formalin, processed using a tissue processor to create paraffin blocks, and then cut into 5 μm thick sections using a microtome. The sections were deparaffinized using xylene for 15 min and then rehydrated using a decreasing concentration of ethanol and distilled water. To examine the histopathological change in the liver tissue samples, the sections were stained with hematoxylin and eosin (H&E). Images of the stained samples were captured using light microscopy.

An immunohistochemical technique was used to detect the immunoreactivity of TLR4, NF-κB, and caspase-3 targets in the paraffin-embedded slides. The liver slices were subjected to dewaxing, antigen retrieval, staining with primary antibodies, and then incubation with the secondary antibody. Then, the slices were counterstained using hematoxylin, and their immunoreactivities were visualized using an Optika microscope (Ponteranica, Italy).

### 4.7. Statistical Analysis

Statistical comparisons between the groups were performed using a one-way analysis of variance (ANOVA) followed by the Tukey–Kramer multiple comparisons test. The data were analyzed using GraphPad Prism 9 software (San Diego, CA, USA). These data were expressed as means ± standard error of the mean (SEM), and differences were considered significant at *p* < 0.05. Quantitative analyses of the histopathological and immunological examinations were performed using photographed pictures (×400 microscopic magnification) and ImageJ software (46-bit Java 8), respectively.

## 5. Conclusions

**Figure 9:**
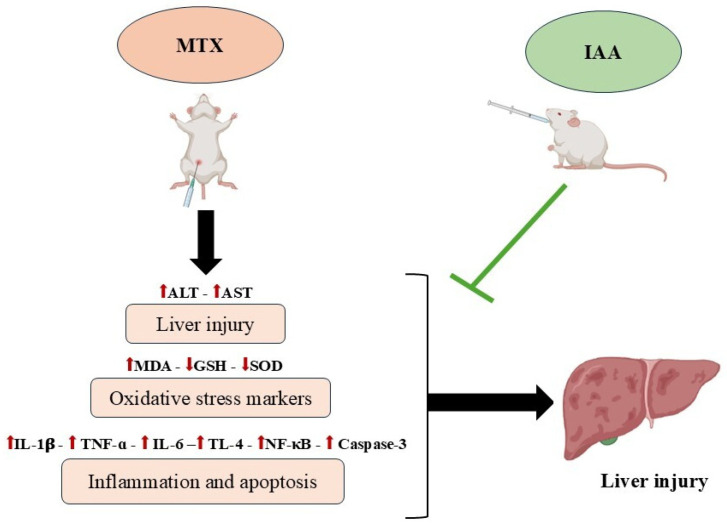
Summary of the protective mechanisms of IAA against MTX-induced liver injury.

Collectively, our results showed that IAA modulated the toxic effects of MTX (Figure 9). The hepatoprotective effects from IAA were achieved through the attenuation of oxidative stress, inflammation, and apoptosis mechanisms, as well as improvements in the morphological architecture of liver tissues. The results revealed that the antioxidant effects of IAA occur via a reduction in the MDA level and an increase in the GSH and SOD activity levels. In addition, IAA modulated the inflammation and apoptosis caused by MTX by decreasing the levels of TLR4, NF-κB, TNF-α, IL-1β, IL-6, and caspase-3.

Taking these results together, IAA has a pronounced protective activity against MTX-induced liver injury comparable to QUR activity. Furthermore, we believe that the mechanism underlying these effects may be closely related to the modulation of the TLR4/NF-κB/caspase-3 signaling pathway. IAA could be used clinically during long-term MTX therapy to overcome liver complications.

## Data Availability

The original contributions presented in this study are included in the article/Supplementary Materials. Further inquiries can be directed to the corresponding authors.

## Supplementary Materials

The following supporting information can be downloaded at: https://www.mdpi.com/article/10.3390/ph18060828/s1. The original images of Histology and Immunohistochemistry figures.
