# Supplementary figures and images for: Indole-3-Acetic Acid: Promising Protective Agent Against Methotrexate-Induced Liver Injury via Modulation of TLR4/NF-κB/Caspase-3 Pathway (part 2 of 2)
Source: Pharmaceuticals (Basel). 2025 Jun 1;18(6):828. doi: 10.3390/ph18060828 (PMC12195934; doi:10.3390/ph18060828)

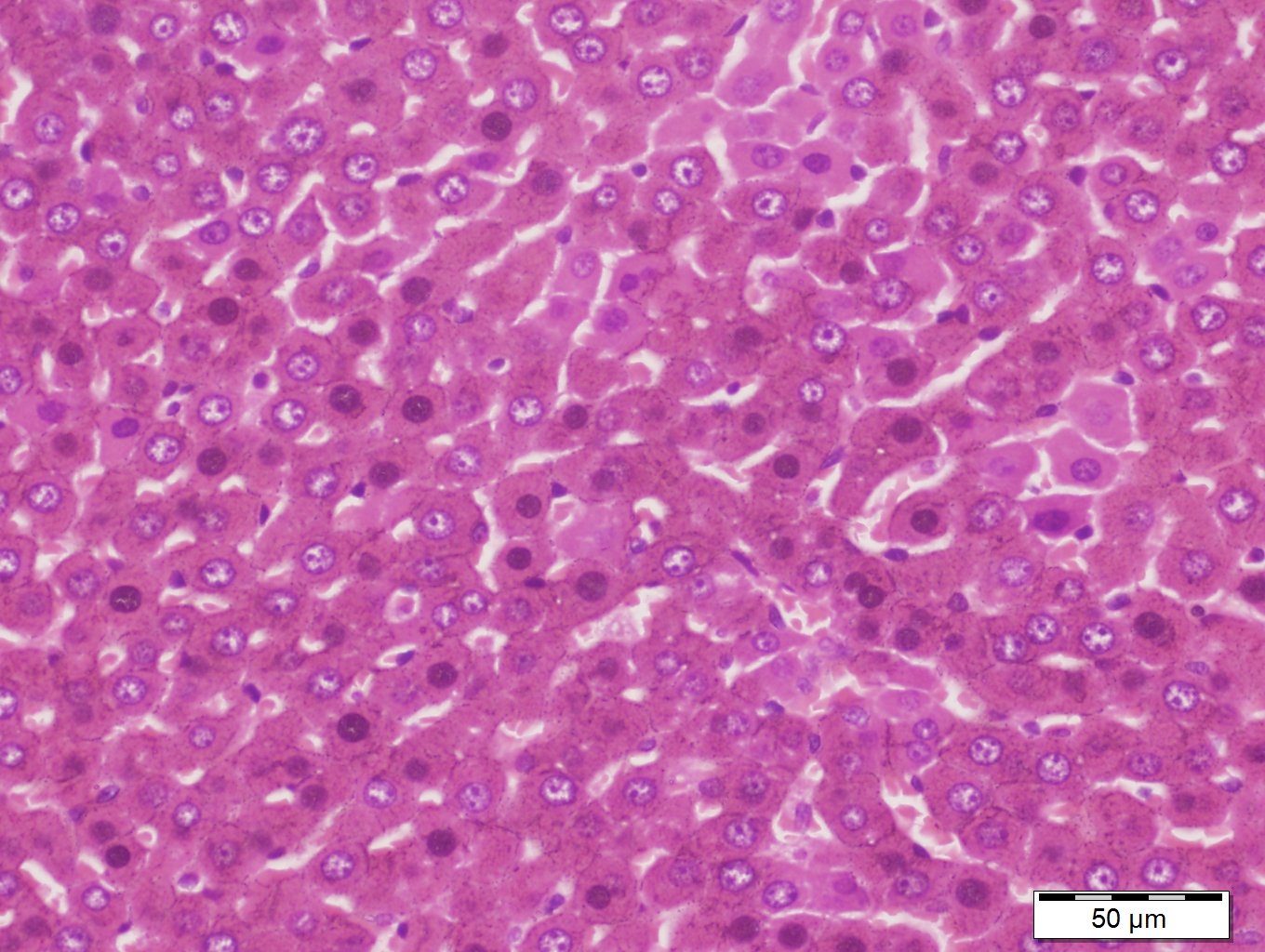

Supplement: Supplementary file 1 [file pharmaceuticals-18-00828-s001.zip › H&E and Immune images/Liver-H&E-Sumayya/Liver-MTX-H&E-X400-2 .jpg]

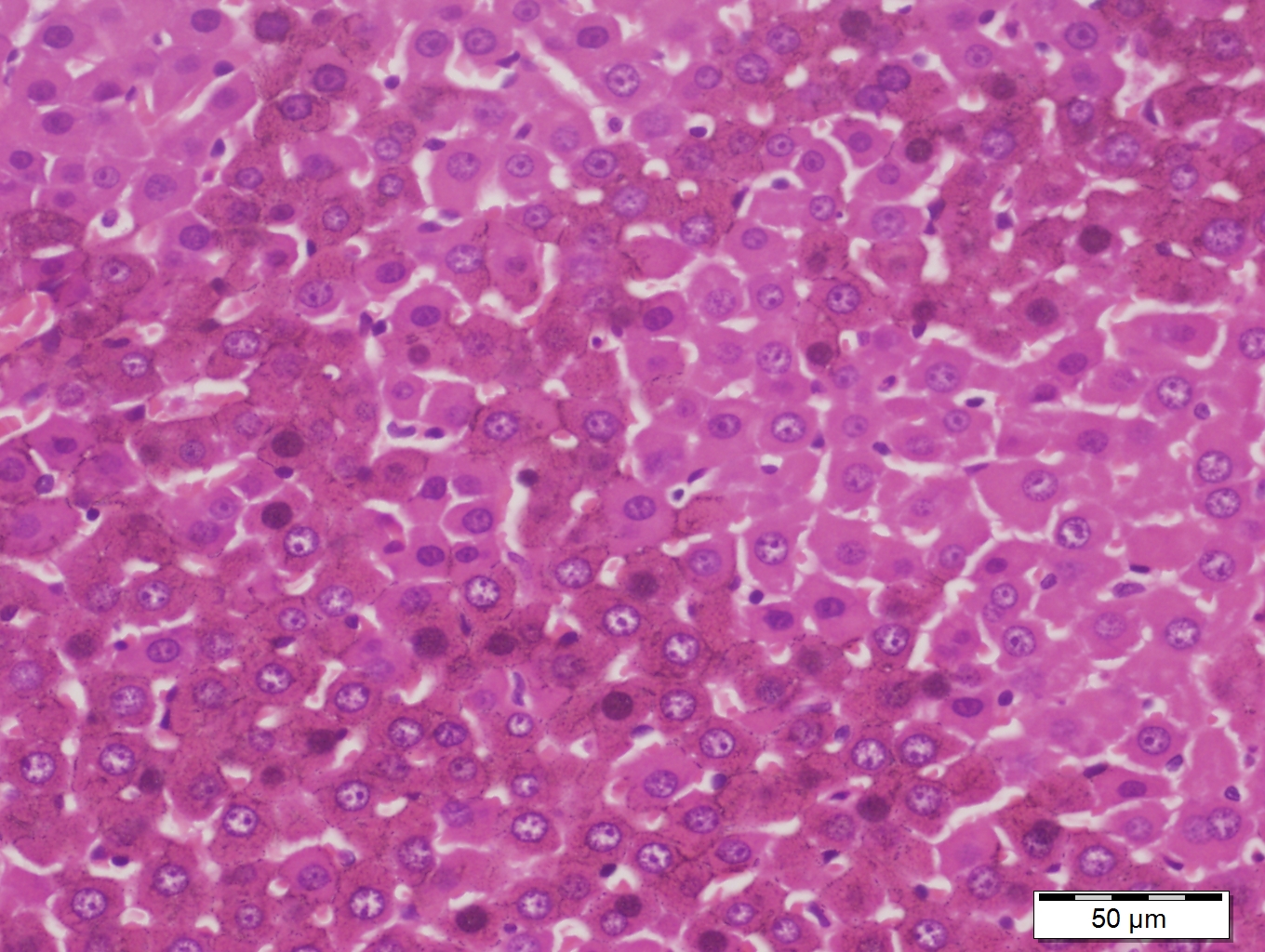

Supplement: Supplementary file 1 [file pharmaceuticals-18-00828-s001.zip › H&E and Immune images/Liver-H&E-Sumayya/Liver-MTX-H&E-X400-3 .jpg]

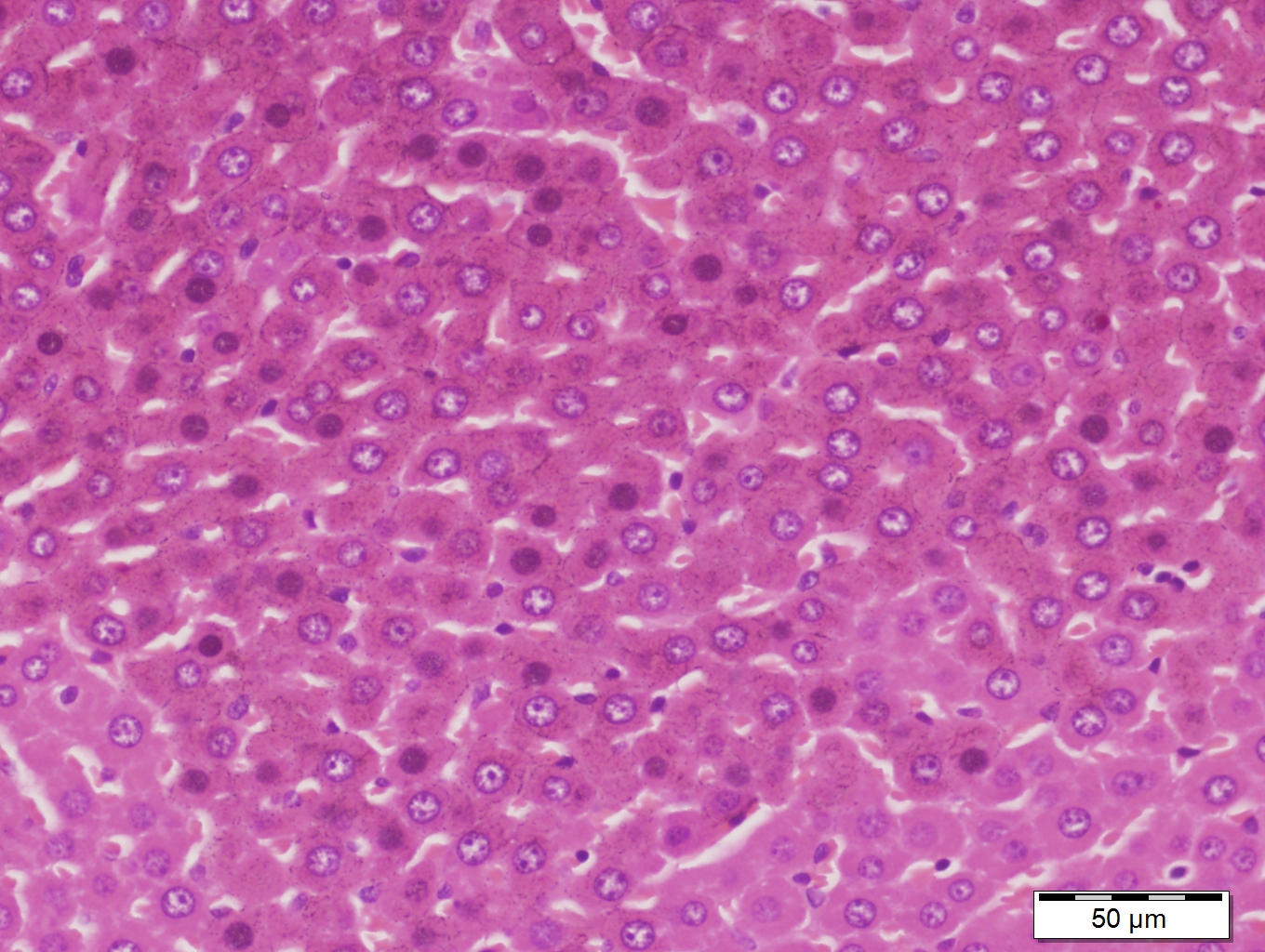

Supplement: Supplementary file 1 [file pharmaceuticals-18-00828-s001.zip › H&E and Immune images/Liver-H&E-Sumayya/Liver-MTX-H&E-X400-4 .jpg]

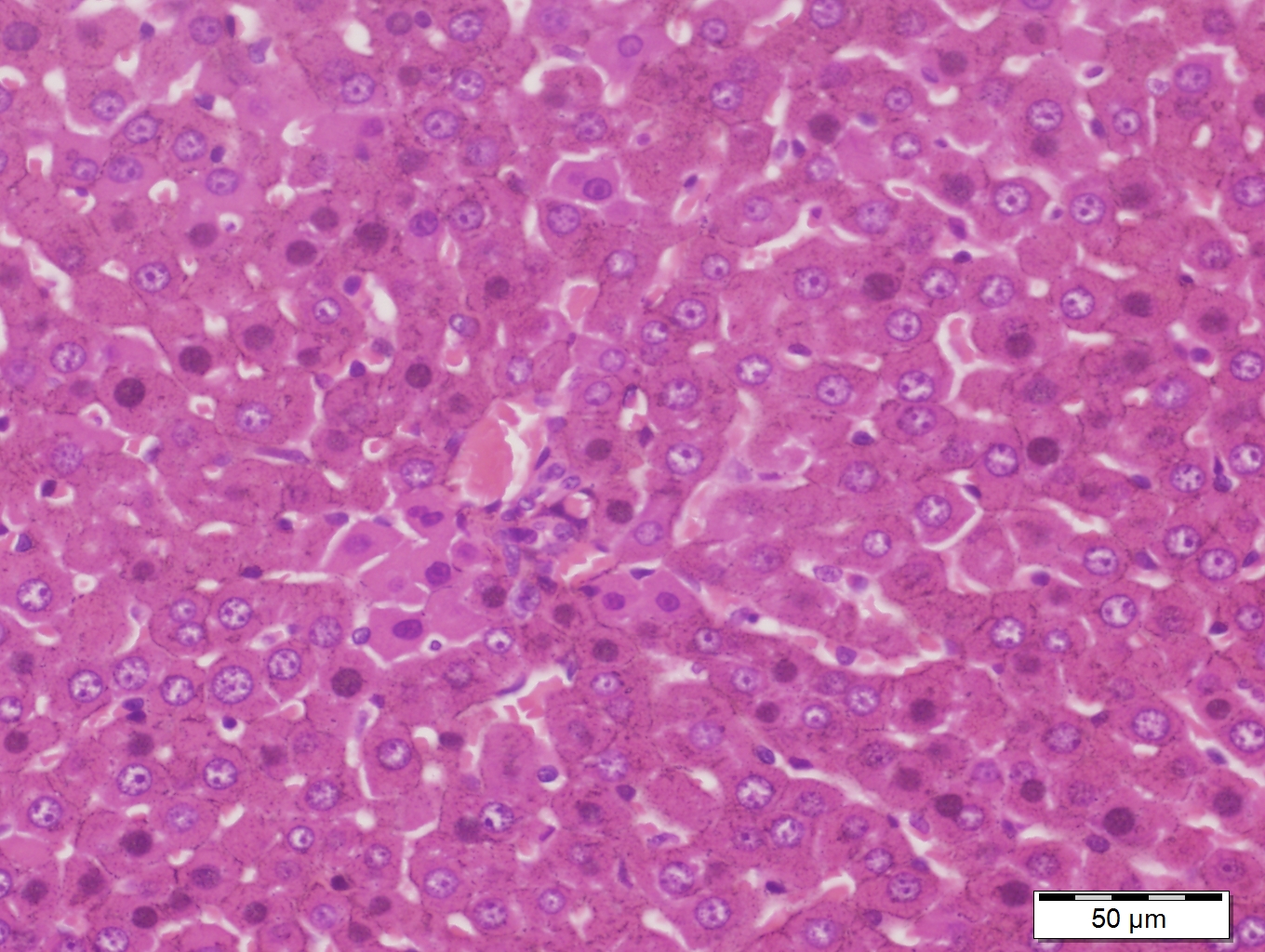

Supplement: Supplementary file 1 [file pharmaceuticals-18-00828-s001.zip › H&E and Immune images/Liver-H&E-Sumayya/Liver-MTX-H&E-X400-5 .jpg]

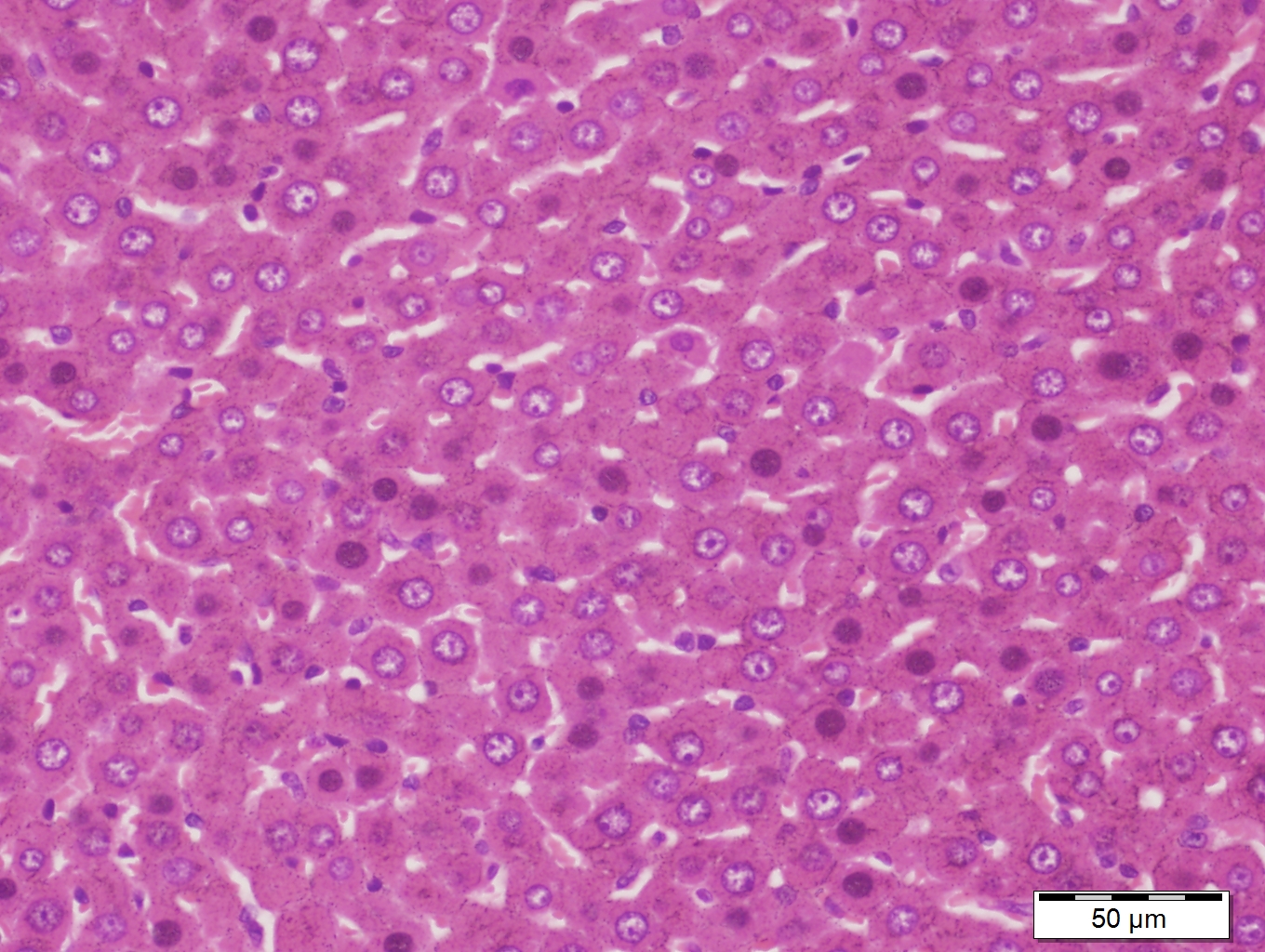

Supplement: Supplementary file 1 [file pharmaceuticals-18-00828-s001.zip › H&E and Immune images/Liver-H&E-Sumayya/Liver-MTX-H&E-X400-6 .jpg]

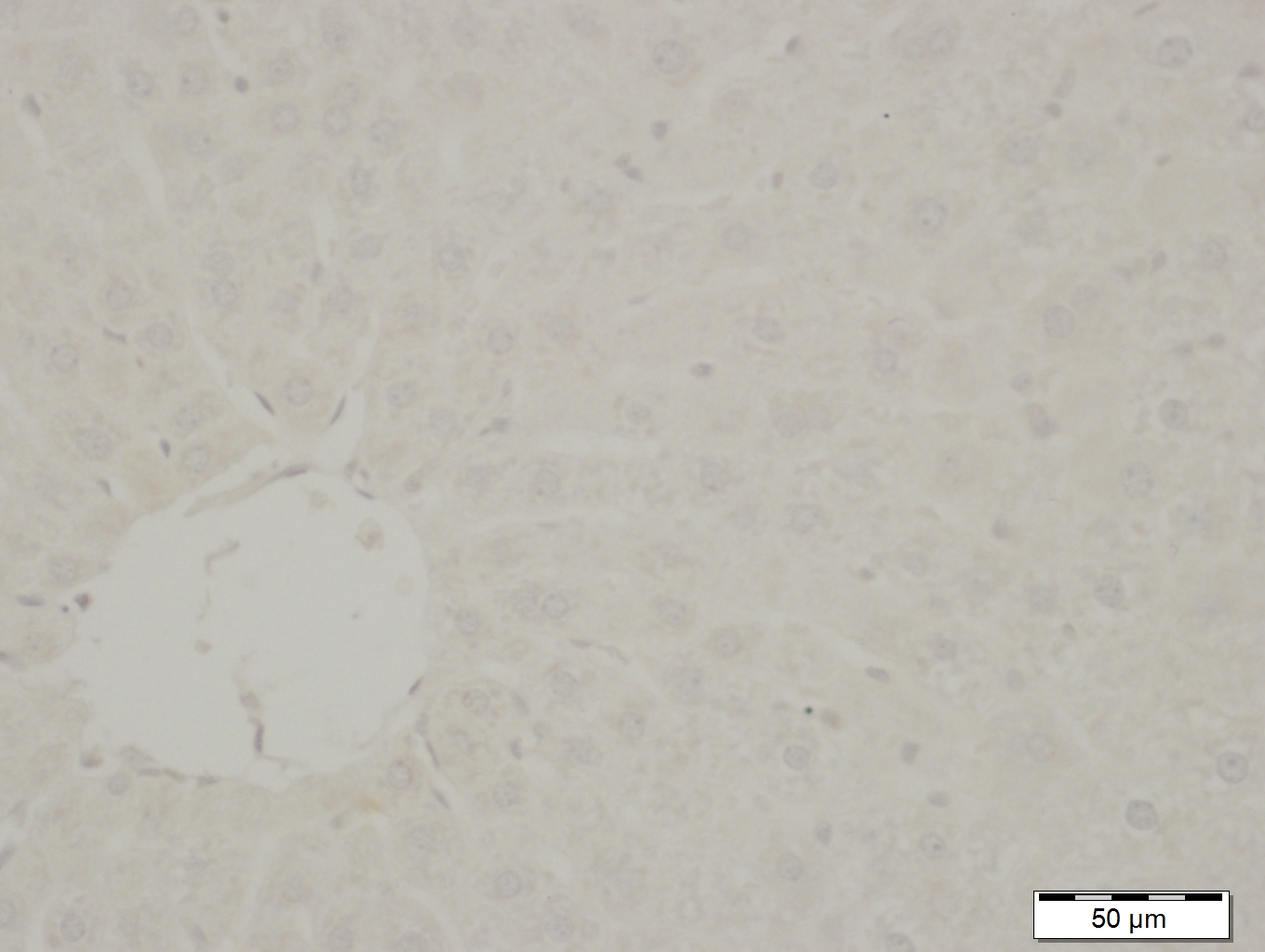

Supplement: Supplementary file 1 [file pharmaceuticals-18-00828-s001.zip › H&E and Immune images/Liver-TLR4-Sumayya/Liver-Cont-TL-X400-1 .jpg]

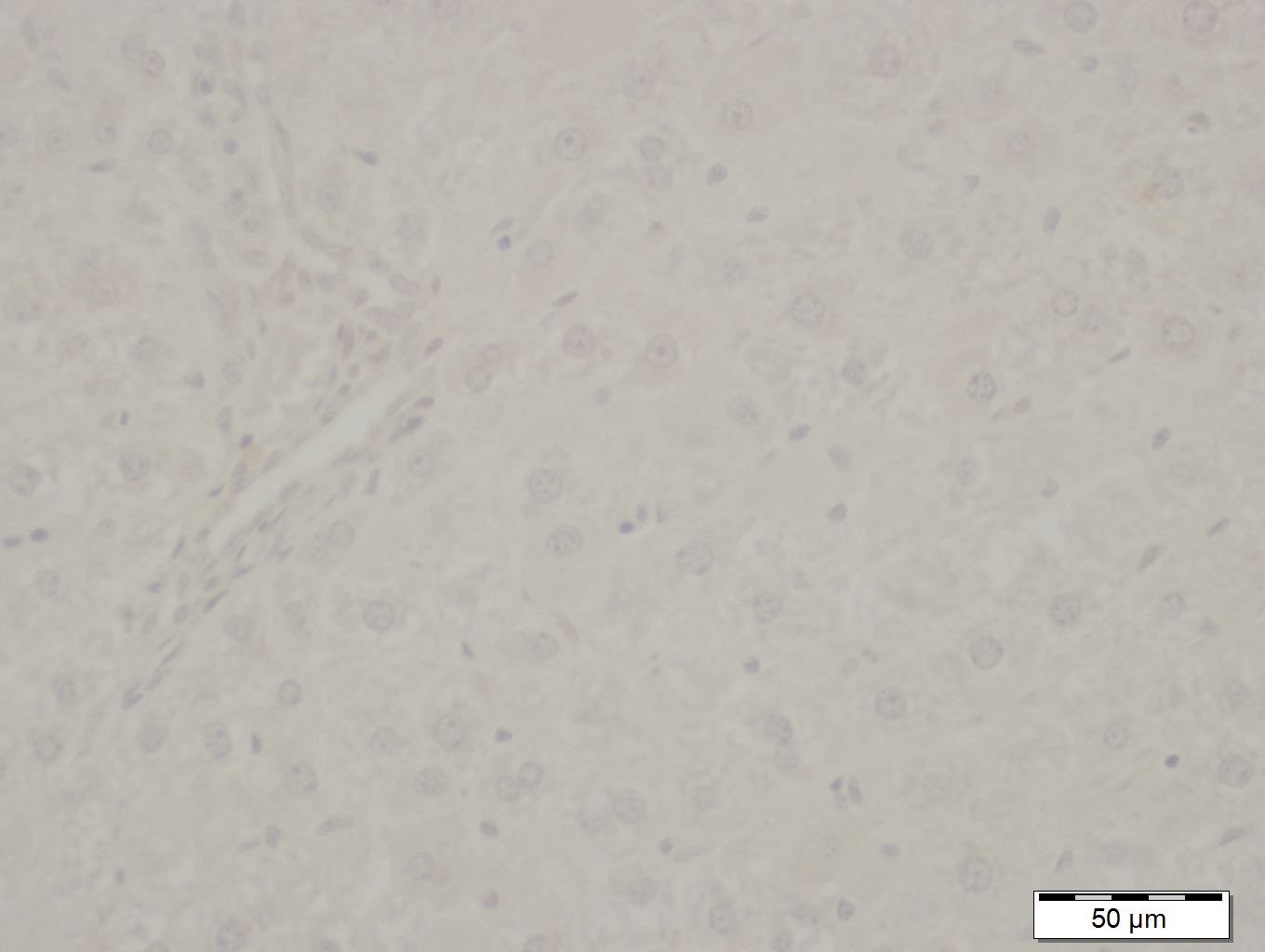

Supplement: Supplementary file 1 [file pharmaceuticals-18-00828-s001.zip › H&E and Immune images/Liver-TLR4-Sumayya/Liver-Cont-TL-X400-2 .jpg]

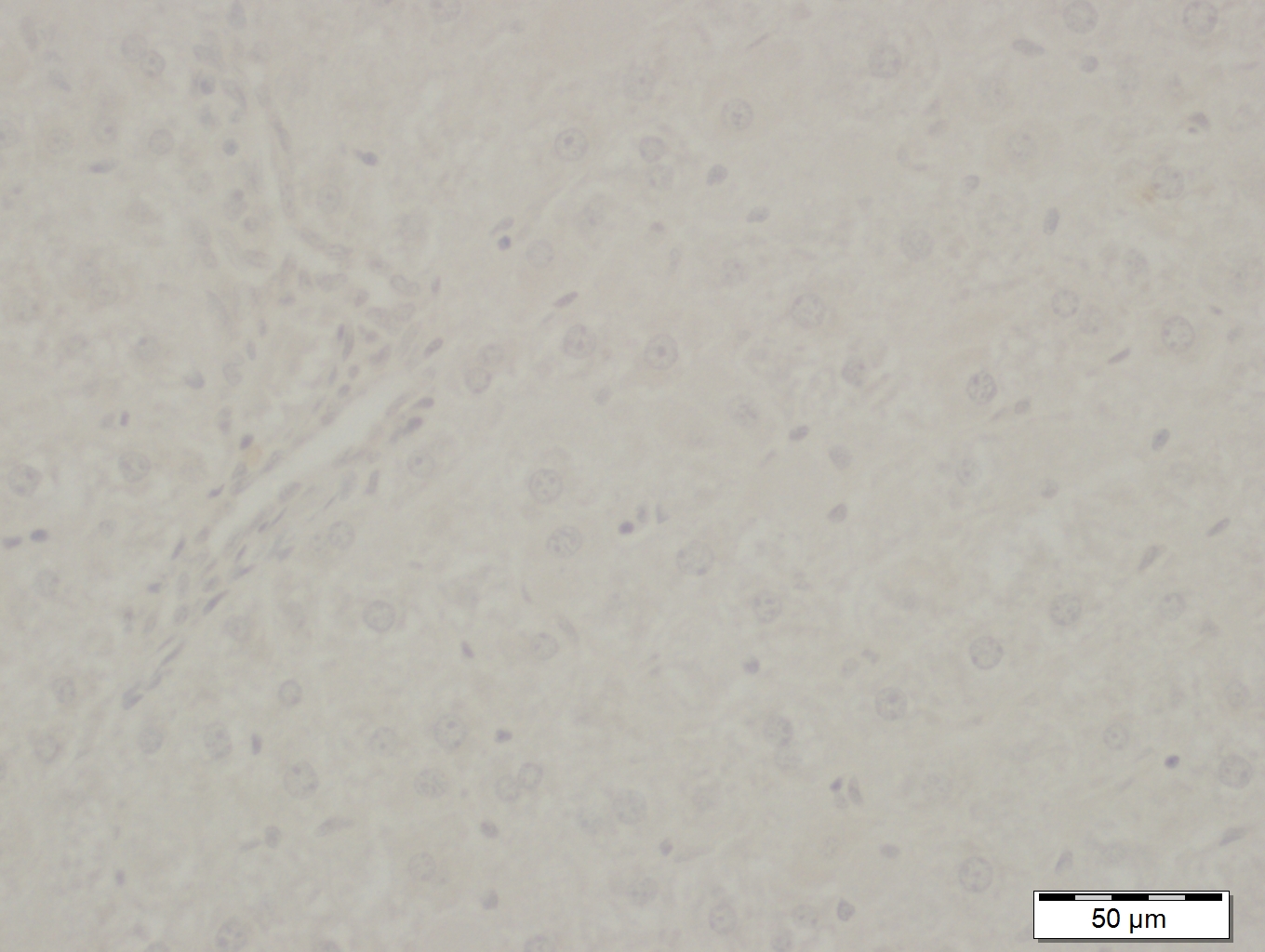

Supplement: Supplementary file 1 [file pharmaceuticals-18-00828-s001.zip › H&E and Immune images/Liver-TLR4-Sumayya/Liver-Cont-TL-X400-3 .jpg]

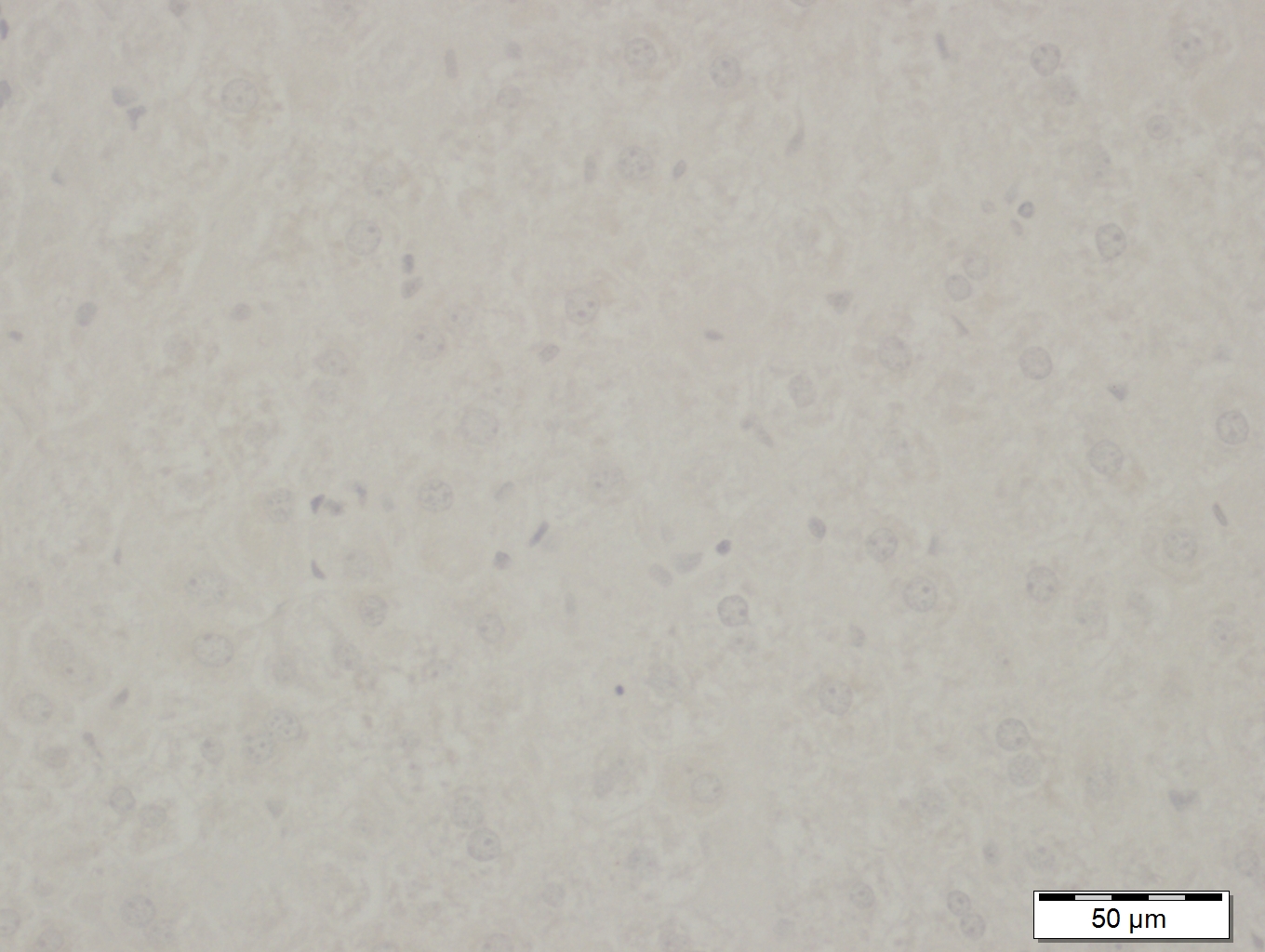

Supplement: Supplementary file 1 [file pharmaceuticals-18-00828-s001.zip › H&E and Immune images/Liver-TLR4-Sumayya/Liver-Cont-TL-X400-4 .jpg]

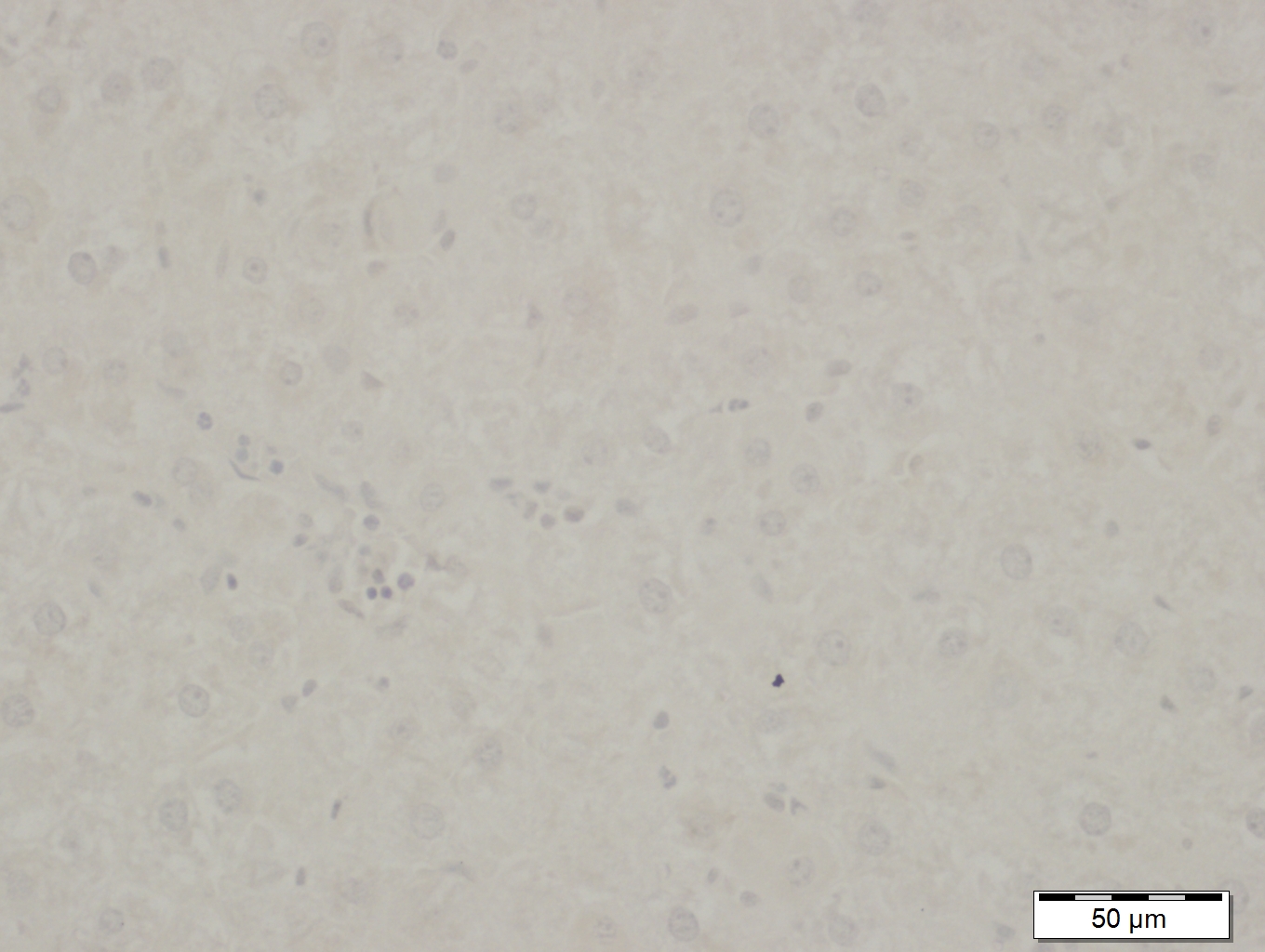

Supplement: Supplementary file 1 [file pharmaceuticals-18-00828-s001.zip › H&E and Immune images/Liver-TLR4-Sumayya/Liver-Cont-TL-X400-6 .jpg]

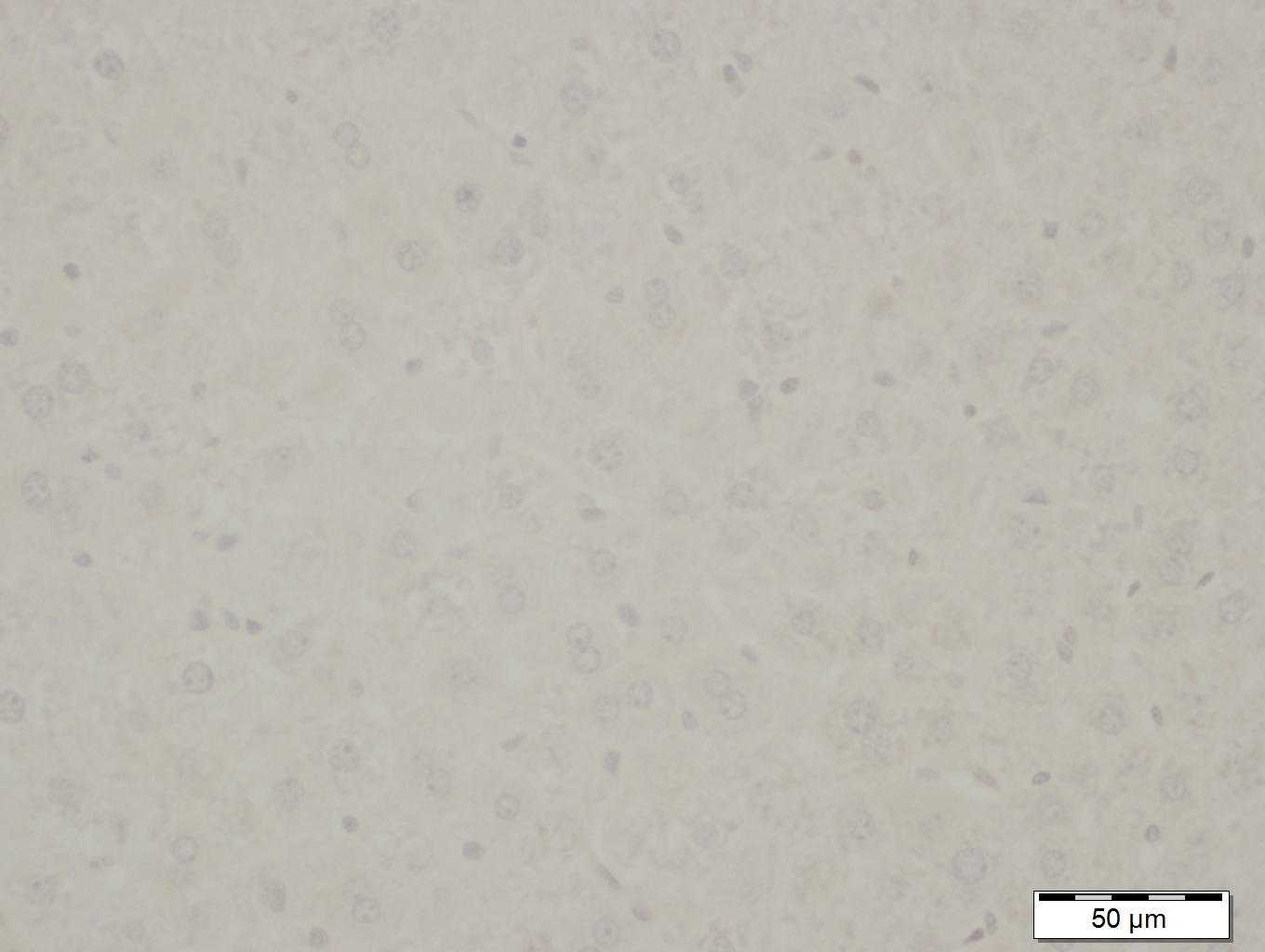

Supplement: Supplementary file 1 [file pharmaceuticals-18-00828-s001.zip › H&E and Immune images/Liver-TLR4-Sumayya/Liver-Cont-TL-X400-7 .jpg]

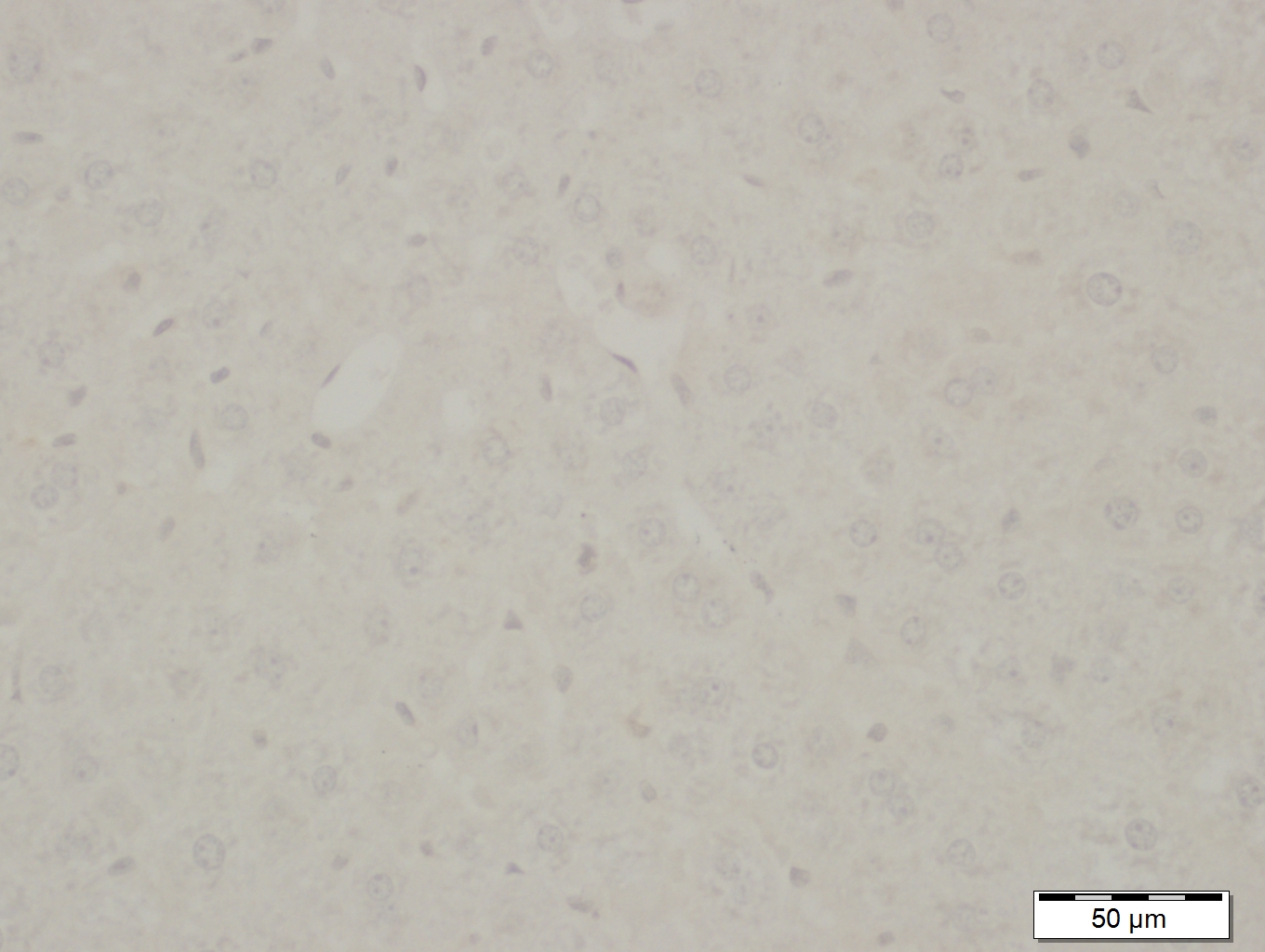

Supplement: Supplementary file 1 [file pharmaceuticals-18-00828-s001.zip › H&E and Immune images/Liver-TLR4-Sumayya/Liver-Cont-TL-X400-8 .jpg]

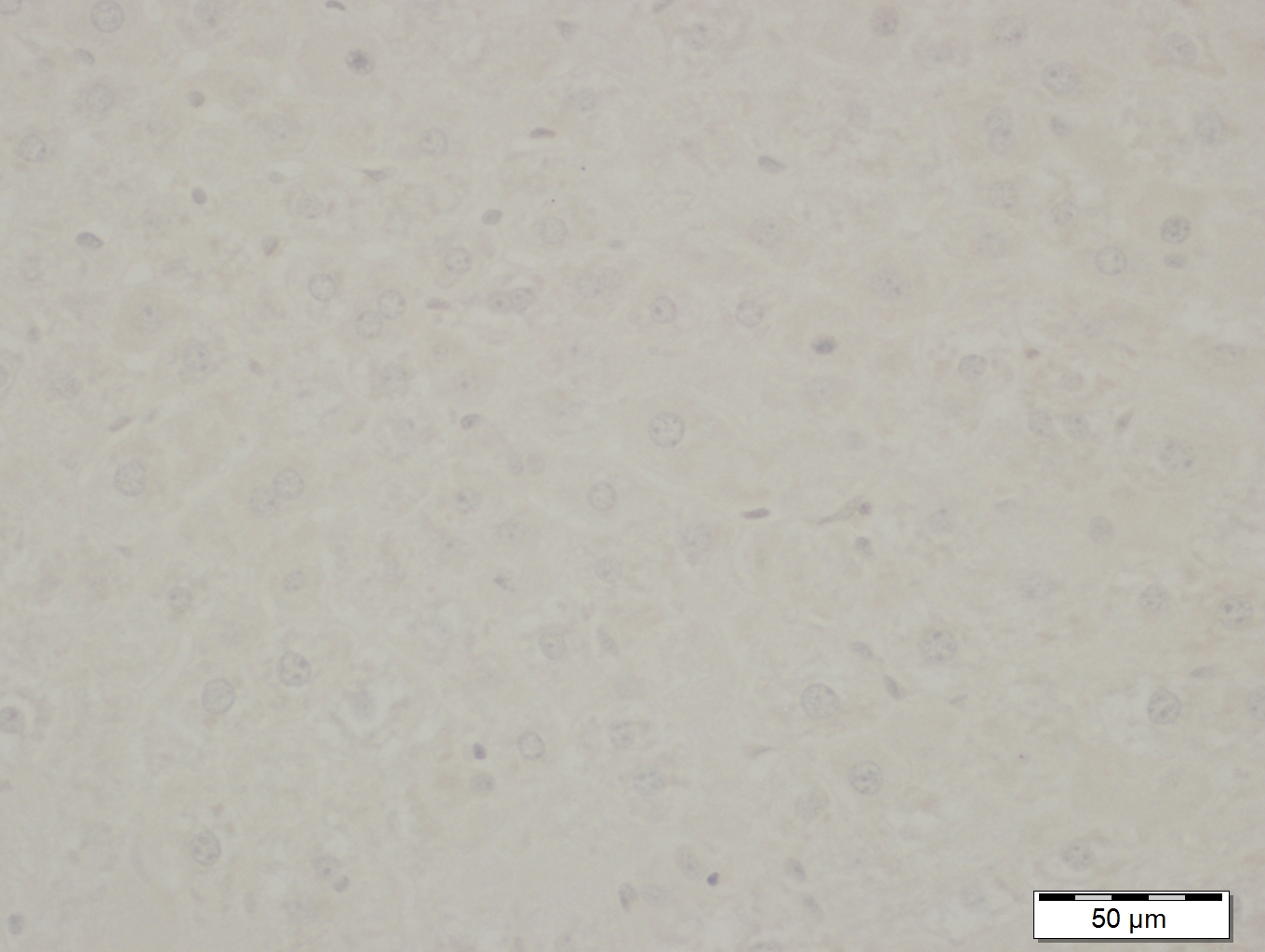

Supplement: Supplementary file 1 [file pharmaceuticals-18-00828-s001.zip › H&E and Immune images/Liver-TLR4-Sumayya/Liver-Cont-TL-X400-9 .jpg]

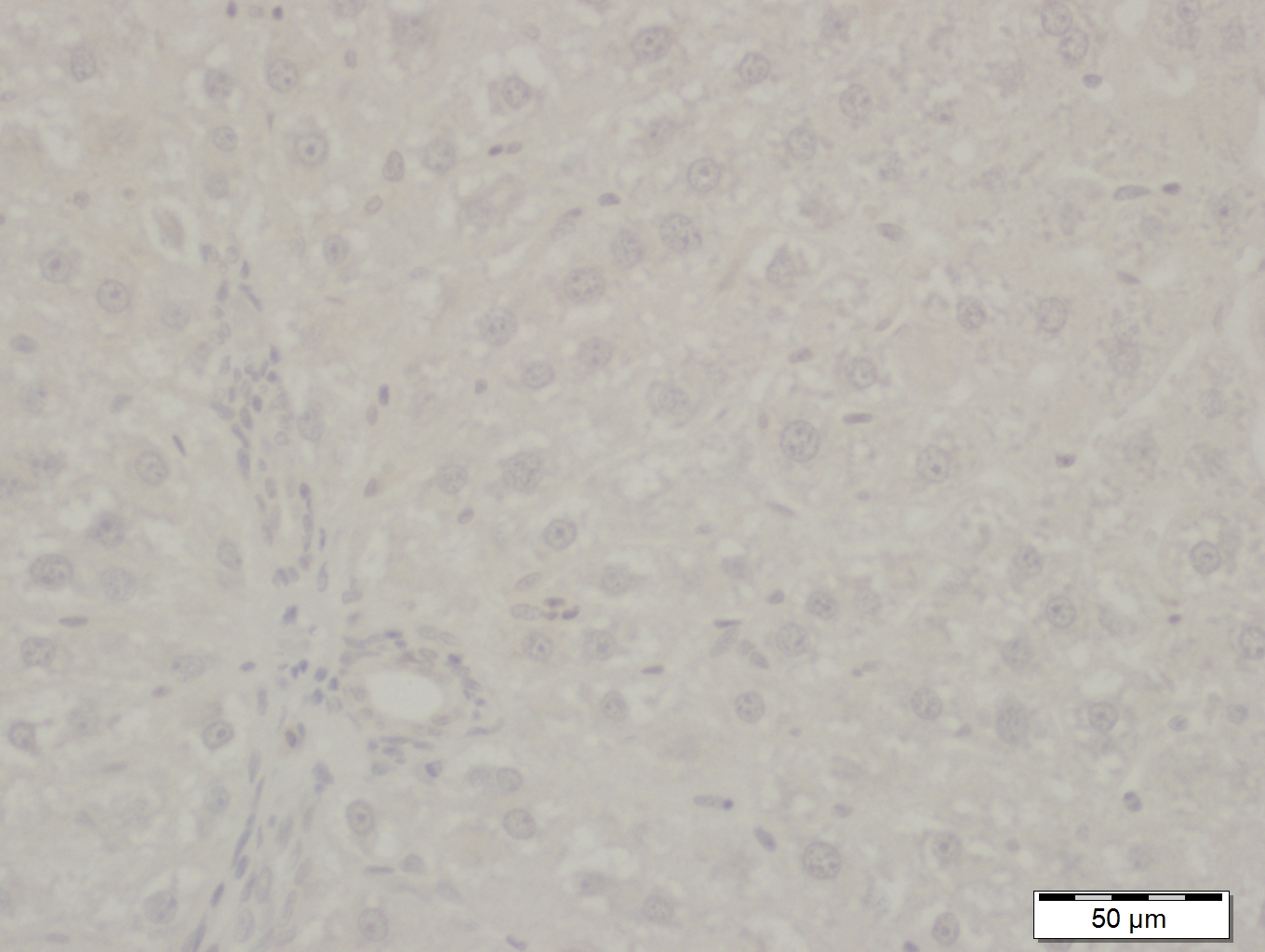

Supplement: Supplementary file 1 [file pharmaceuticals-18-00828-s001.zip › H&E and Immune images/Liver-TLR4-Sumayya/Liver-IAA-TL-X400-1 .jpg]

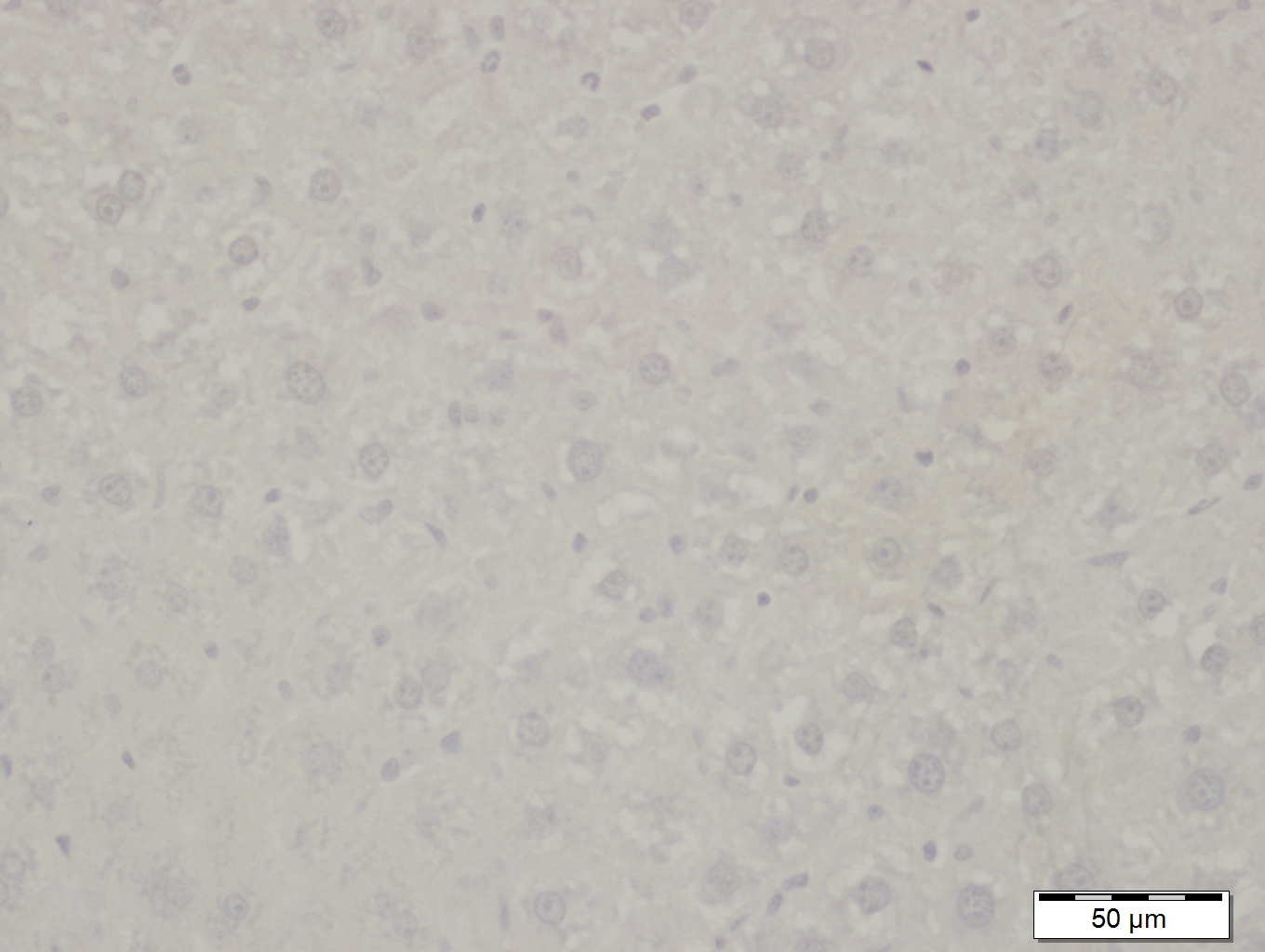

Supplement: Supplementary file 1 [file pharmaceuticals-18-00828-s001.zip › H&E and Immune images/Liver-TLR4-Sumayya/Liver-IAA-TL-X400-2 .jpg]

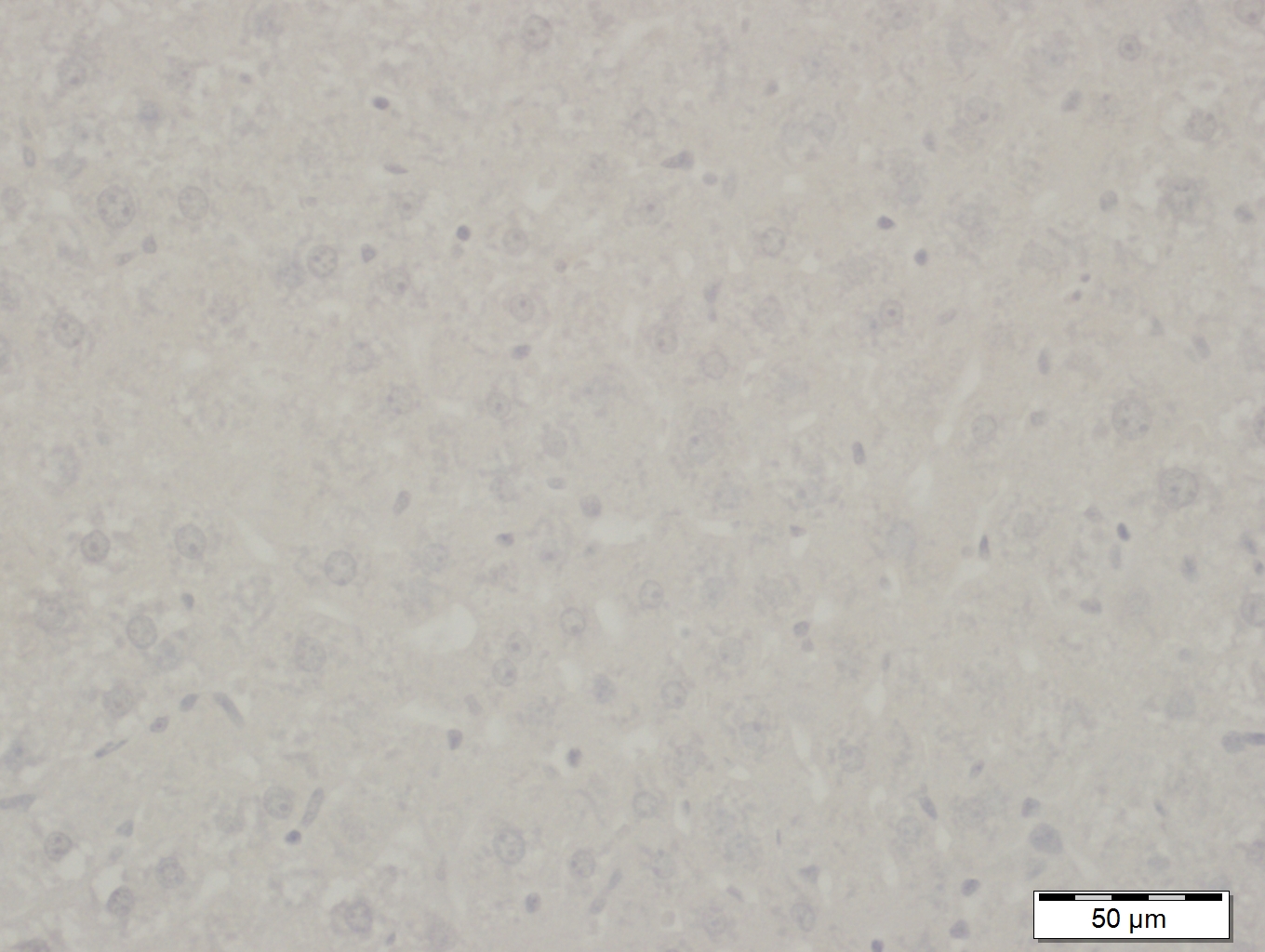

Supplement: Supplementary file 1 [file pharmaceuticals-18-00828-s001.zip › H&E and Immune images/Liver-TLR4-Sumayya/Liver-IAA-TL-X400-3 .jpg]

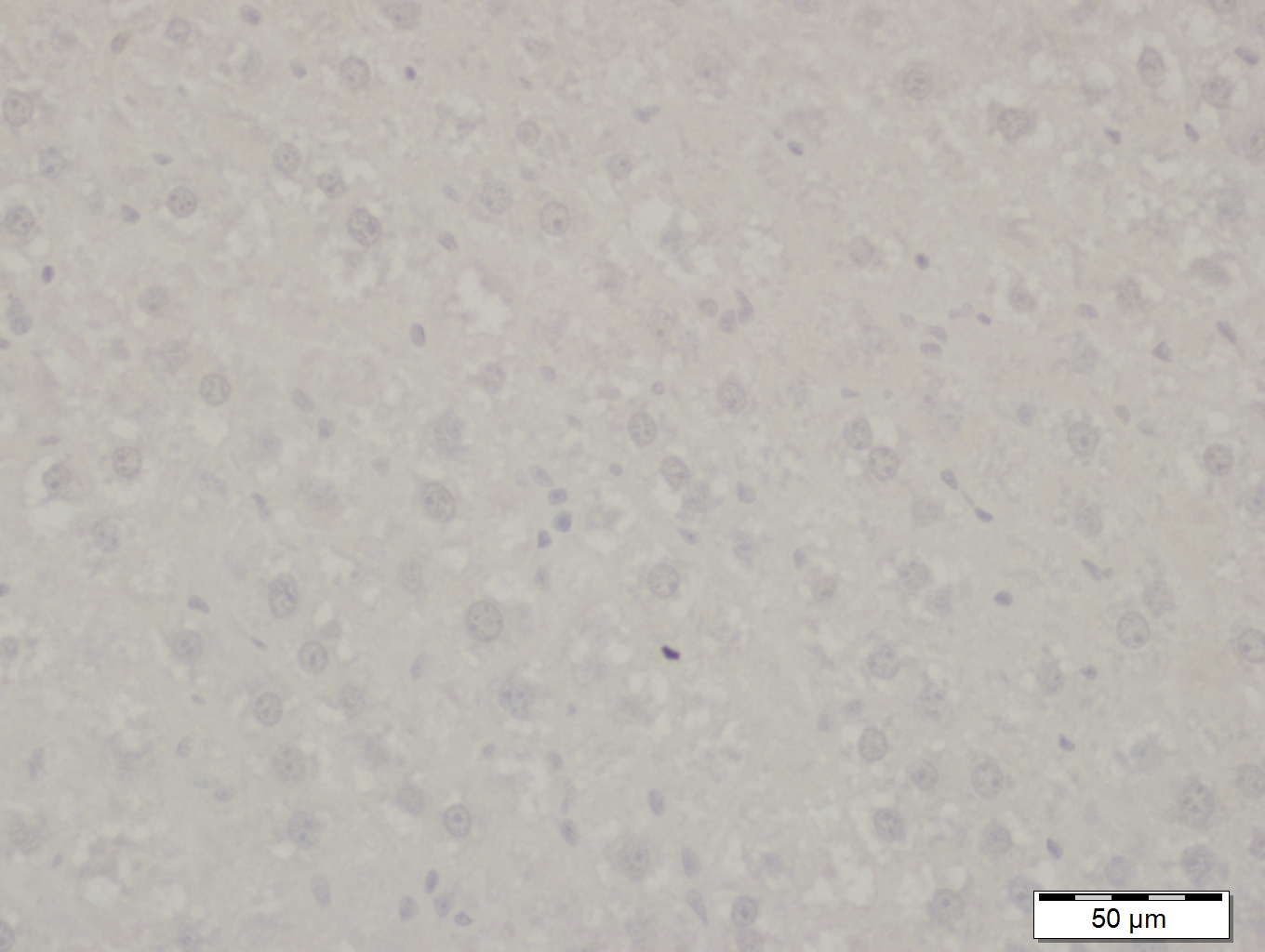

Supplement: Supplementary file 1 [file pharmaceuticals-18-00828-s001.zip › H&E and Immune images/Liver-TLR4-Sumayya/Liver-IAA-TL-X400-4 .jpg]

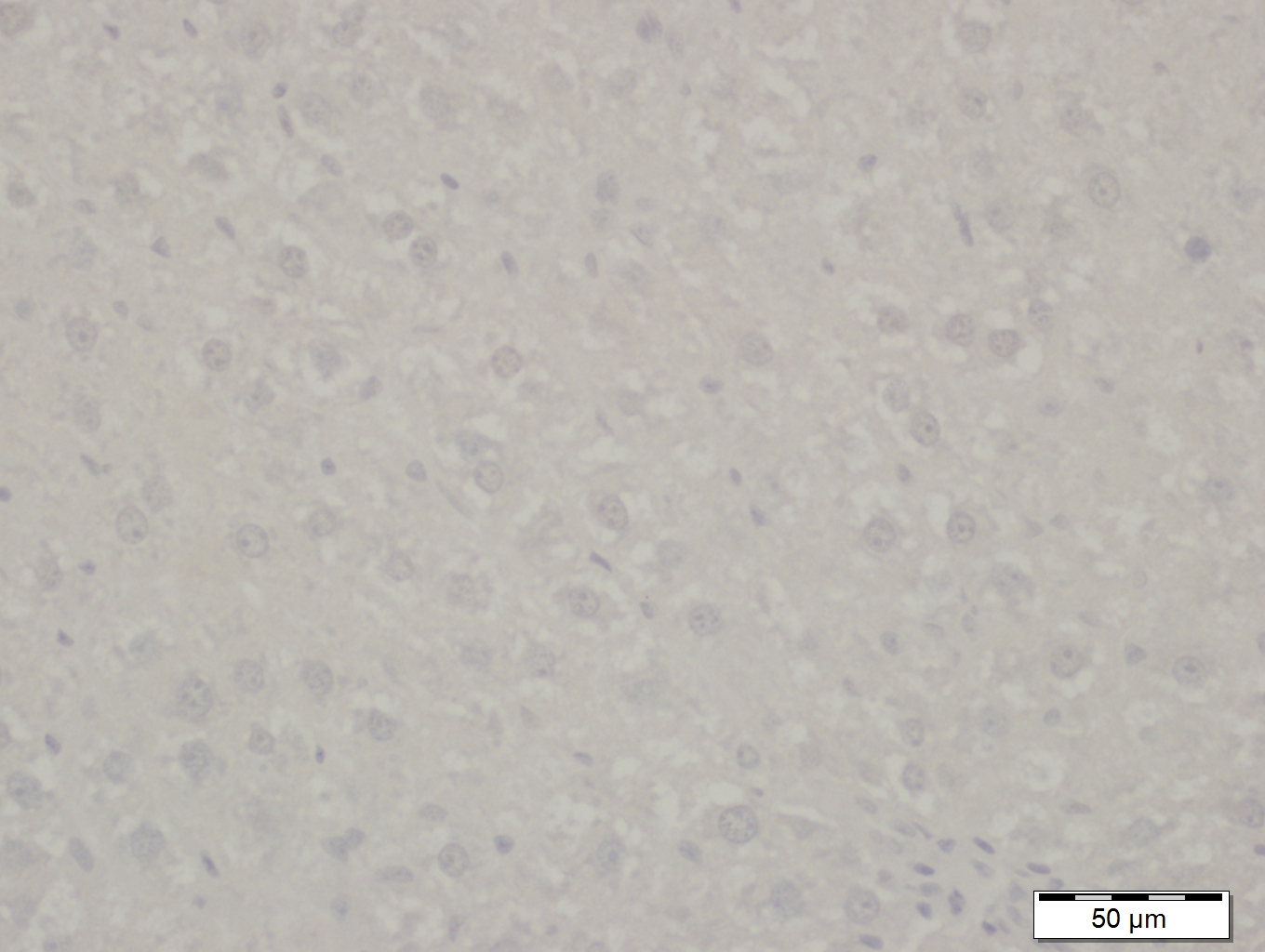

Supplement: Supplementary file 1 [file pharmaceuticals-18-00828-s001.zip › H&E and Immune images/Liver-TLR4-Sumayya/Liver-IAA-TL-X400-5 .jpg]

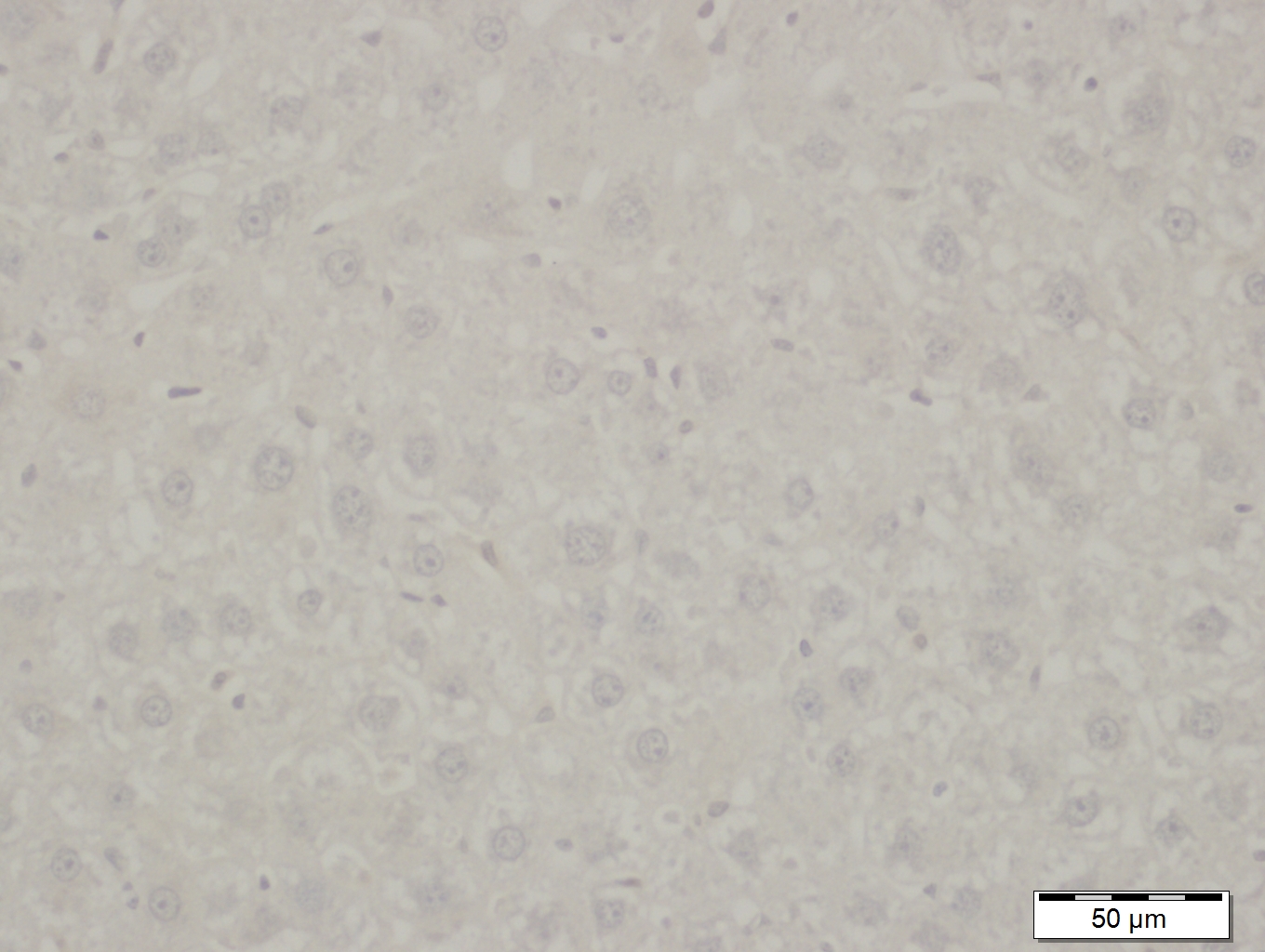

Supplement: Supplementary file 1 [file pharmaceuticals-18-00828-s001.zip › H&E and Immune images/Liver-TLR4-Sumayya/Liver-IAA-TL-X400-6 .jpg]

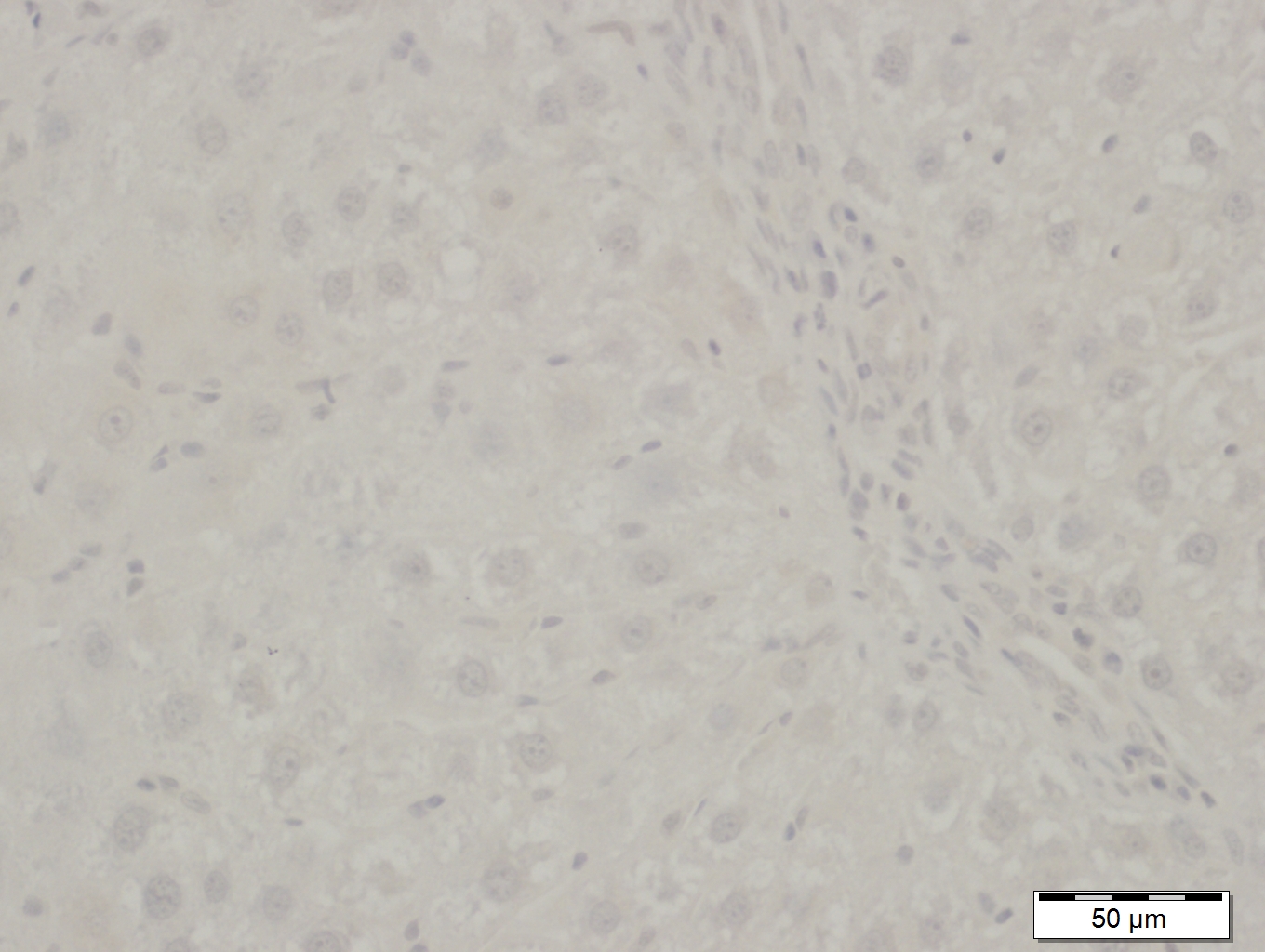

Supplement: Supplementary file 1 [file pharmaceuticals-18-00828-s001.zip › H&E and Immune images/Liver-TLR4-Sumayya/Liver-IAA-TL-X400-7 .jpg]

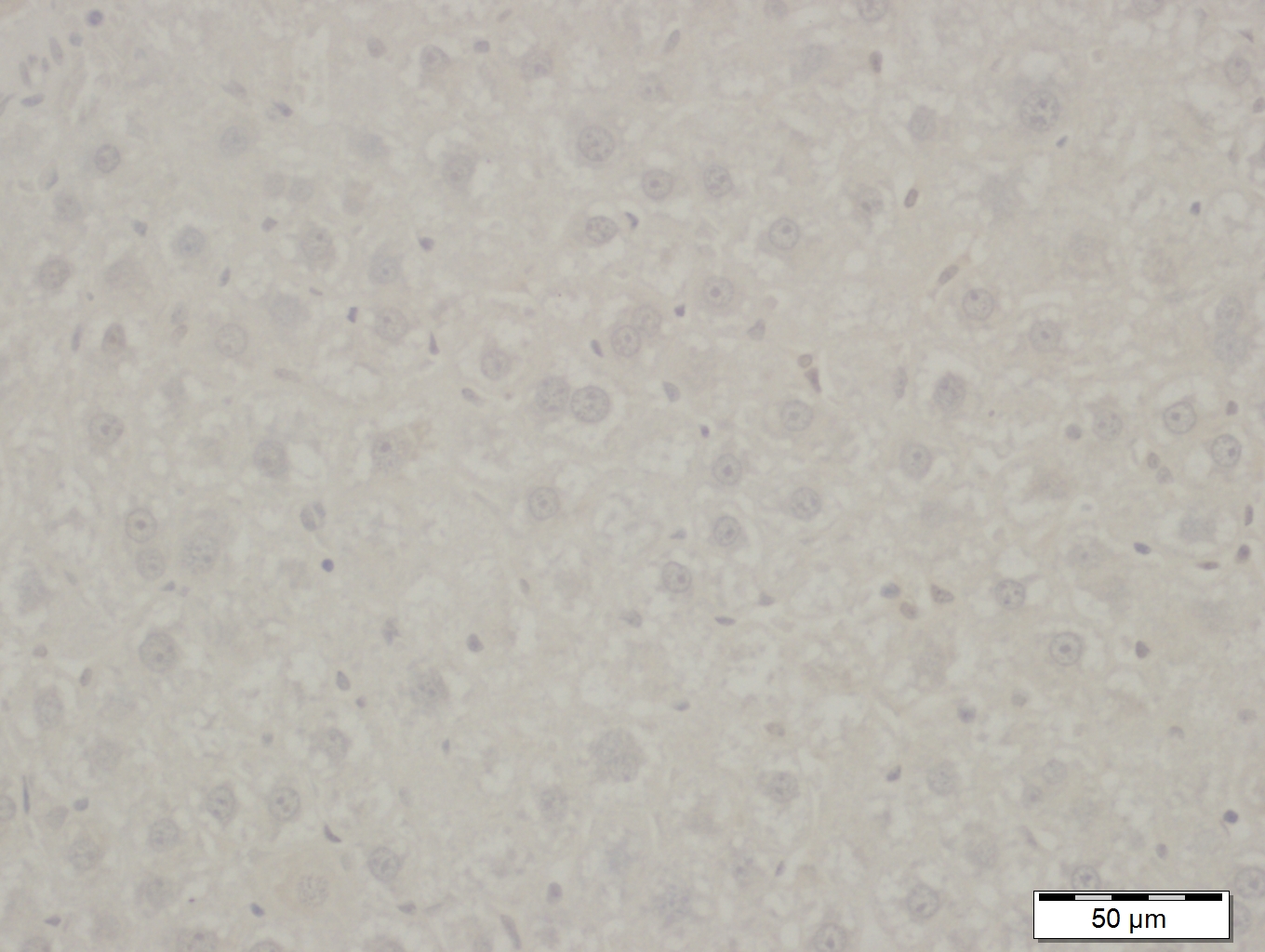

Supplement: Supplementary file 1 [file pharmaceuticals-18-00828-s001.zip › H&E and Immune images/Liver-TLR4-Sumayya/Liver-IAA-TL-X400-8 .jpg]

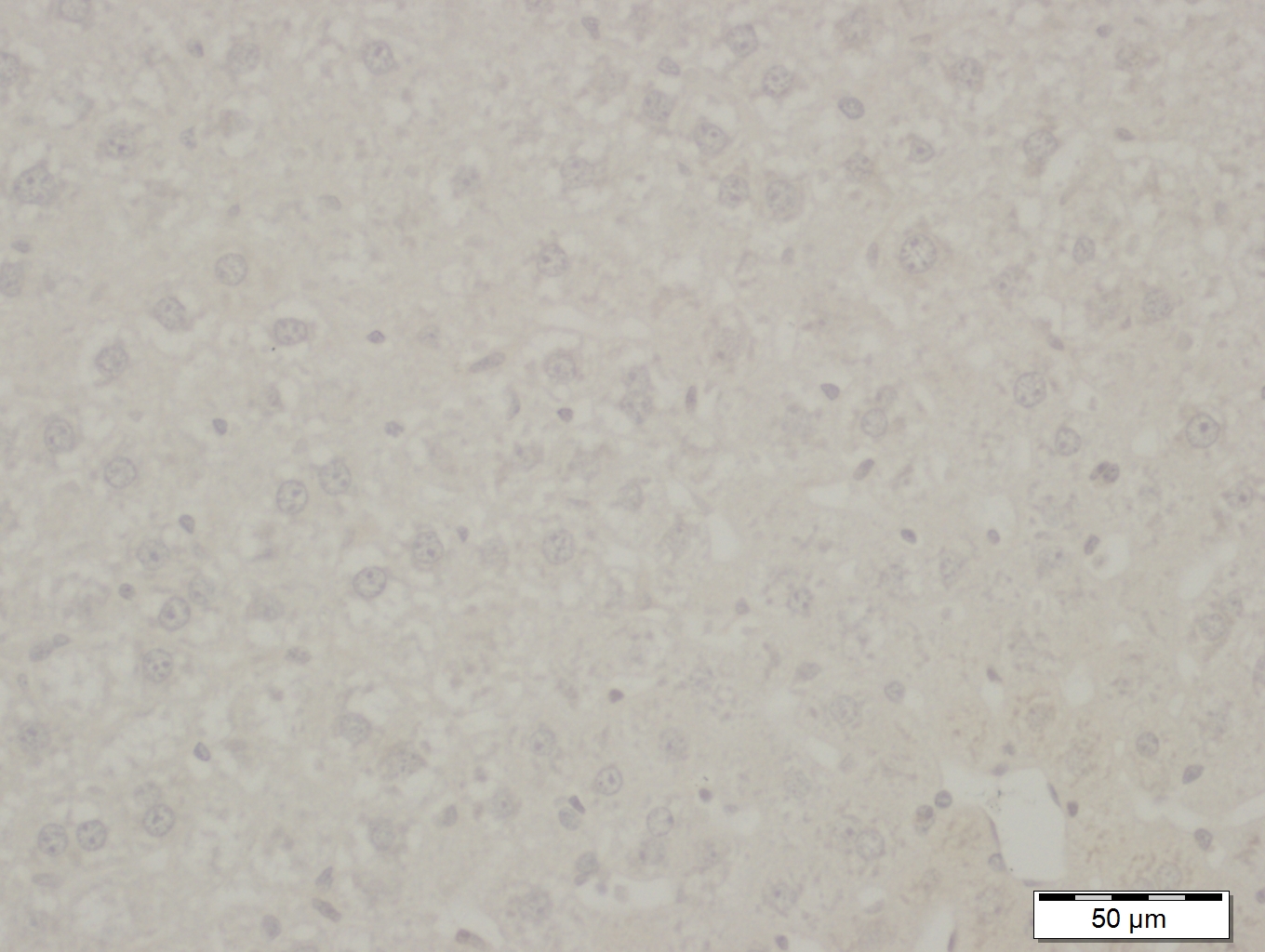

Supplement: Supplementary file 1 [file pharmaceuticals-18-00828-s001.zip › H&E and Immune images/Liver-TLR4-Sumayya/Liver-IAA-TL-X400-9 .jpg]

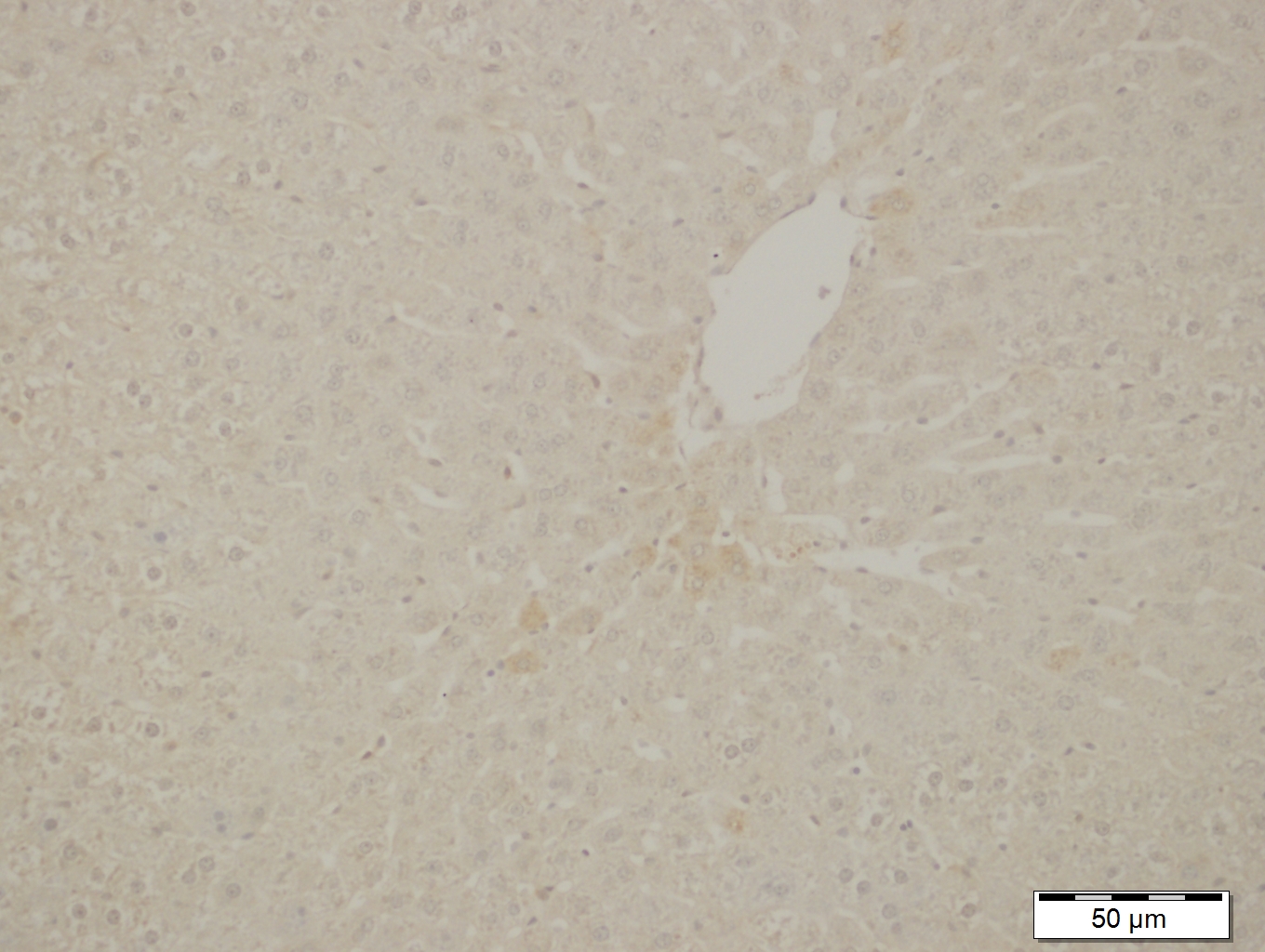

Supplement: Supplementary file 1 [file pharmaceuticals-18-00828-s001.zip › H&E and Immune images/Liver-TLR4-Sumayya/Liver-MTX+IAA-TL-X400-1 .jpg]

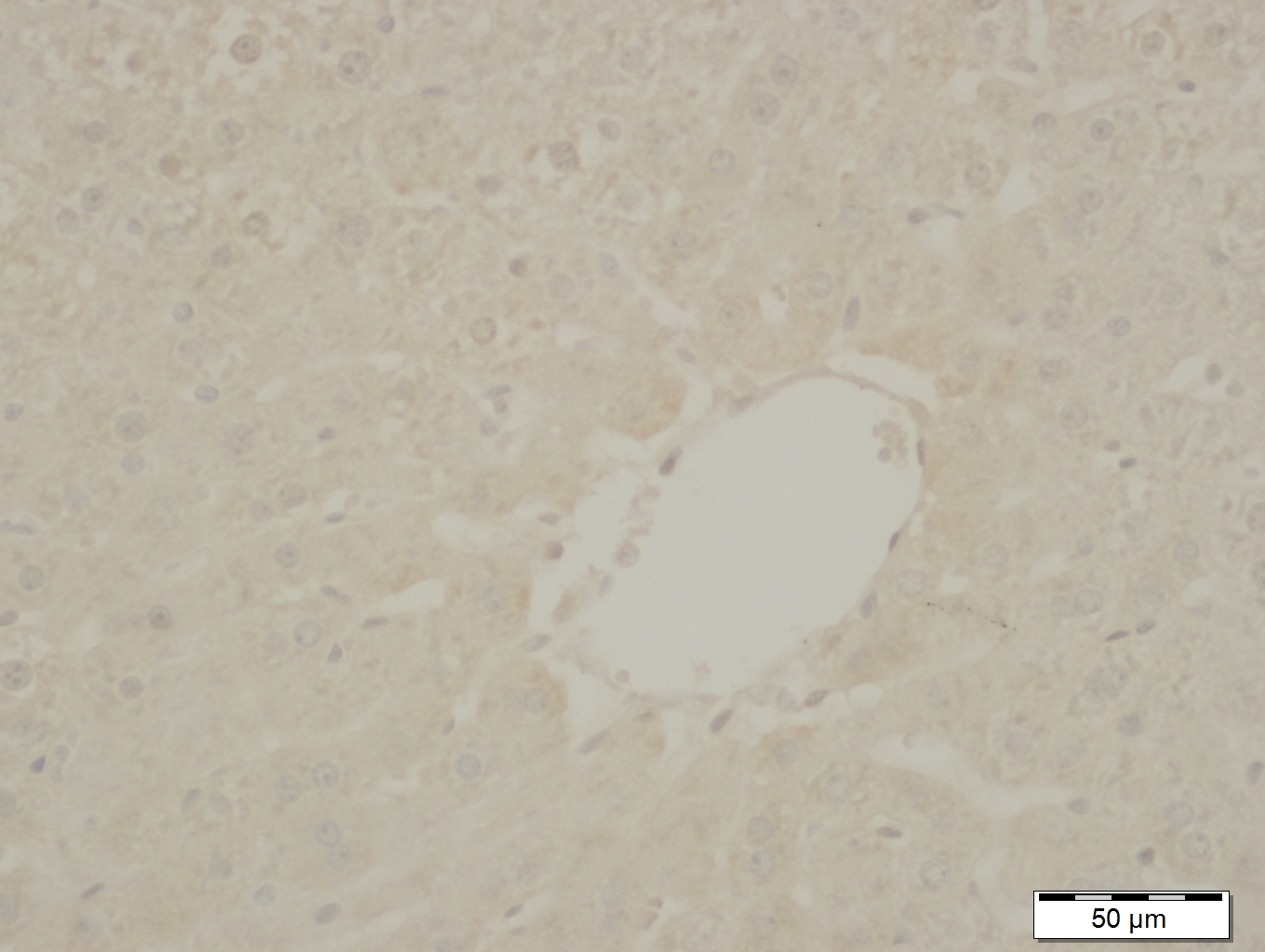

Supplement: Supplementary file 1 [file pharmaceuticals-18-00828-s001.zip › H&E and Immune images/Liver-TLR4-Sumayya/Liver-MTX+IAA-TL-X400-2 .jpg]

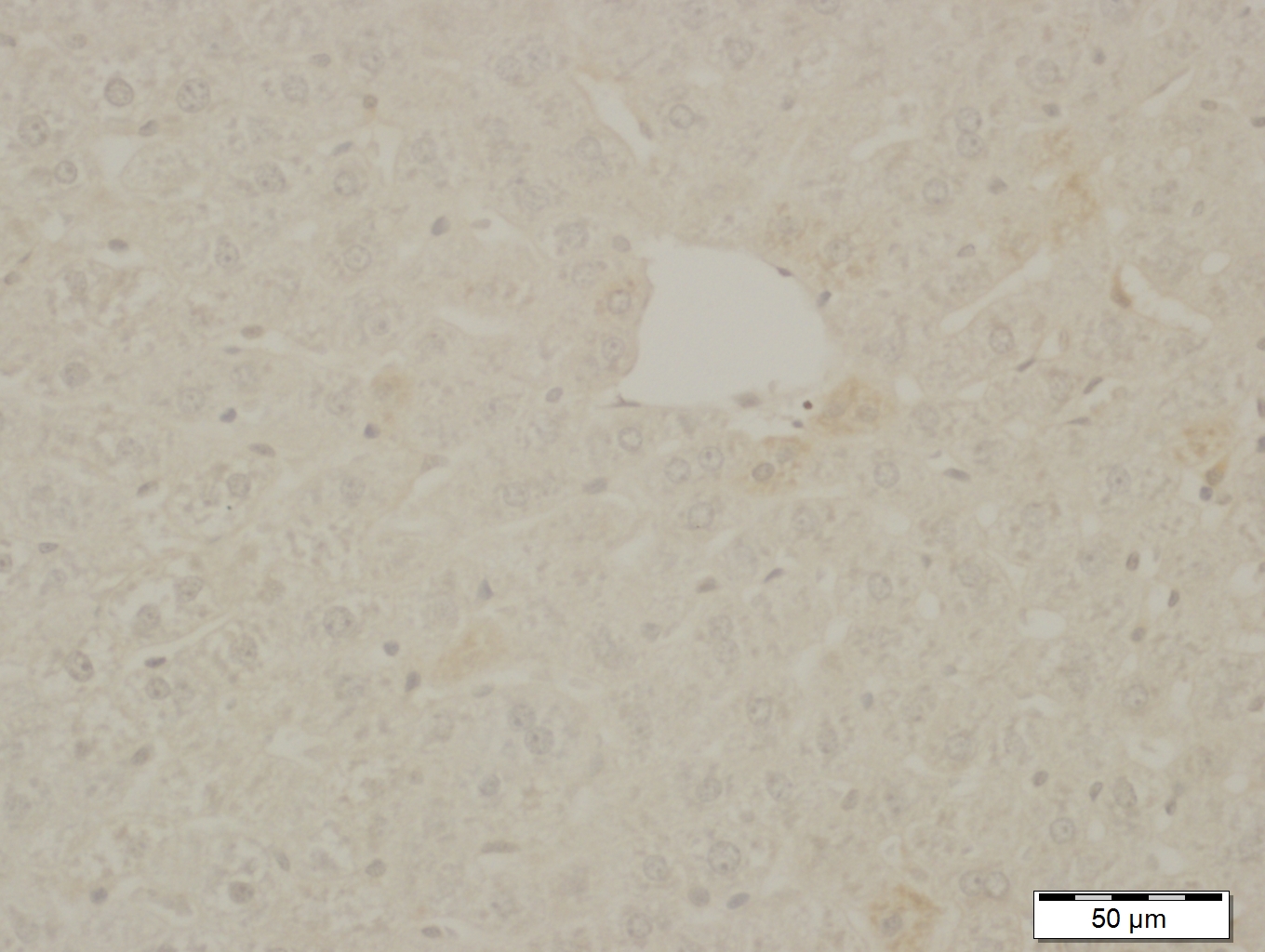

Supplement: Supplementary file 1 [file pharmaceuticals-18-00828-s001.zip › H&E and Immune images/Liver-TLR4-Sumayya/Liver-MTX+IAA-TL-X400-3 .jpg]

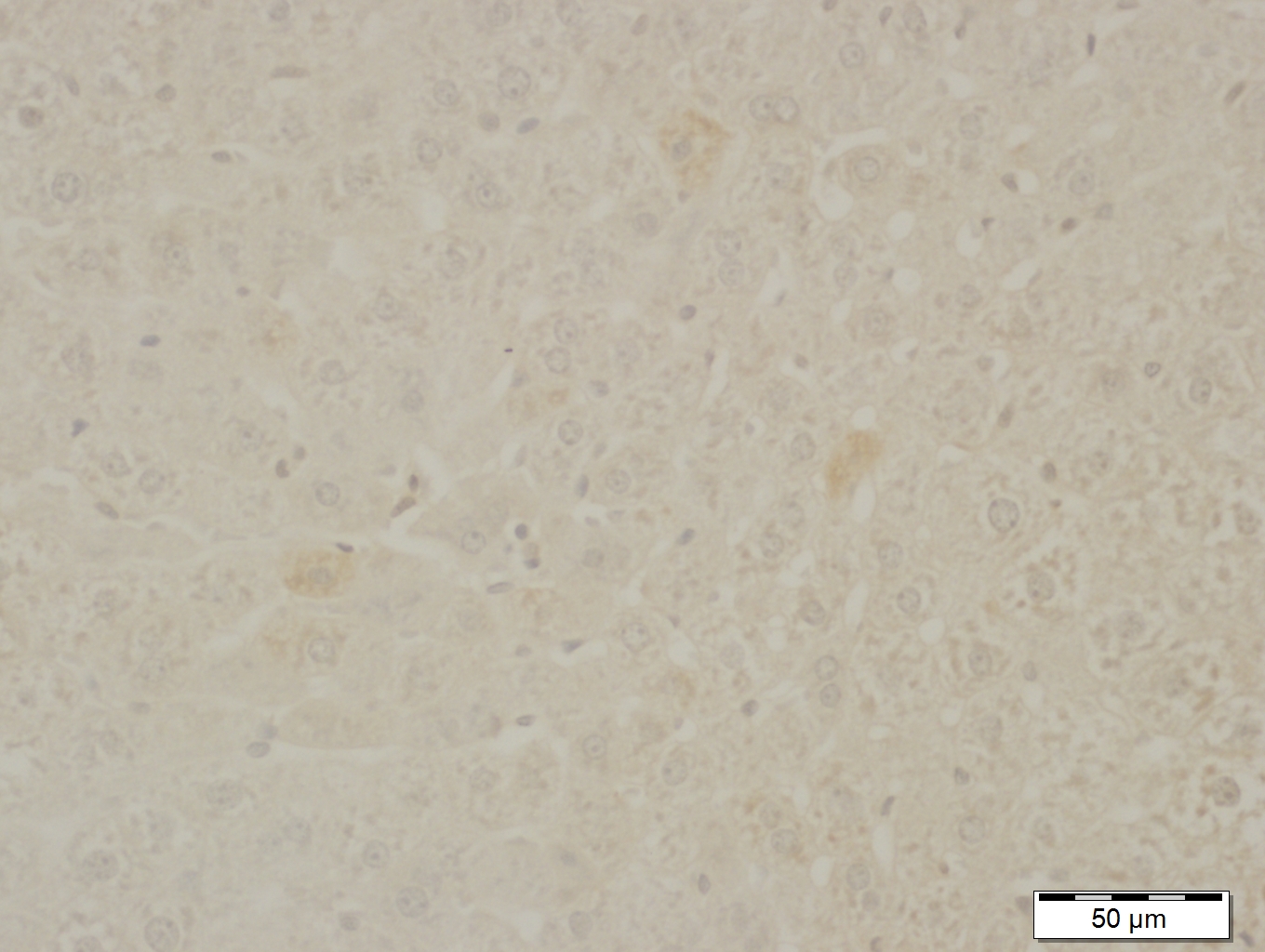

Supplement: Supplementary file 1 [file pharmaceuticals-18-00828-s001.zip › H&E and Immune images/Liver-TLR4-Sumayya/Liver-MTX+IAA-TL-X400-4 .jpg]

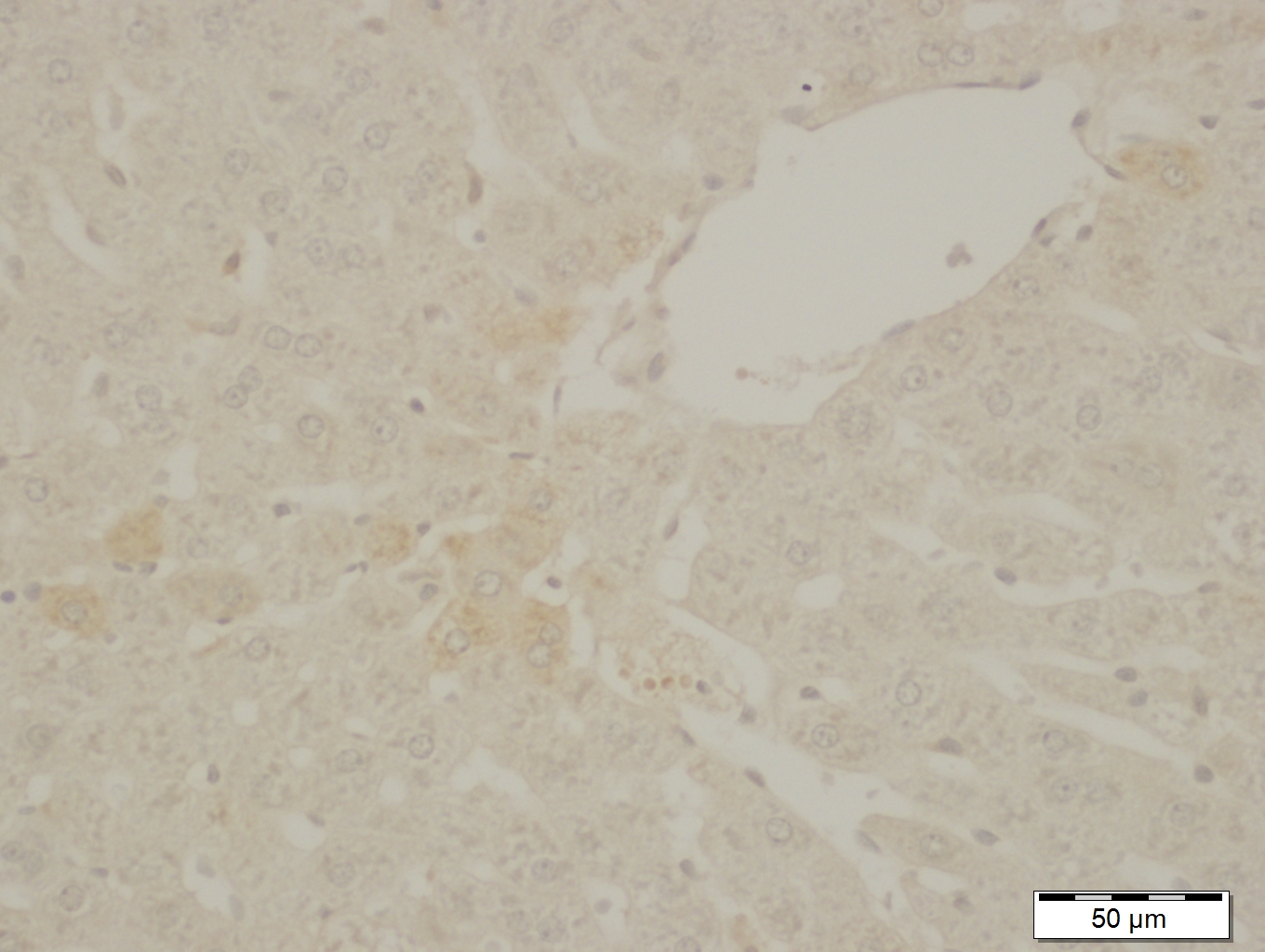

Supplement: Supplementary file 1 [file pharmaceuticals-18-00828-s001.zip › H&E and Immune images/Liver-TLR4-Sumayya/Liver-MTX+IAA-TL-X400-5 .jpg]

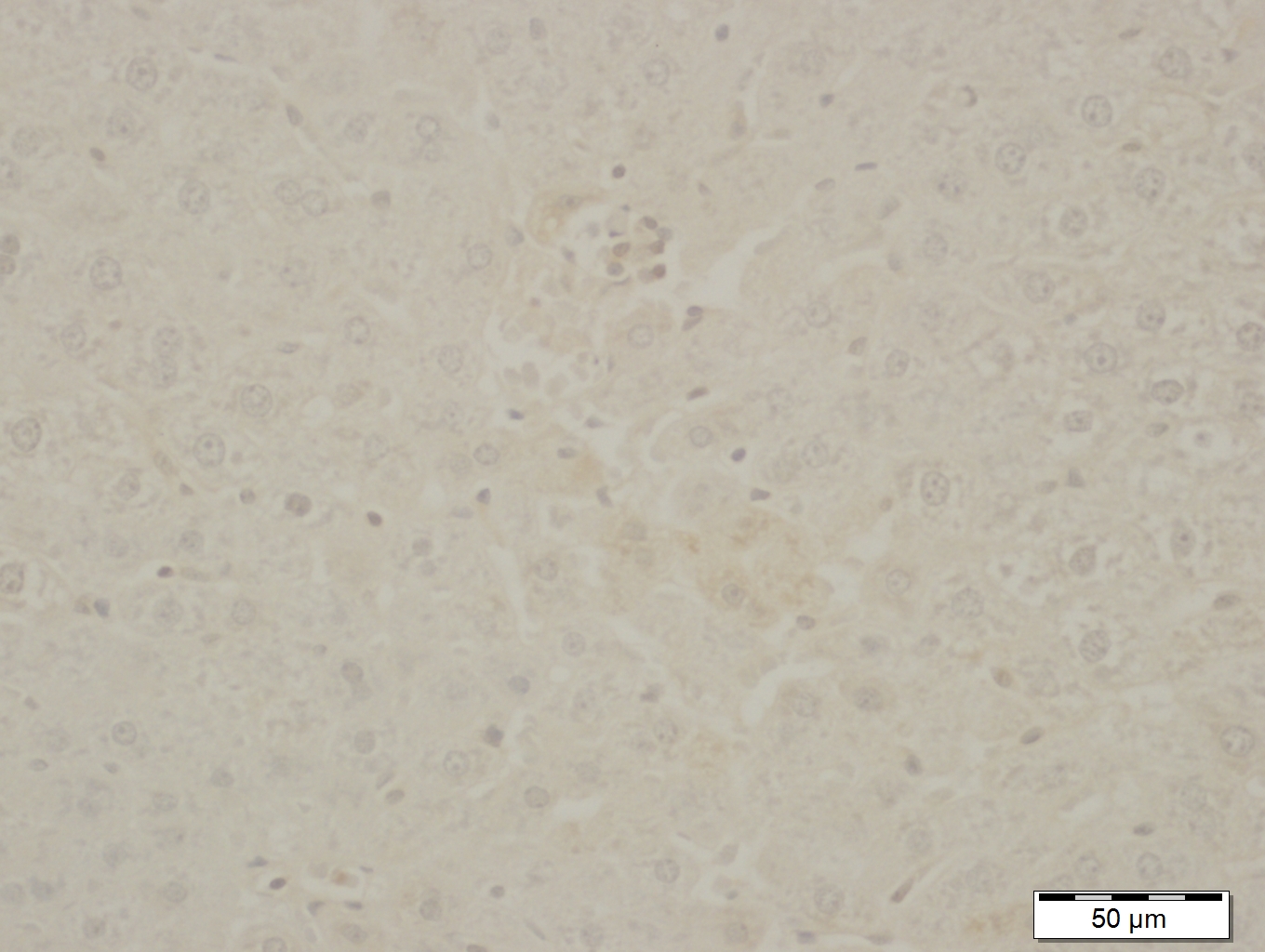

Supplement: Supplementary file 1 [file pharmaceuticals-18-00828-s001.zip › H&E and Immune images/Liver-TLR4-Sumayya/Liver-MTX+IAA-TL-X400-6 .jpg]

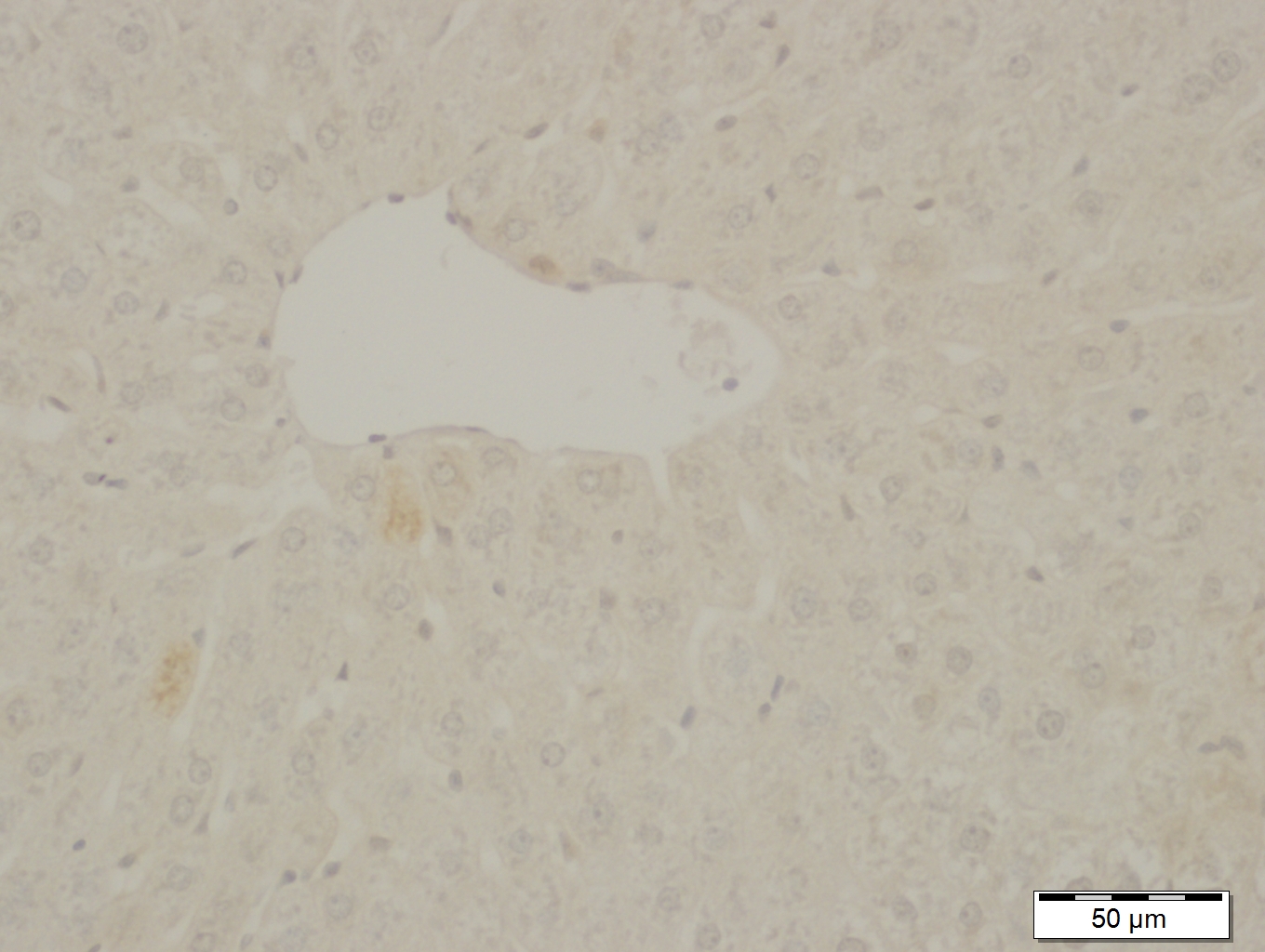

Supplement: Supplementary file 1 [file pharmaceuticals-18-00828-s001.zip › H&E and Immune images/Liver-TLR4-Sumayya/Liver-MTX+IAA-TL-X400-7 .jpg]

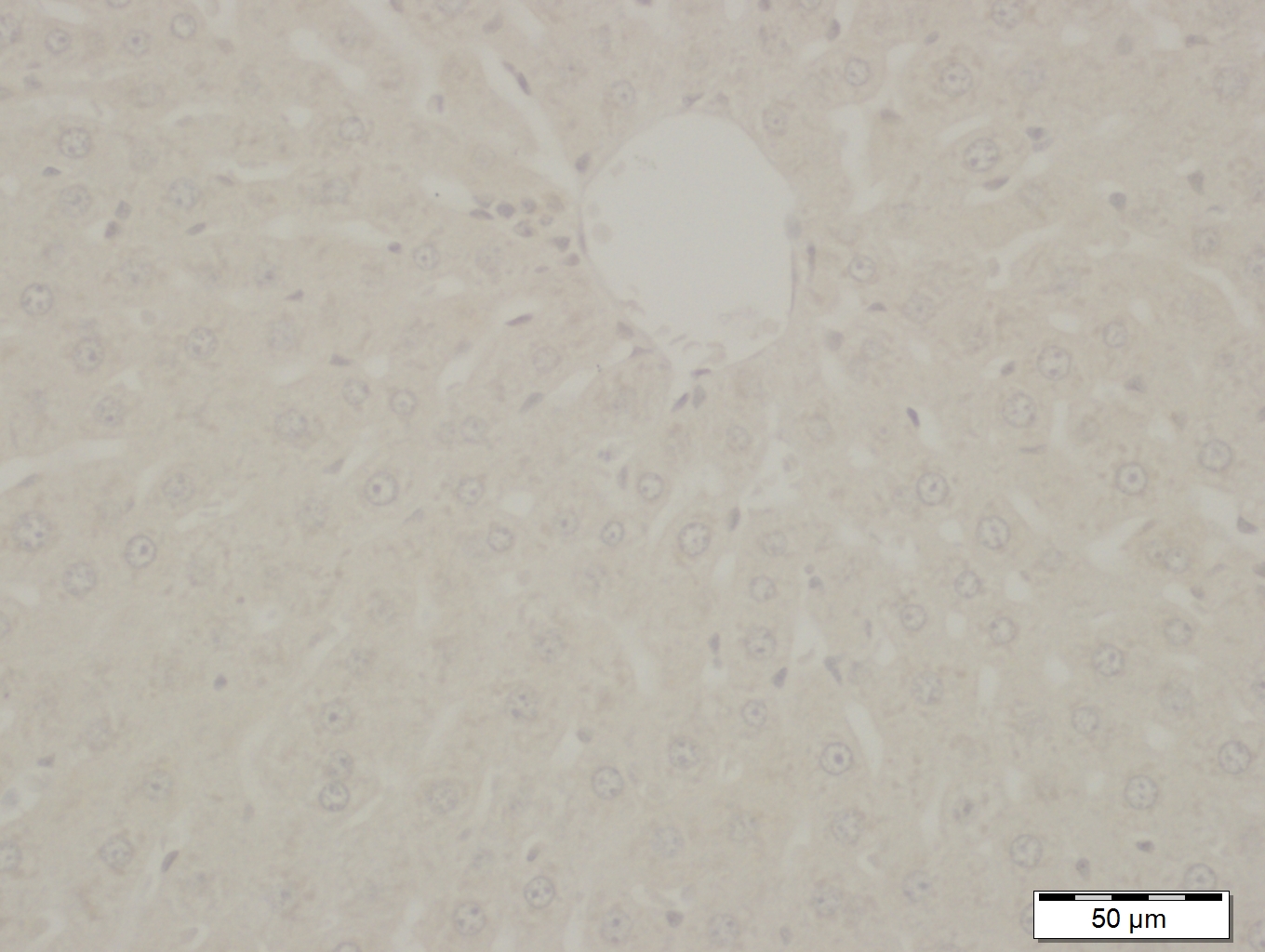

Supplement: Supplementary file 1 [file pharmaceuticals-18-00828-s001.zip › H&E and Immune images/Liver-TLR4-Sumayya/Liver-MTX+QUR-TL-X400-1 .jpg]

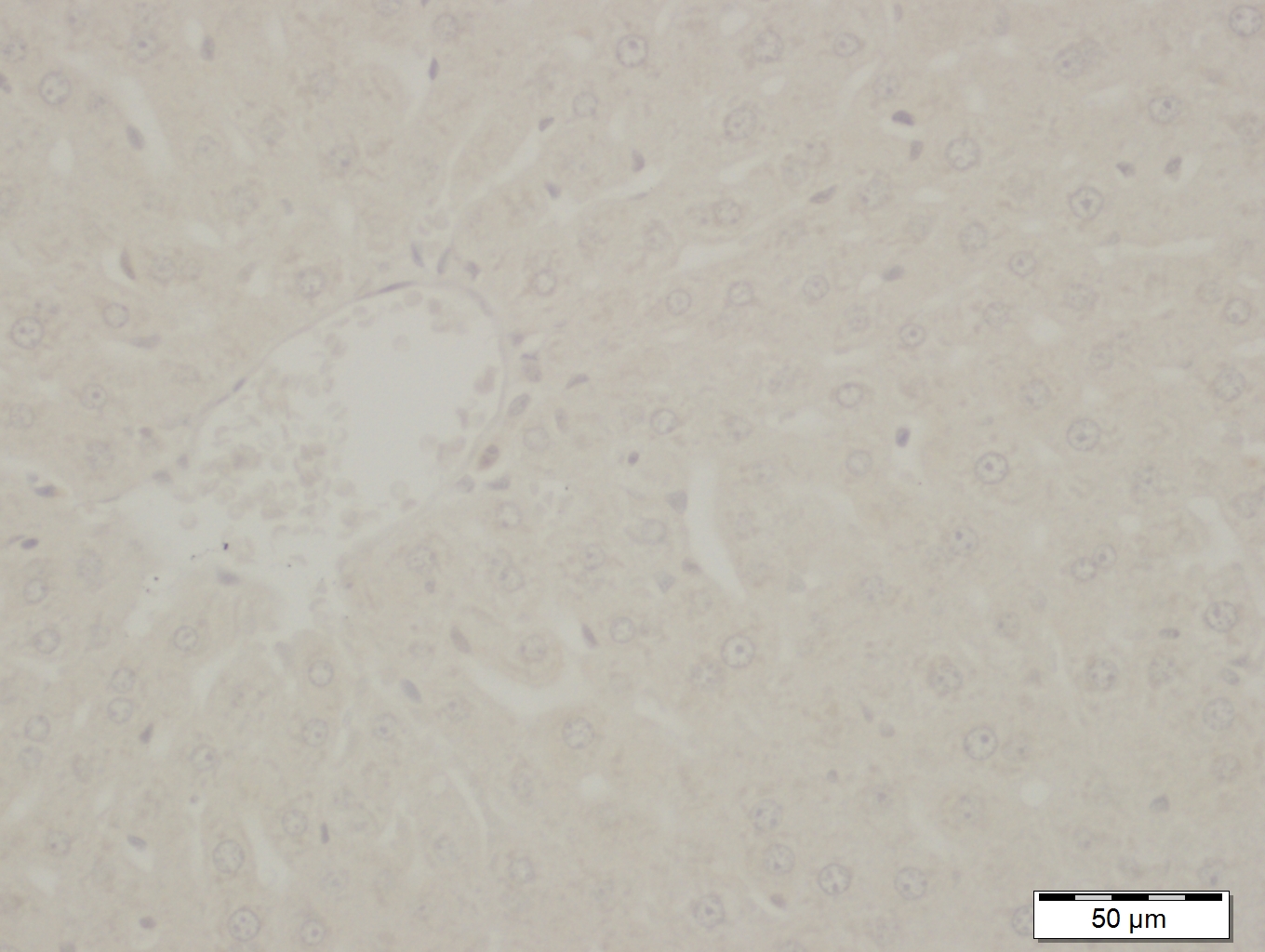

Supplement: Supplementary file 1 [file pharmaceuticals-18-00828-s001.zip › H&E and Immune images/Liver-TLR4-Sumayya/Liver-MTX+QUR-TL-X400-2 .jpg]

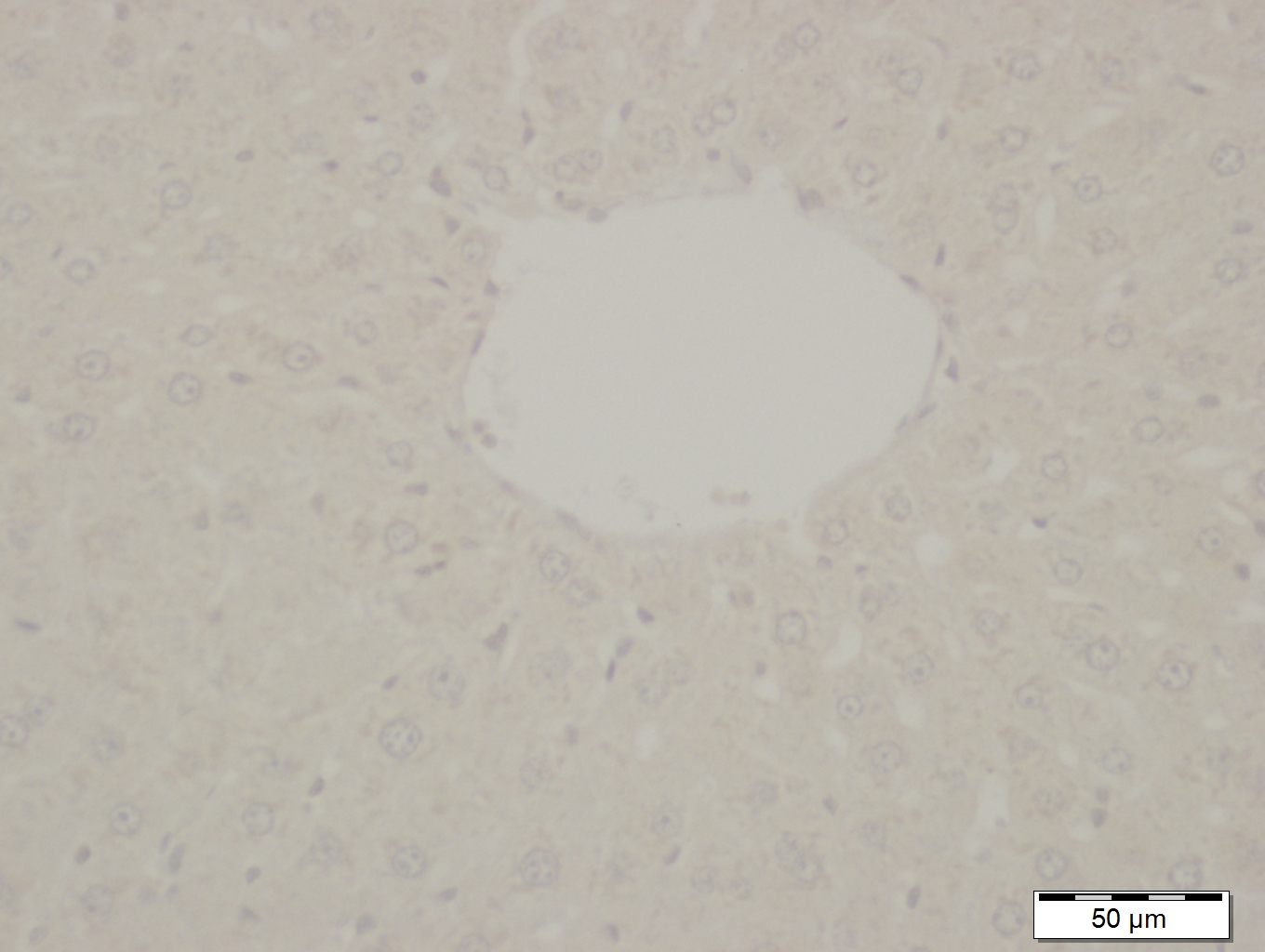

Supplement: Supplementary file 1 [file pharmaceuticals-18-00828-s001.zip › H&E and Immune images/Liver-TLR4-Sumayya/Liver-MTX+QUR-TL-X400-3 .jpg]

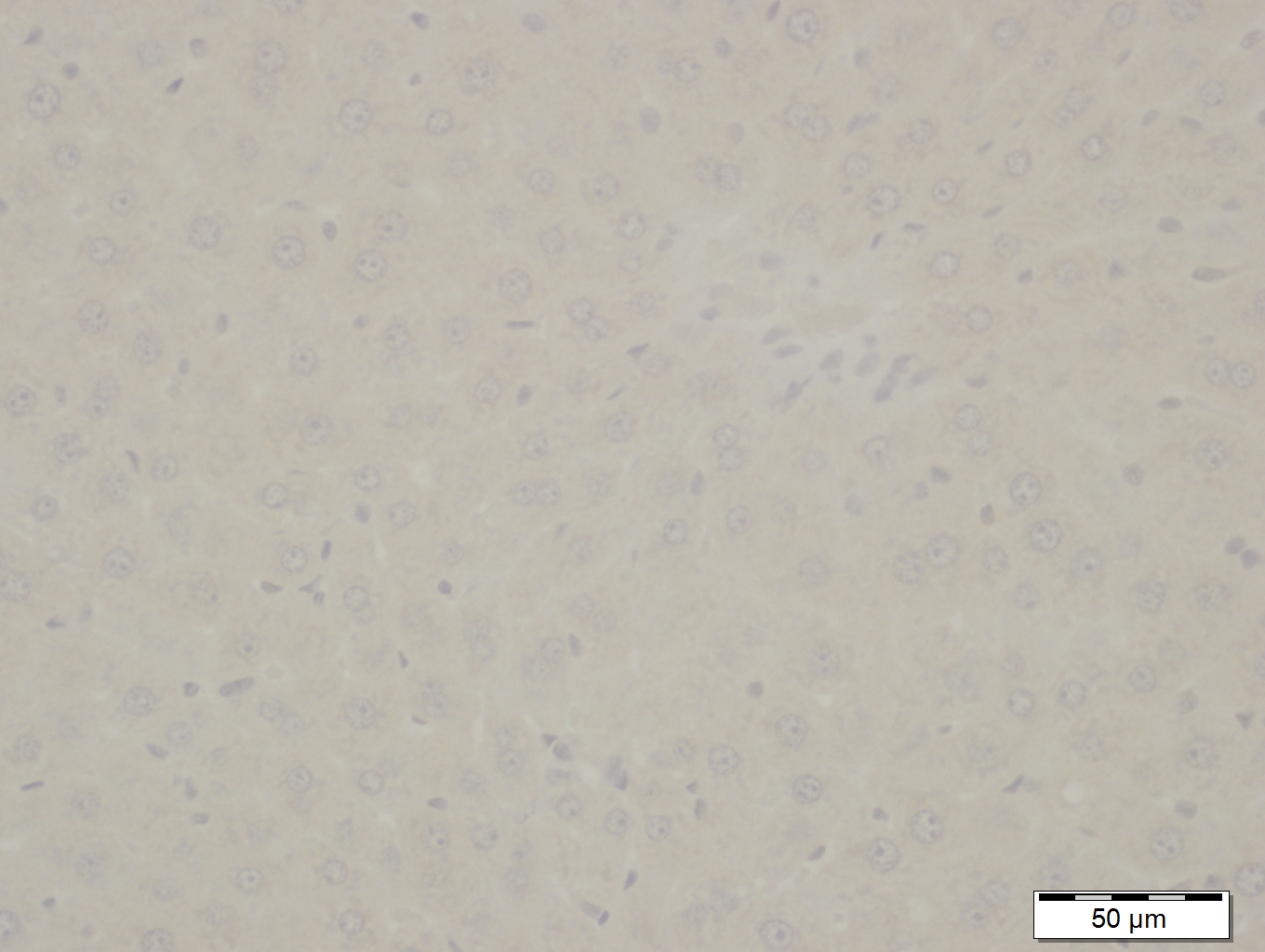

Supplement: Supplementary file 1 [file pharmaceuticals-18-00828-s001.zip › H&E and Immune images/Liver-TLR4-Sumayya/Liver-MTX+QUR-TL-X400-5 .jpg]

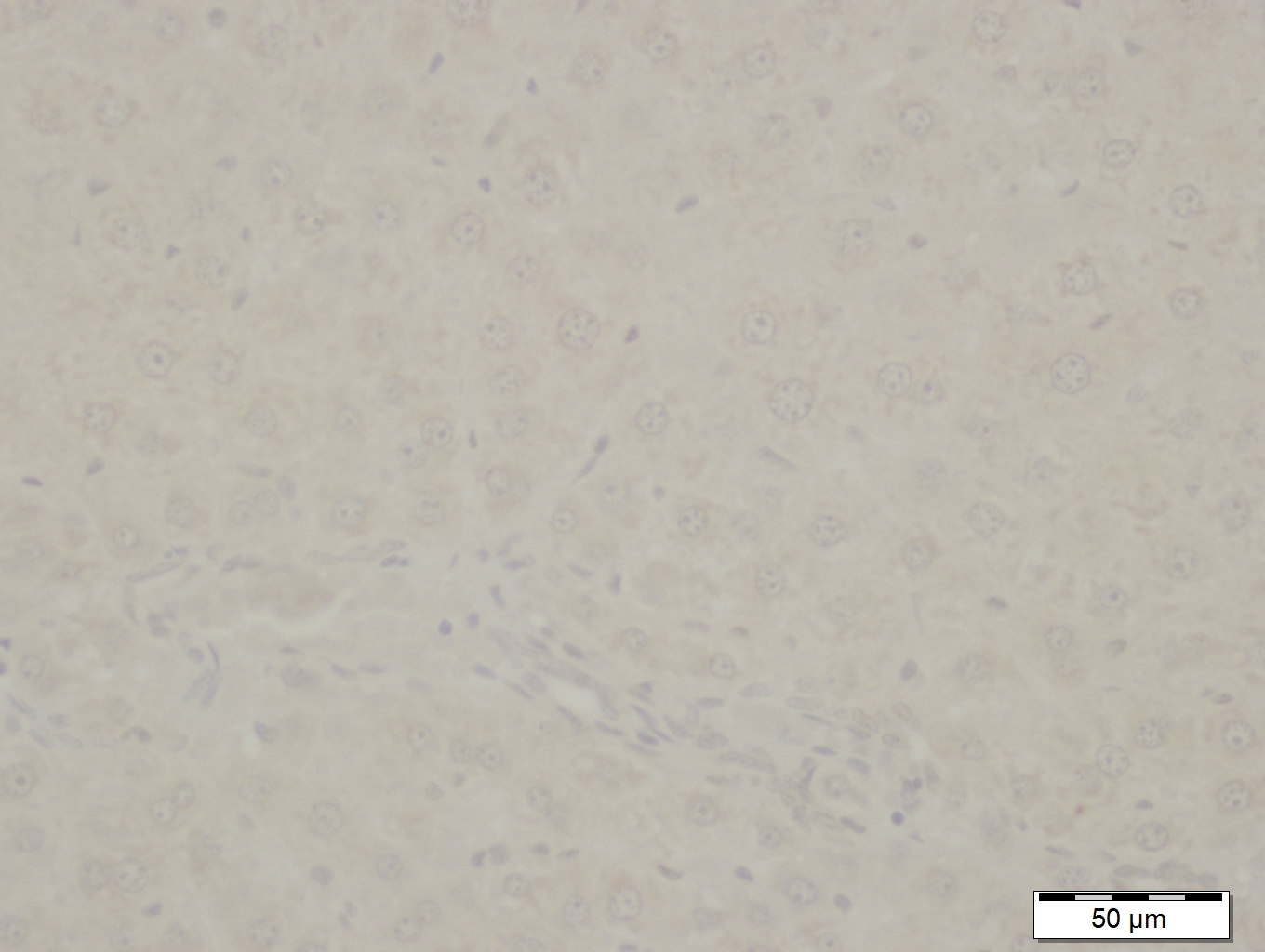

Supplement: Supplementary file 1 [file pharmaceuticals-18-00828-s001.zip › H&E and Immune images/Liver-TLR4-Sumayya/Liver-MTX+QUR-TL-X400-6 .jpg]

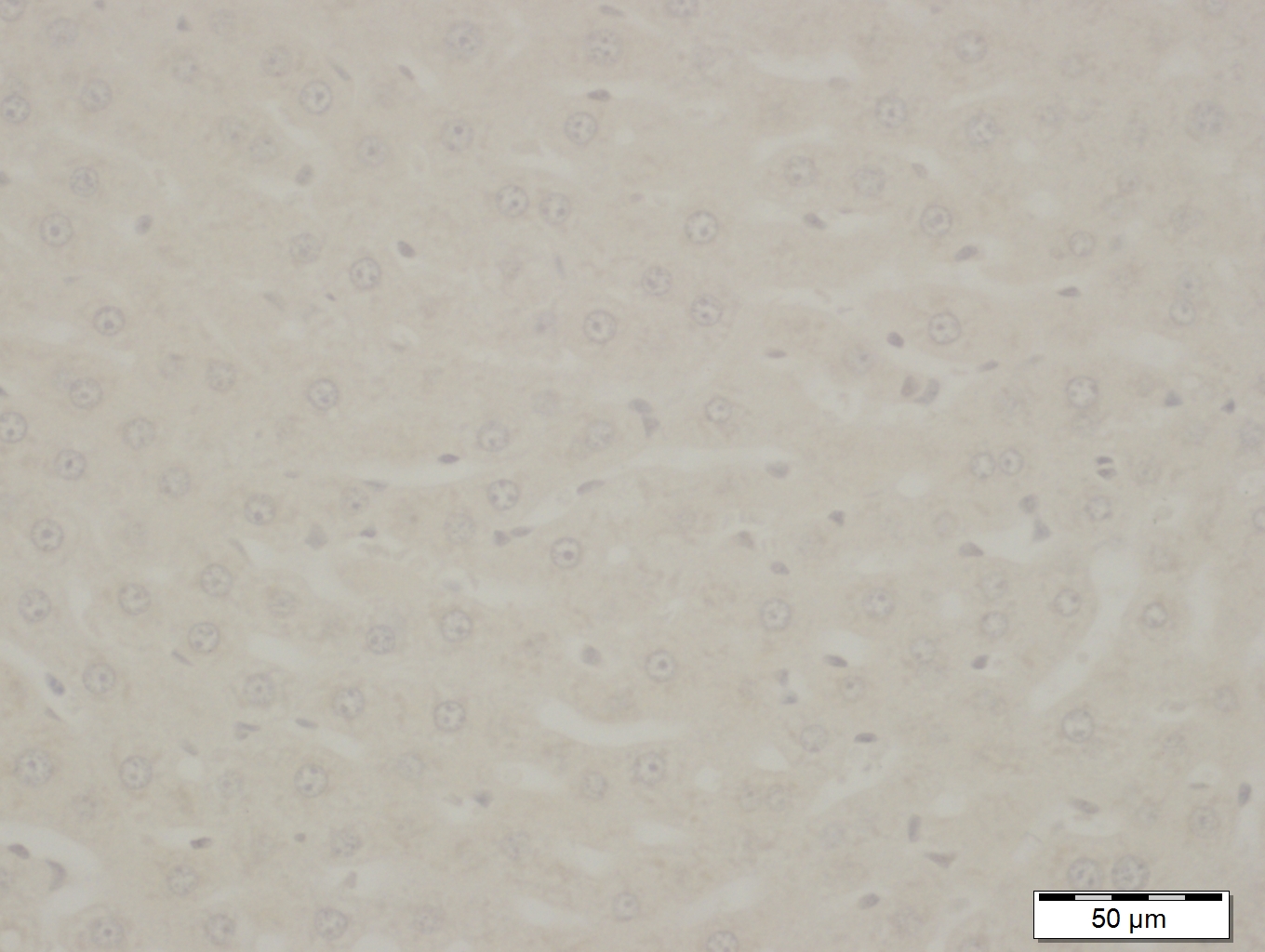

Supplement: Supplementary file 1 [file pharmaceuticals-18-00828-s001.zip › H&E and Immune images/Liver-TLR4-Sumayya/Liver-MTX+QUR-TL-X400-8 .jpg]

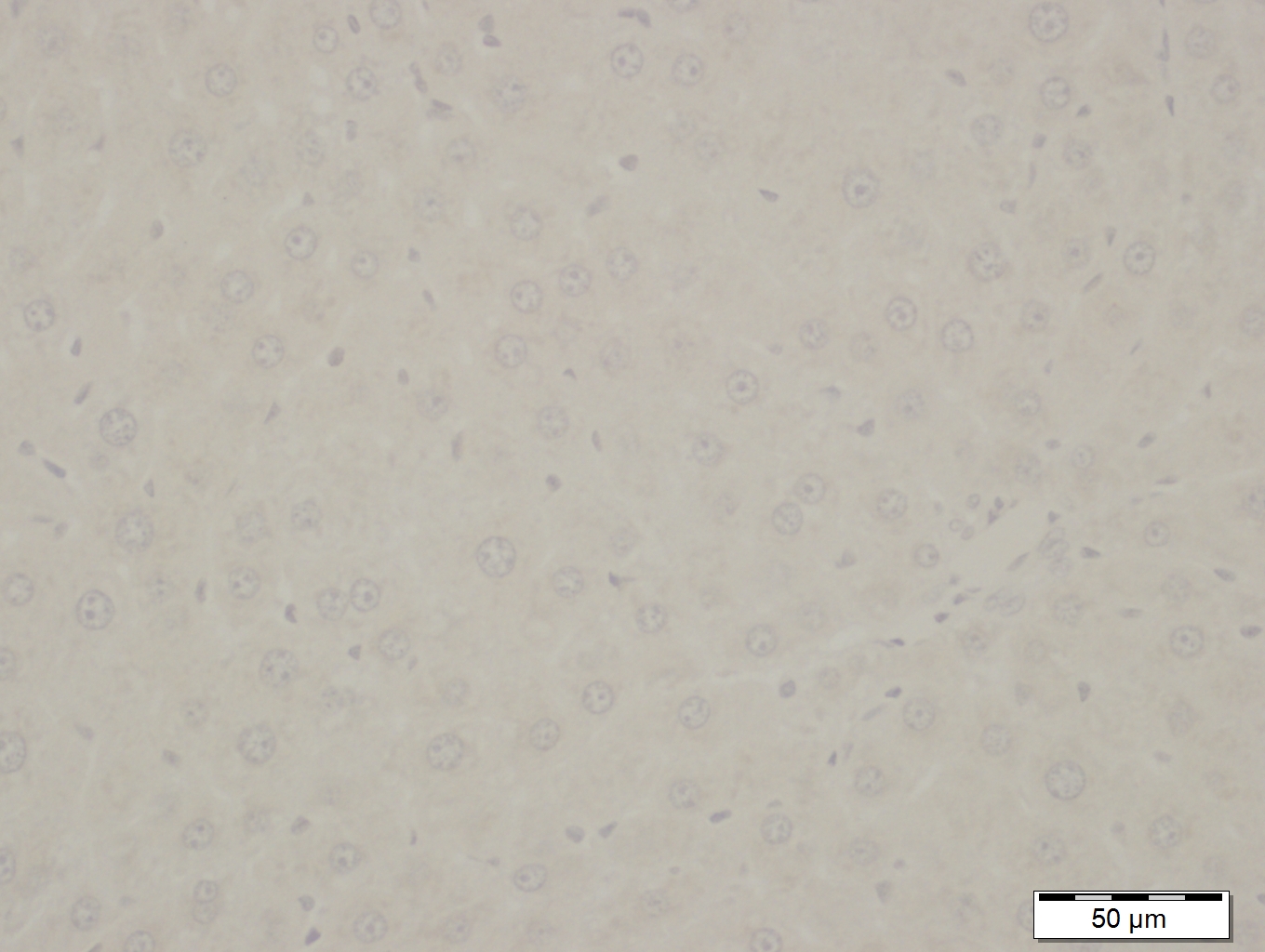

Supplement: Supplementary file 1 [file pharmaceuticals-18-00828-s001.zip › H&E and Immune images/Liver-TLR4-Sumayya/Liver-MTX+QUR-TL-X400-9 .jpg]

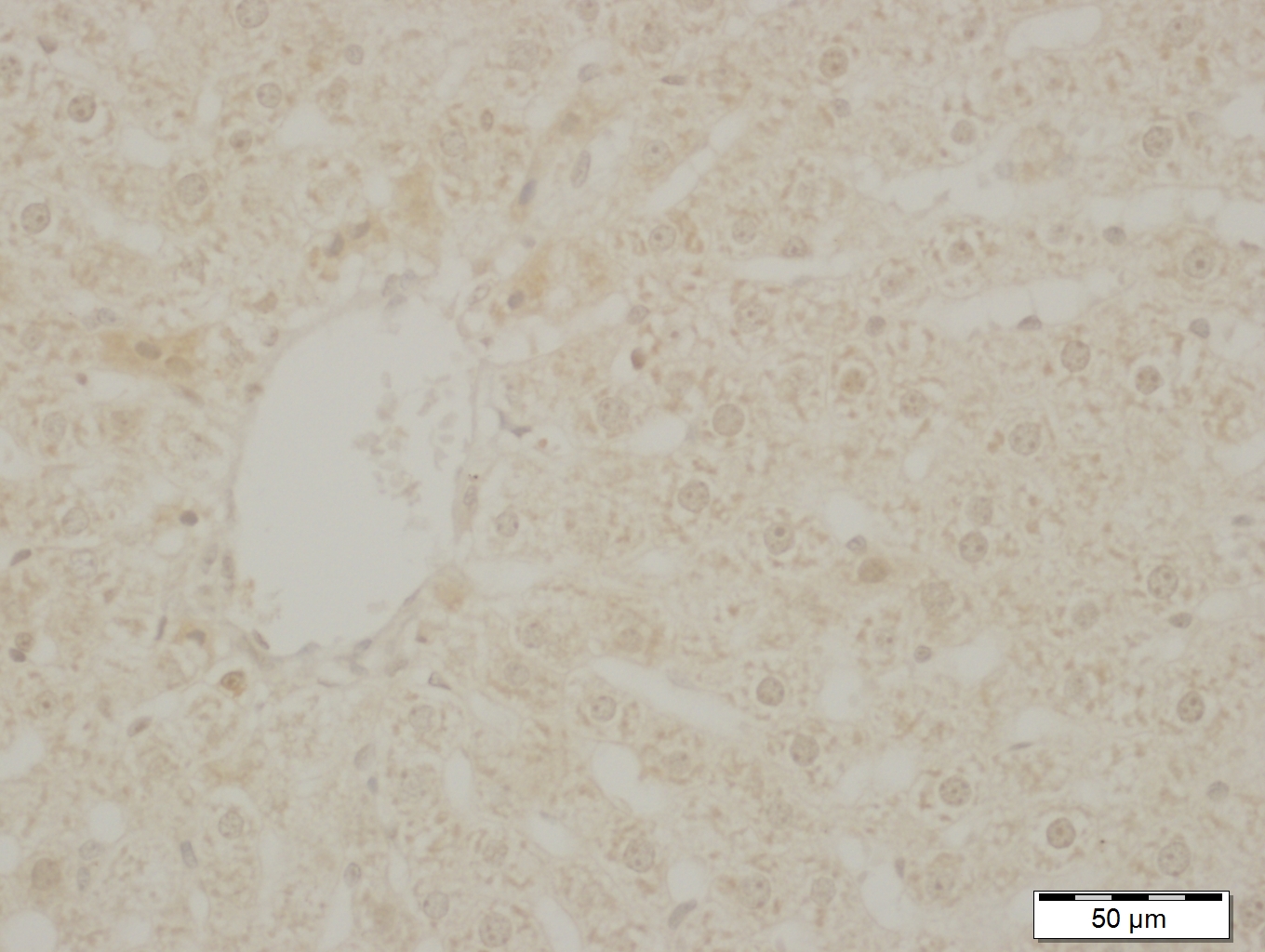

Supplement: Supplementary file 1 [file pharmaceuticals-18-00828-s001.zip › H&E and Immune images/Liver-TLR4-Sumayya/Liver-MTX-TL-X400-1 .jpg]

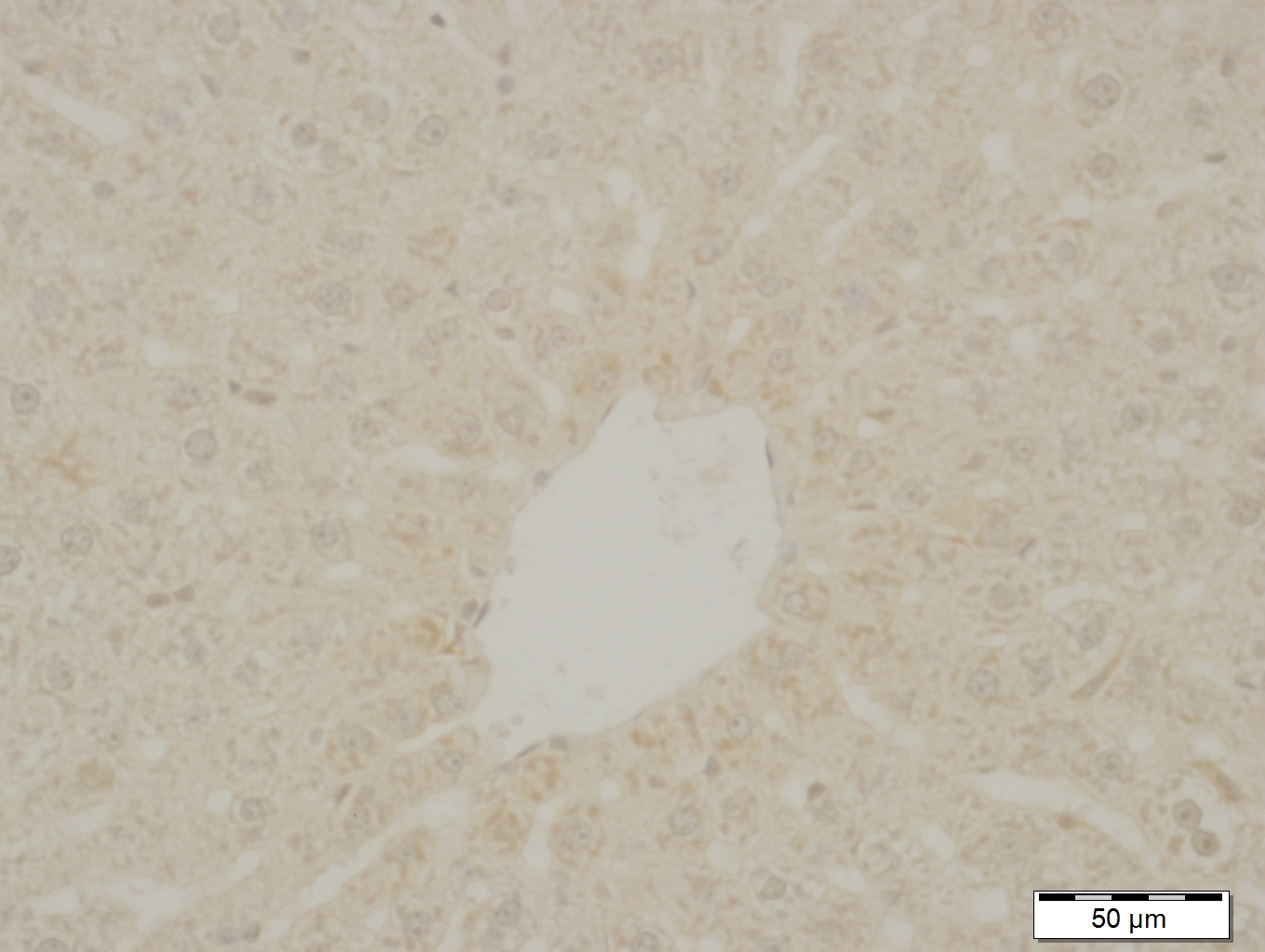

Supplement: Supplementary file 1 [file pharmaceuticals-18-00828-s001.zip › H&E and Immune images/Liver-TLR4-Sumayya/Liver-MTX-TL-X400-2 .jpg]

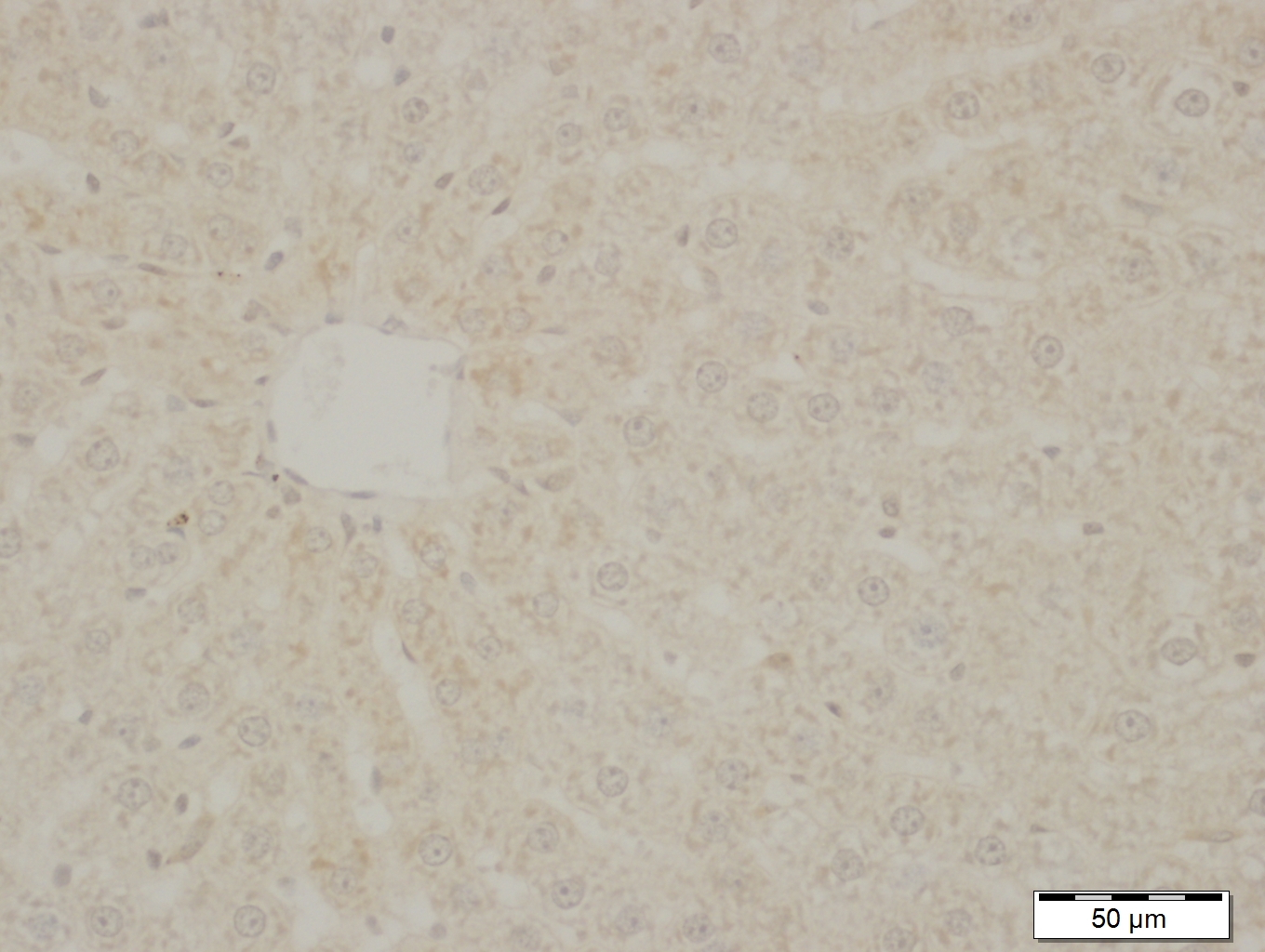

Supplement: Supplementary file 1 [file pharmaceuticals-18-00828-s001.zip › H&E and Immune images/Liver-TLR4-Sumayya/Liver-MTX-TL-X400-3 .jpg]

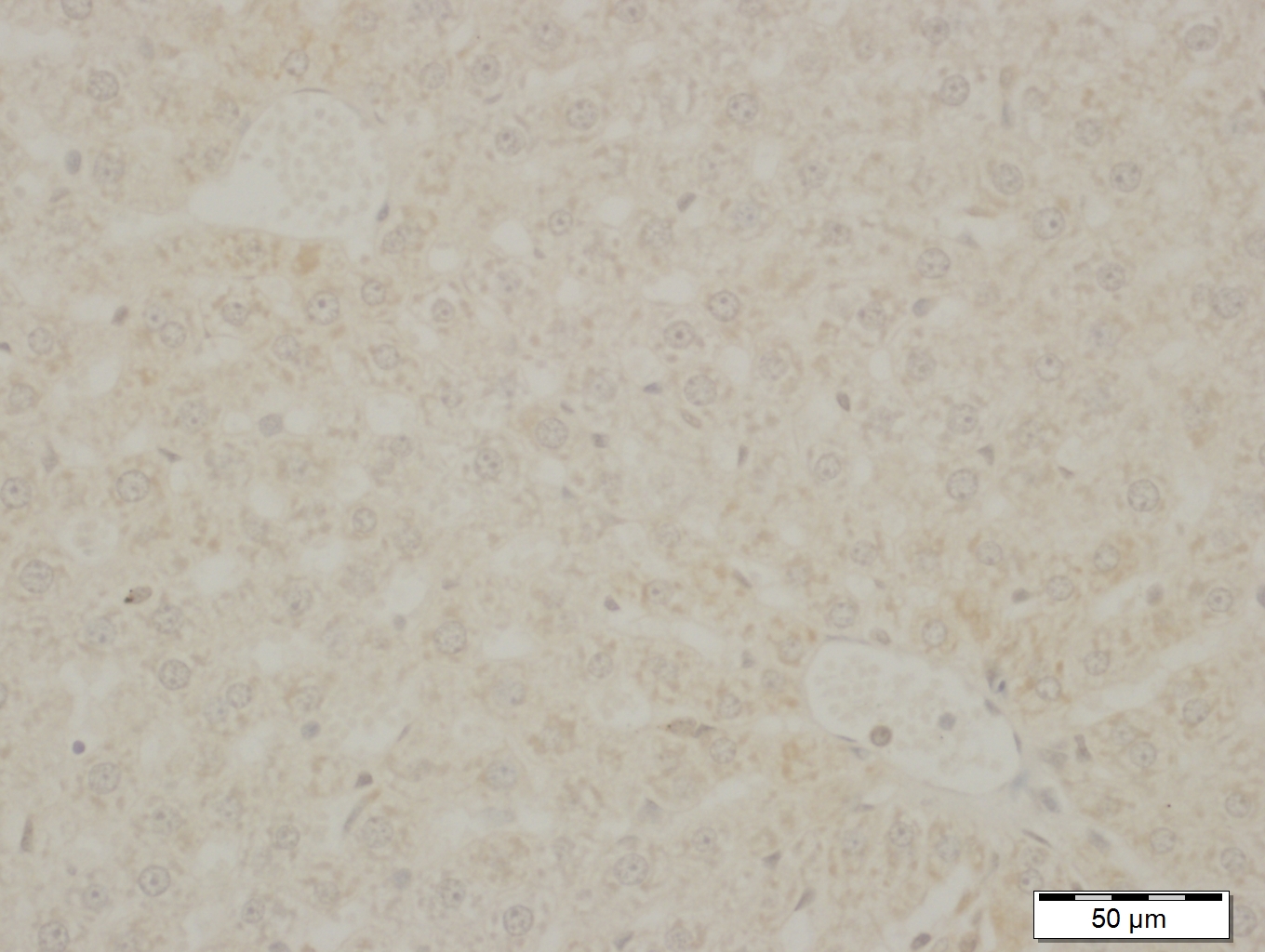

Supplement: Supplementary file 1 [file pharmaceuticals-18-00828-s001.zip › H&E and Immune images/Liver-TLR4-Sumayya/Liver-MTX-TL-X400-4 .jpg]

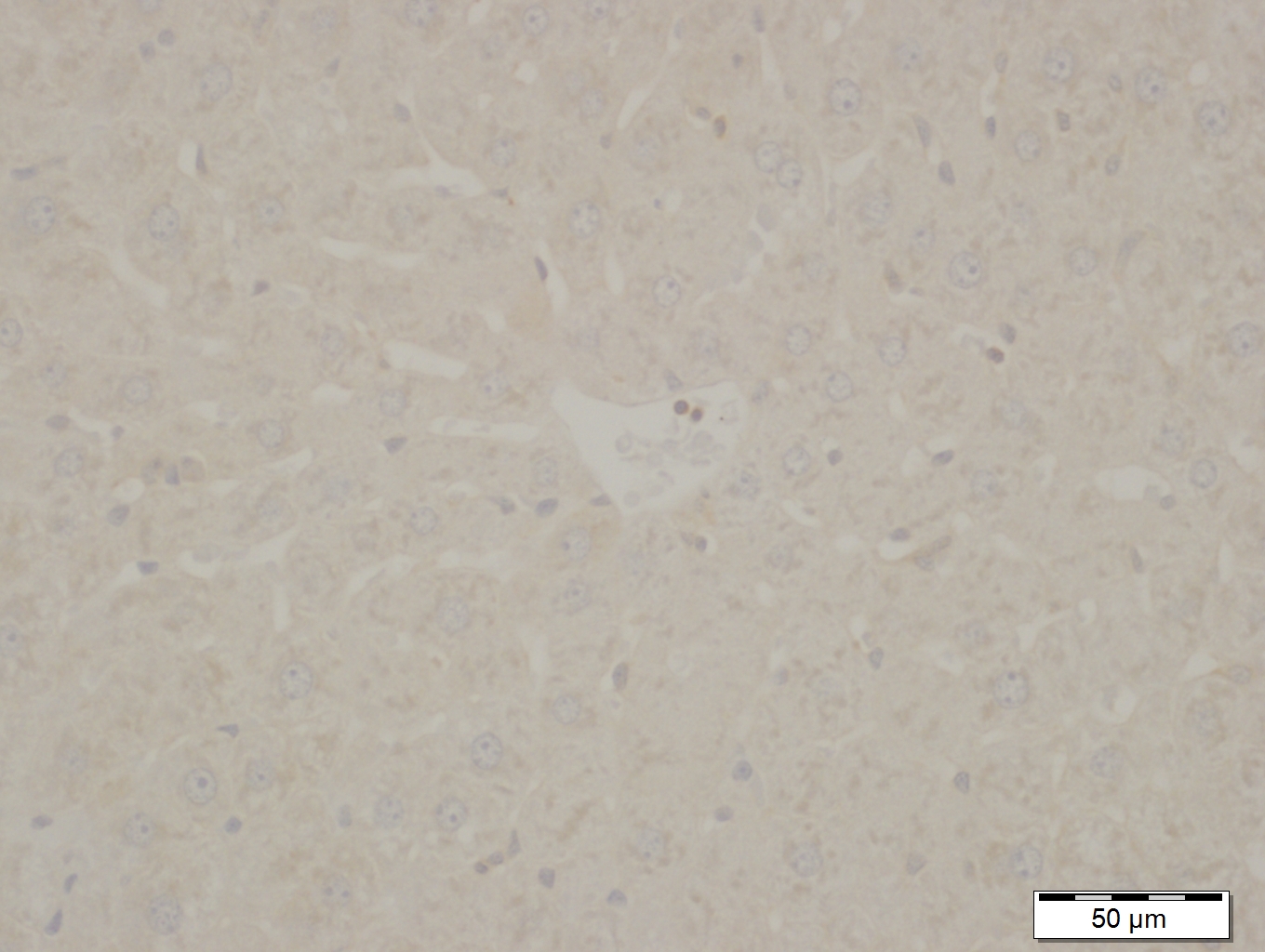

Supplement: Supplementary file 1 [file pharmaceuticals-18-00828-s001.zip › H&E and Immune images/Liver-TNF-KB-Sumayya/Liver-Cont-TNFKB-X400-1 .jpg]

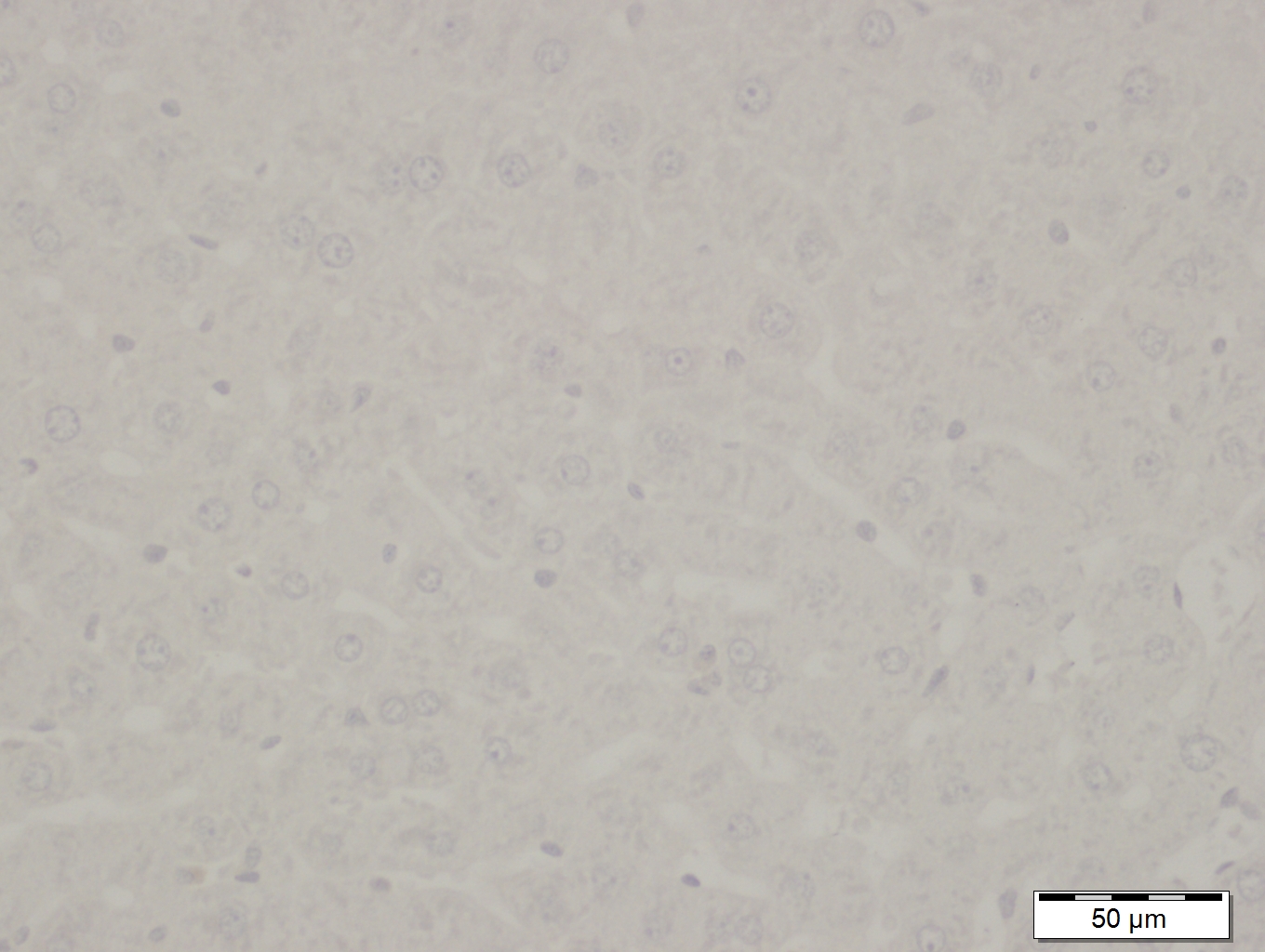

Supplement: Supplementary file 1 [file pharmaceuticals-18-00828-s001.zip › H&E and Immune images/Liver-TNF-KB-Sumayya/Liver-Cont-TNFKB-X400-10 .jpg]

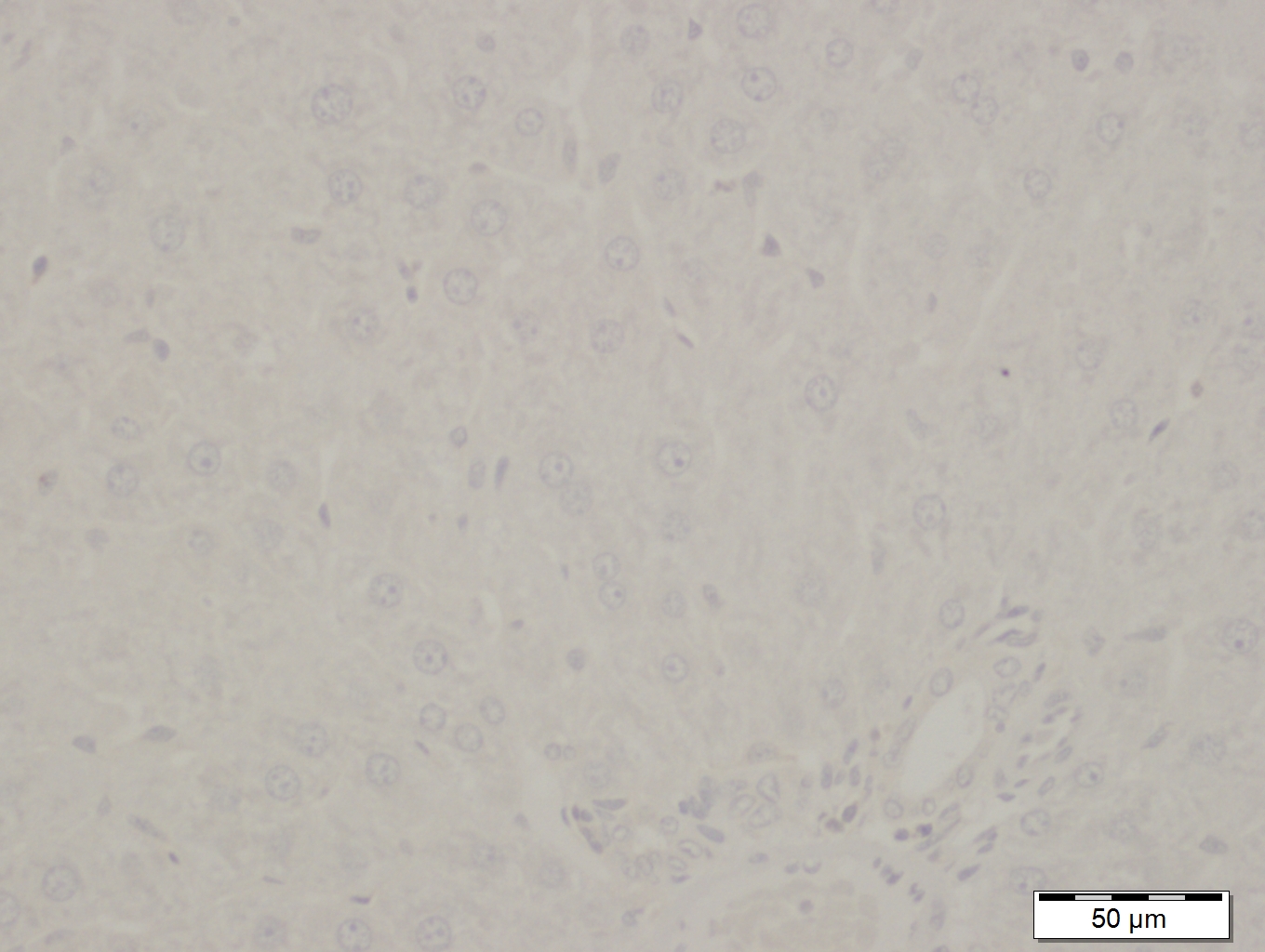

Supplement: Supplementary file 1 [file pharmaceuticals-18-00828-s001.zip › H&E and Immune images/Liver-TNF-KB-Sumayya/Liver-Cont-TNFKB-X400-11 .jpg]

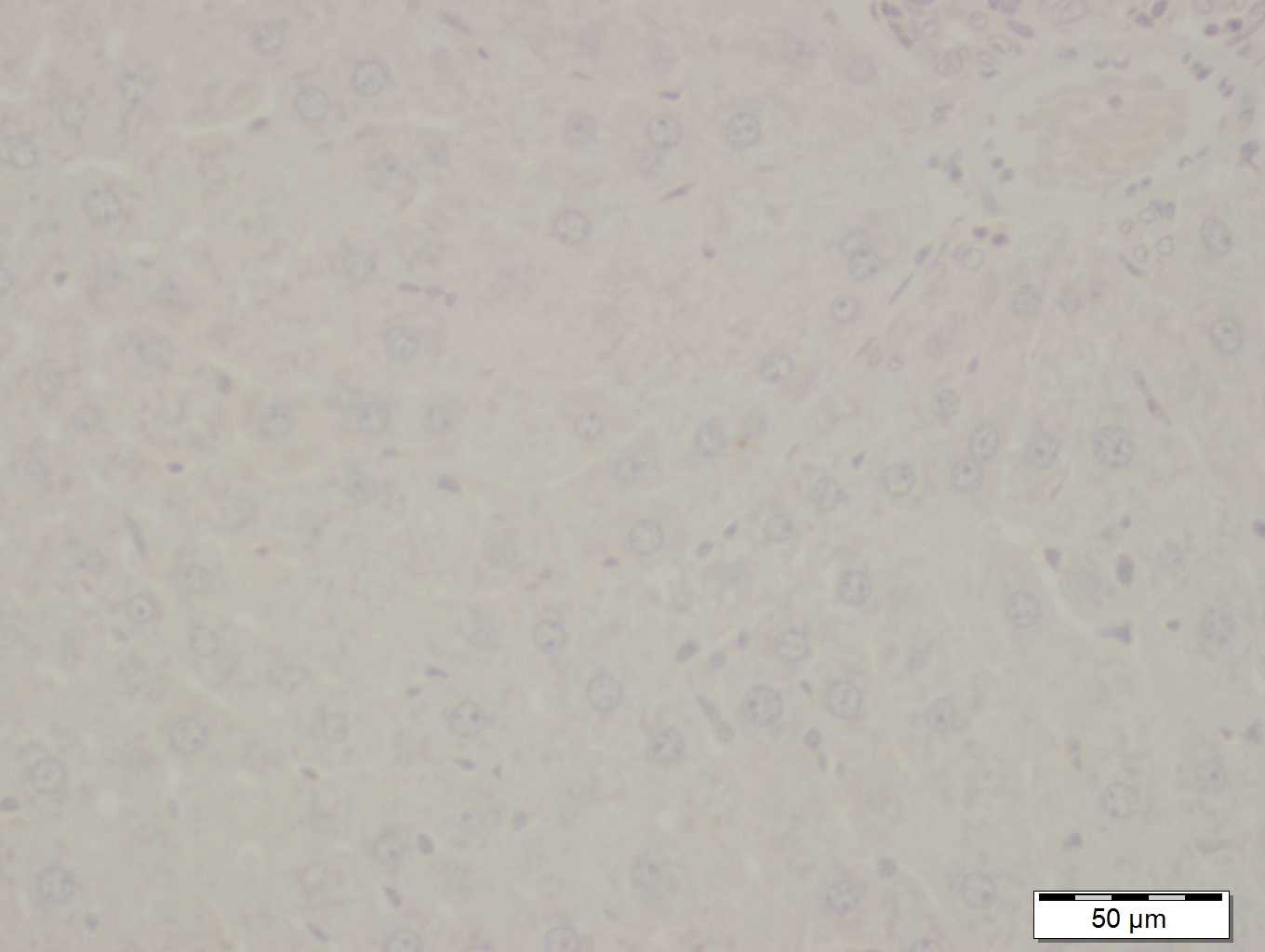

Supplement: Supplementary file 1 [file pharmaceuticals-18-00828-s001.zip › H&E and Immune images/Liver-TNF-KB-Sumayya/Liver-Cont-TNFKB-X400-12 .jpg]

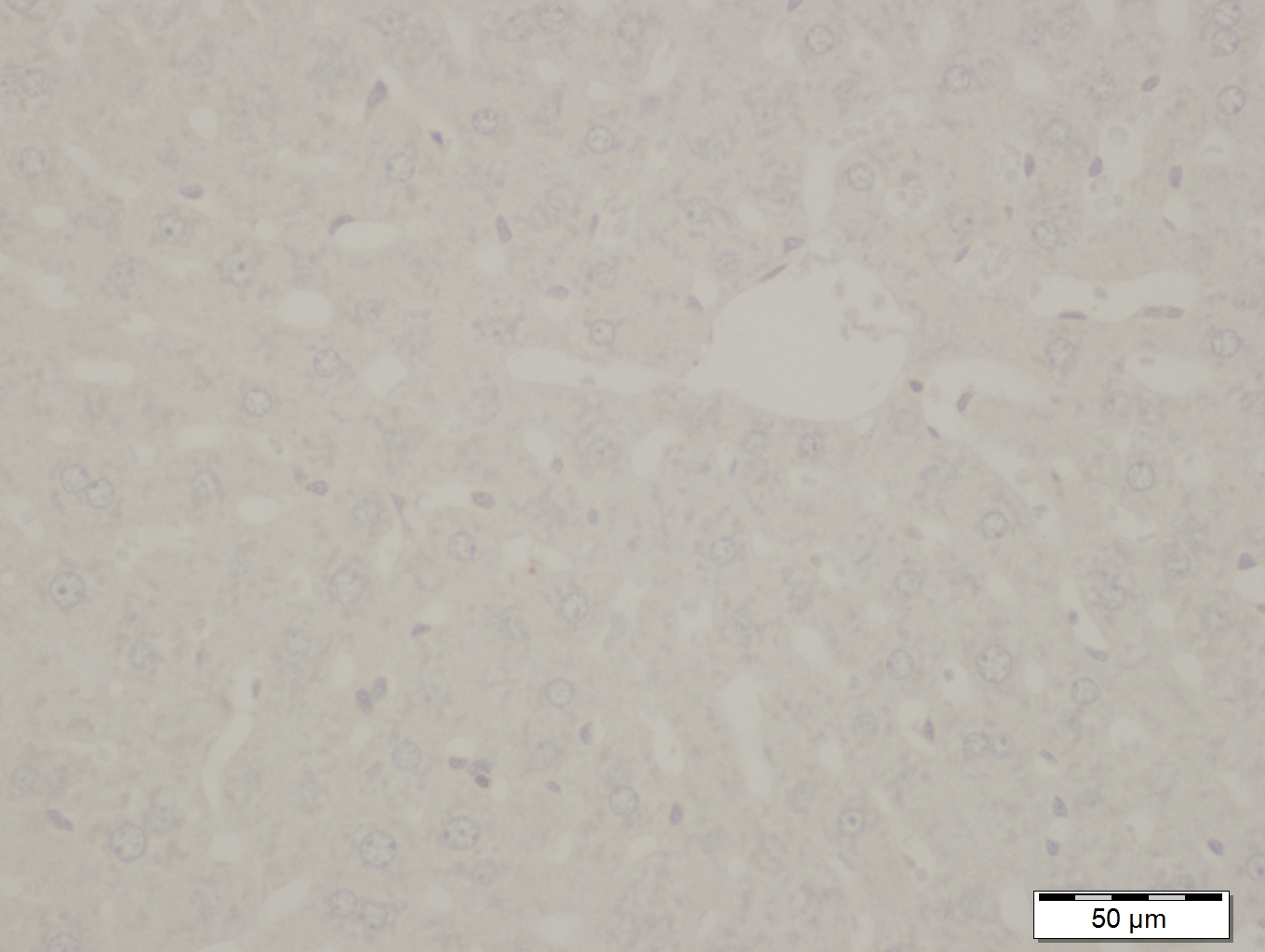

Supplement: Supplementary file 1 [file pharmaceuticals-18-00828-s001.zip › H&E and Immune images/Liver-TNF-KB-Sumayya/Liver-Cont-TNFKB-X400-15 .jpg]

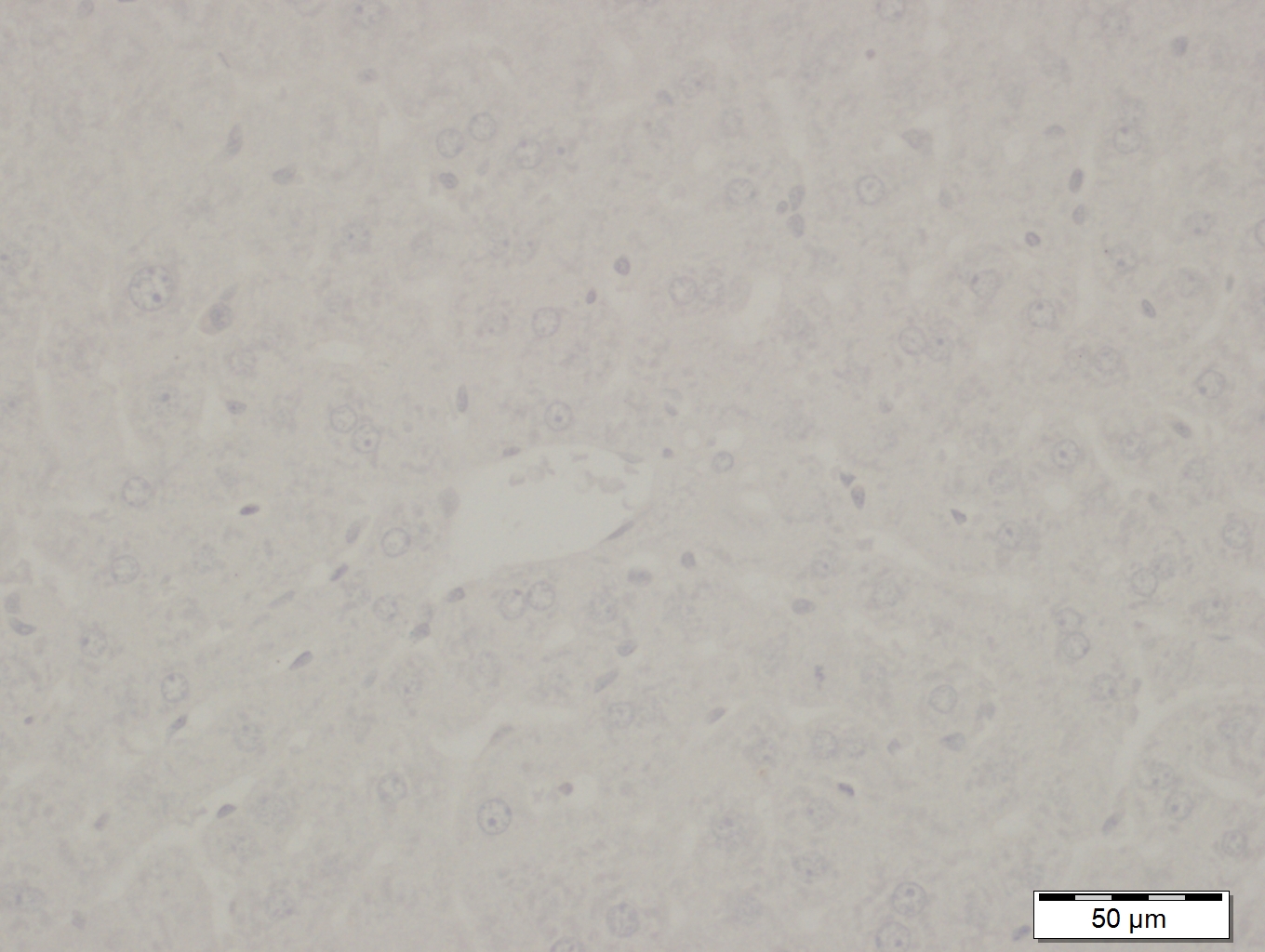

Supplement: Supplementary file 1 [file pharmaceuticals-18-00828-s001.zip › H&E and Immune images/Liver-TNF-KB-Sumayya/Liver-Cont-TNFKB-X400-18 .jpg]

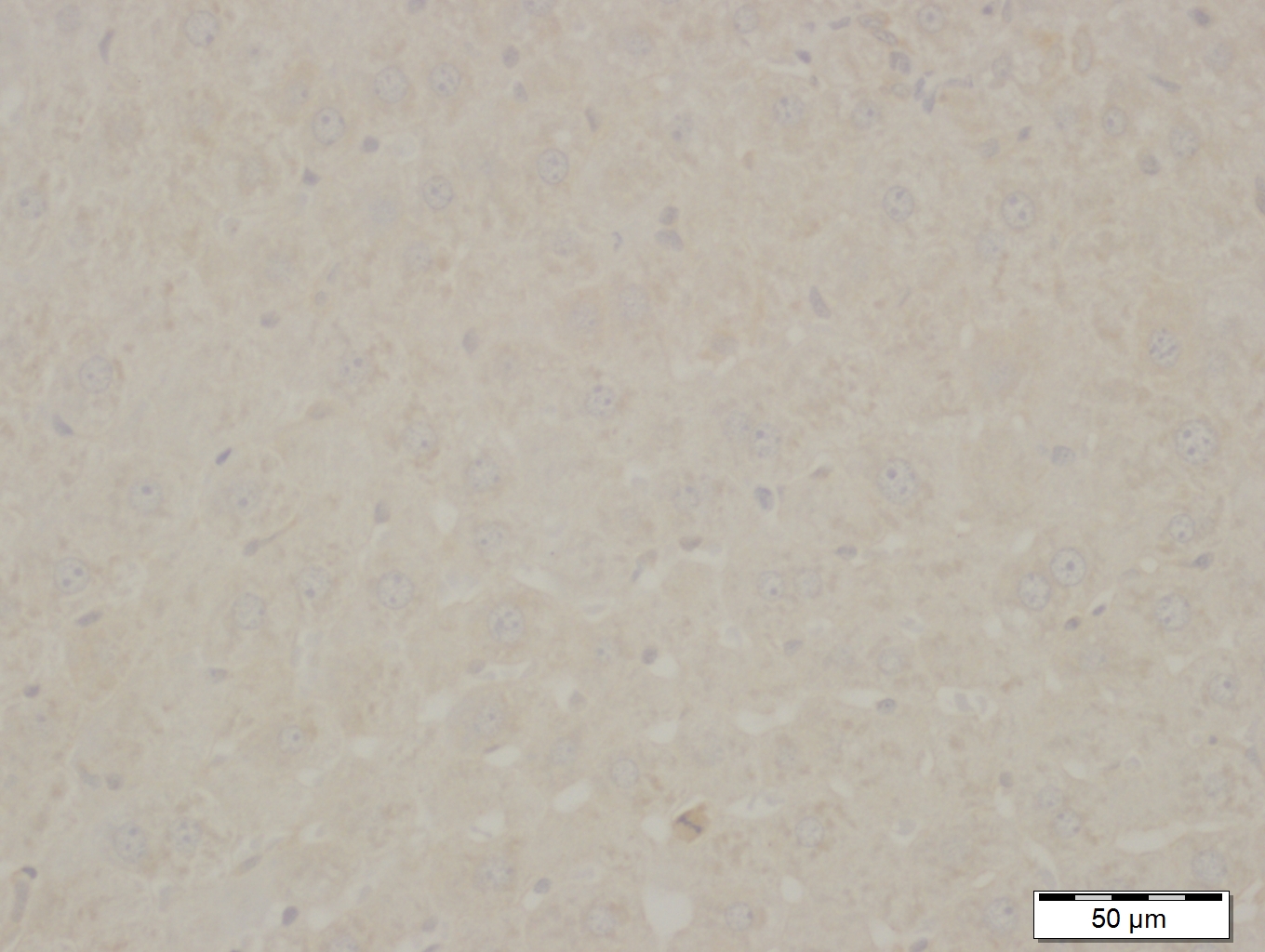

Supplement: Supplementary file 1 [file pharmaceuticals-18-00828-s001.zip › H&E and Immune images/Liver-TNF-KB-Sumayya/Liver-Cont-TNFKB-X400-2 .jpg]

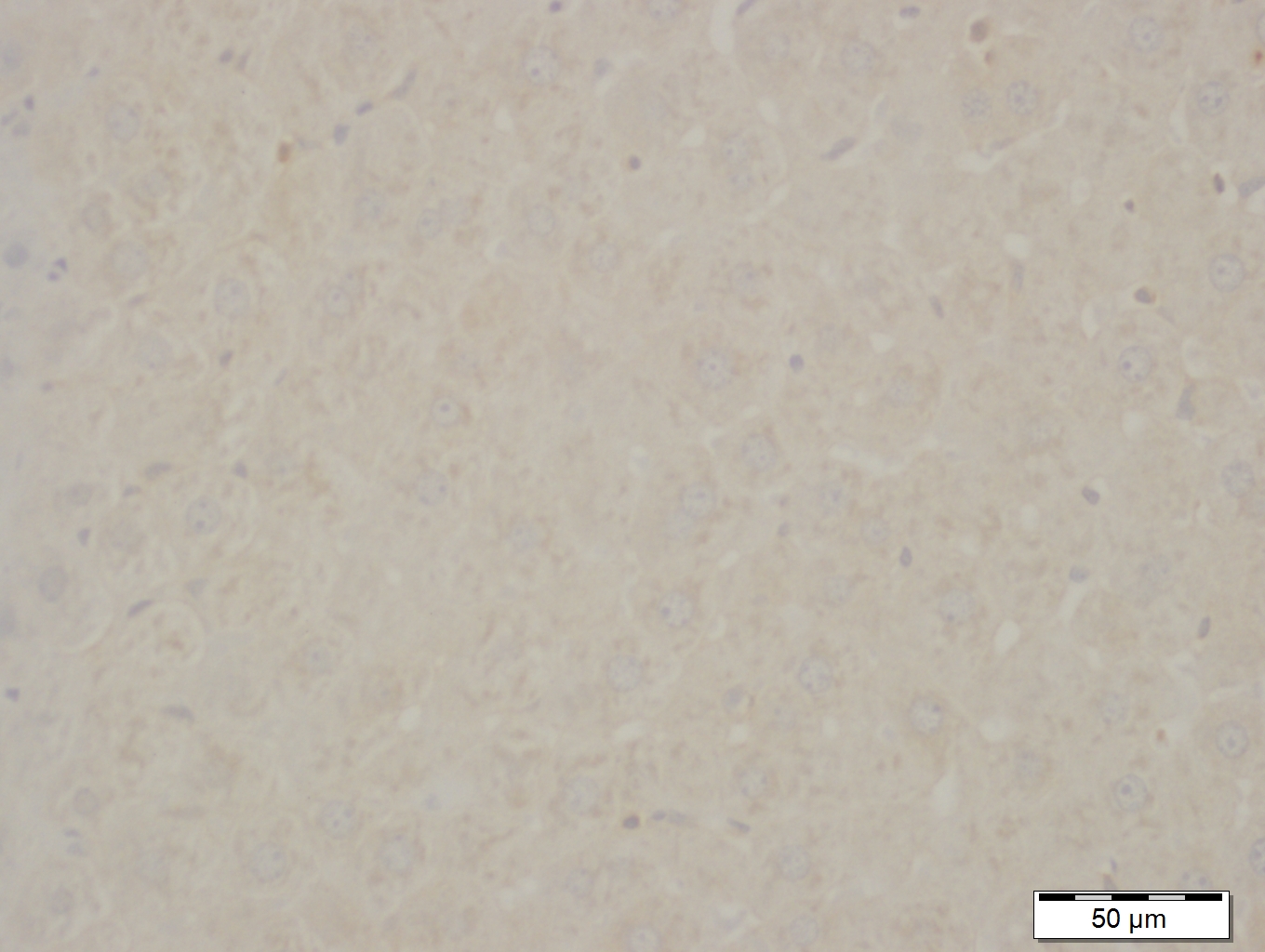

Supplement: Supplementary file 1 [file pharmaceuticals-18-00828-s001.zip › H&E and Immune images/Liver-TNF-KB-Sumayya/Liver-Cont-TNFKB-X400-3 .jpg]

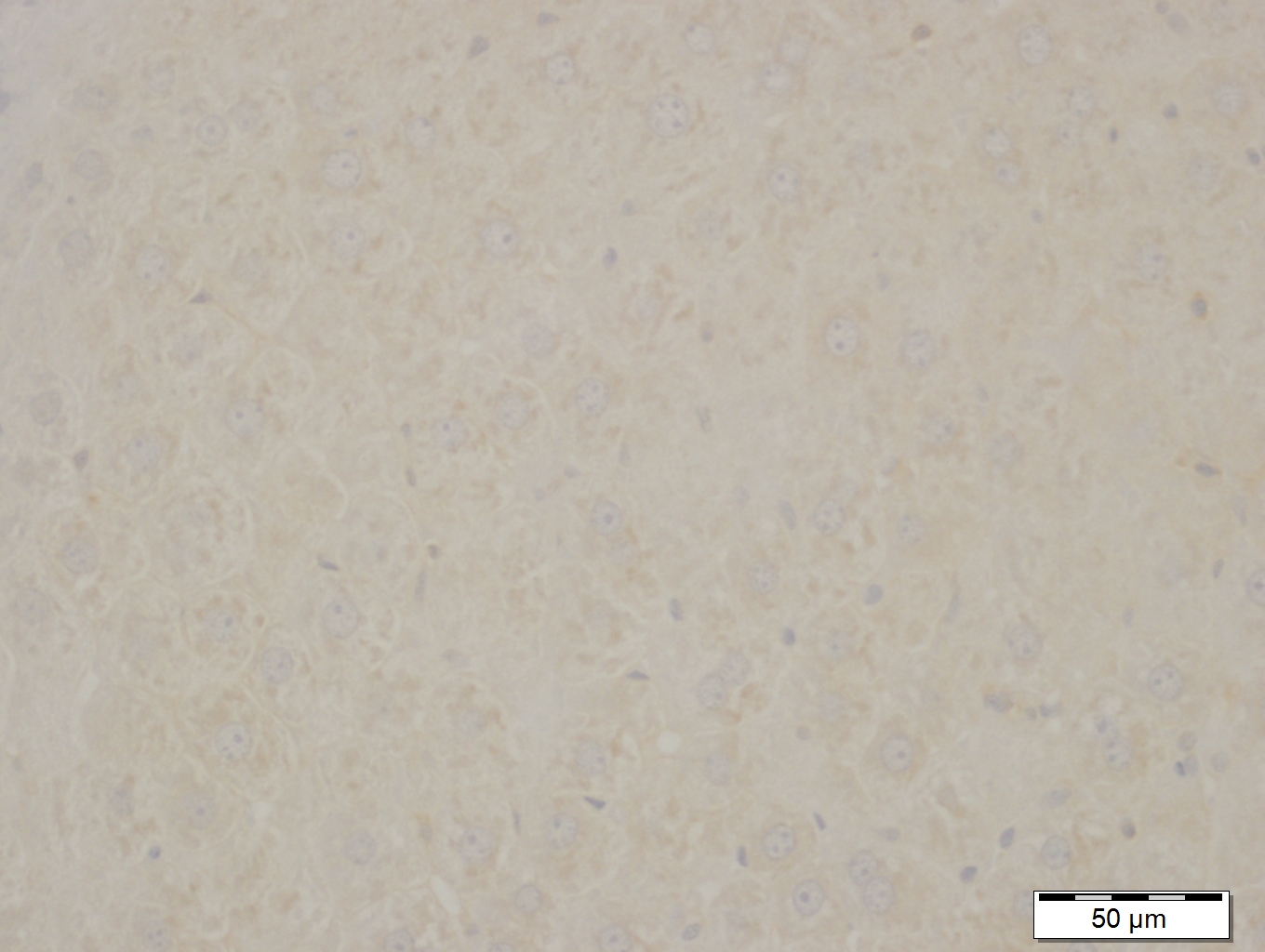

Supplement: Supplementary file 1 [file pharmaceuticals-18-00828-s001.zip › H&E and Immune images/Liver-TNF-KB-Sumayya/Liver-Cont-TNFKB-X400-4 .jpg]

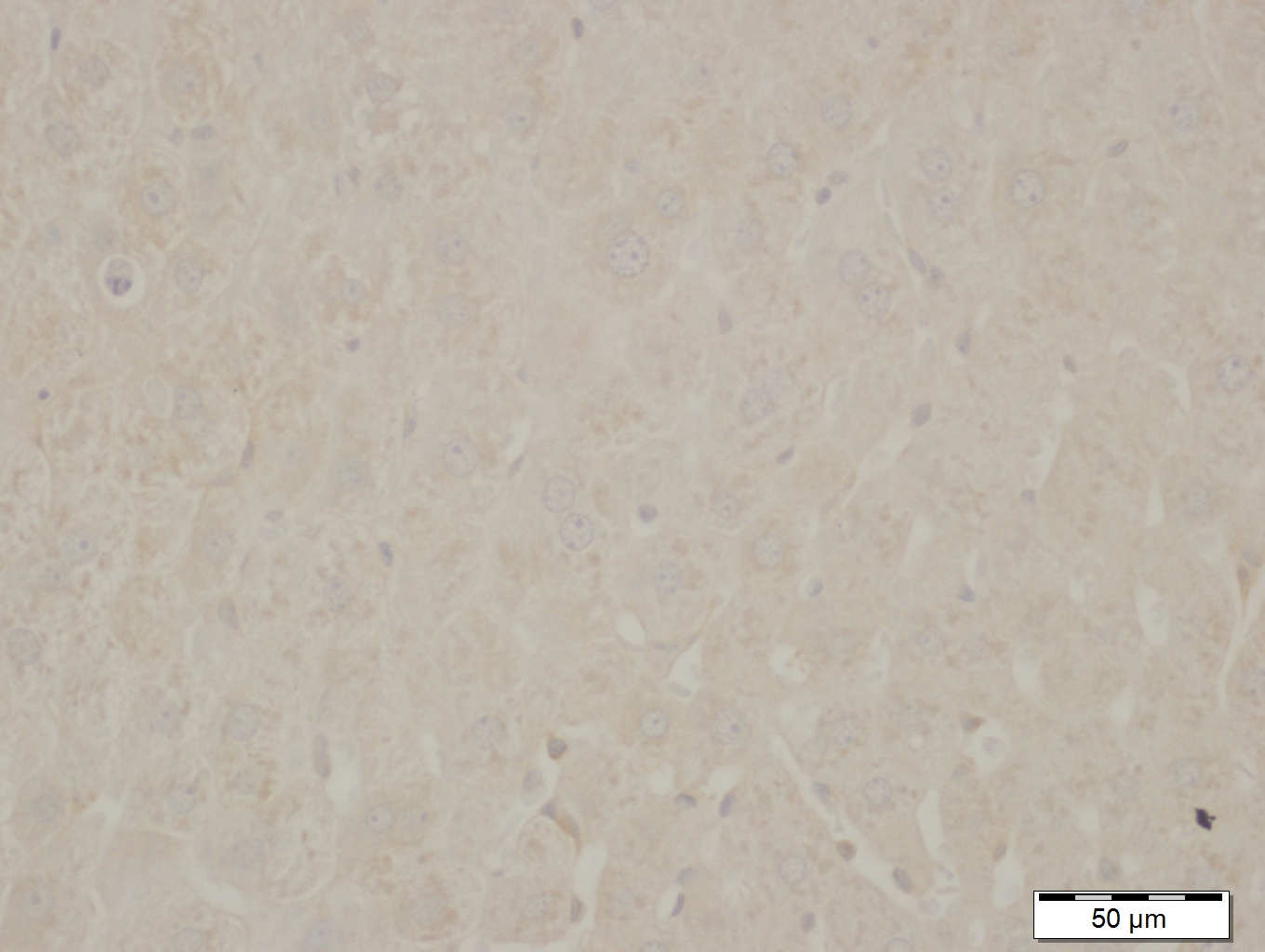

Supplement: Supplementary file 1 [file pharmaceuticals-18-00828-s001.zip › H&E and Immune images/Liver-TNF-KB-Sumayya/Liver-Cont-TNFKB-X400-5 .jpg]

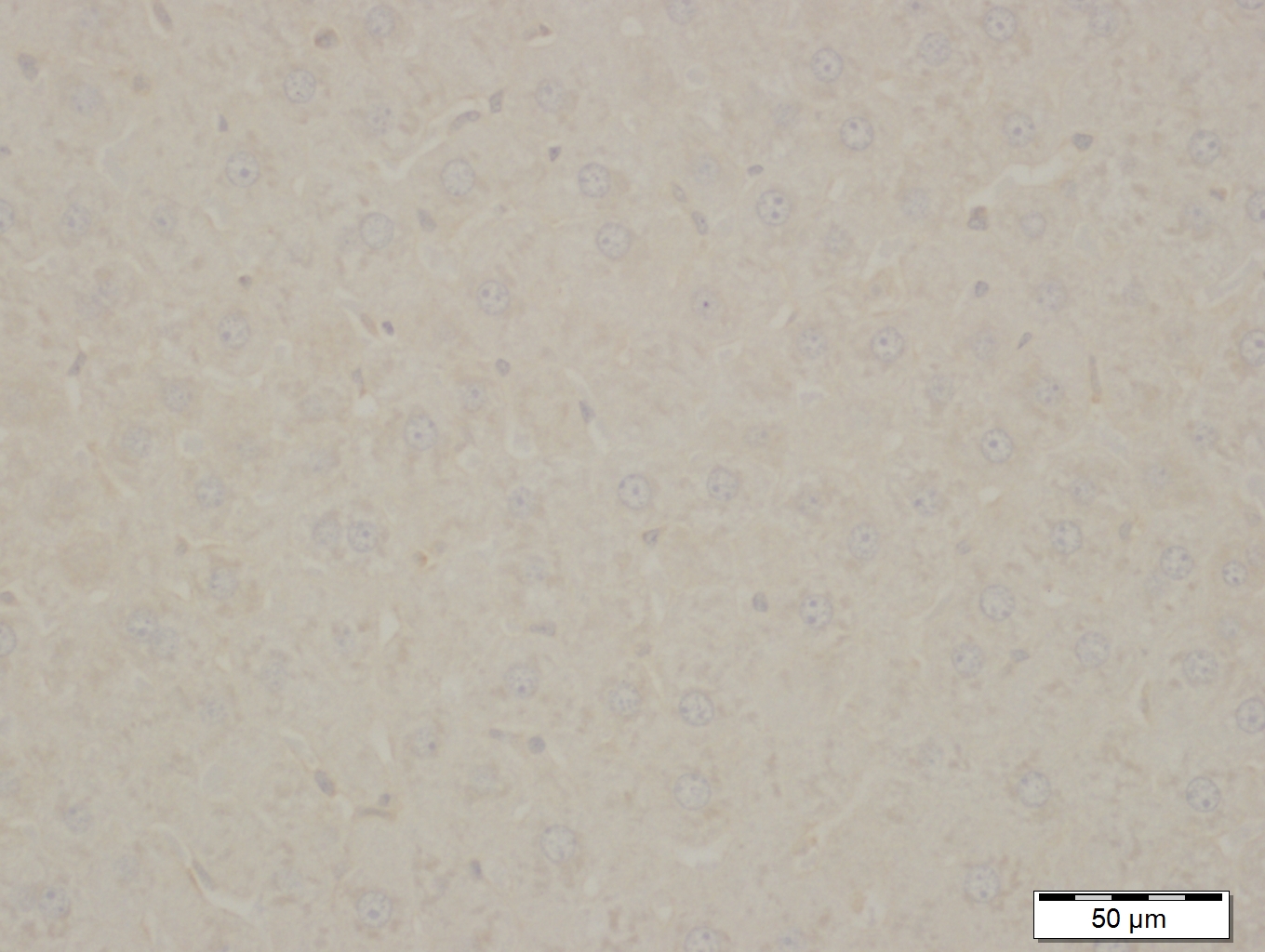

Supplement: Supplementary file 1 [file pharmaceuticals-18-00828-s001.zip › H&E and Immune images/Liver-TNF-KB-Sumayya/Liver-Cont-TNFKB-X400-6 .jpg]

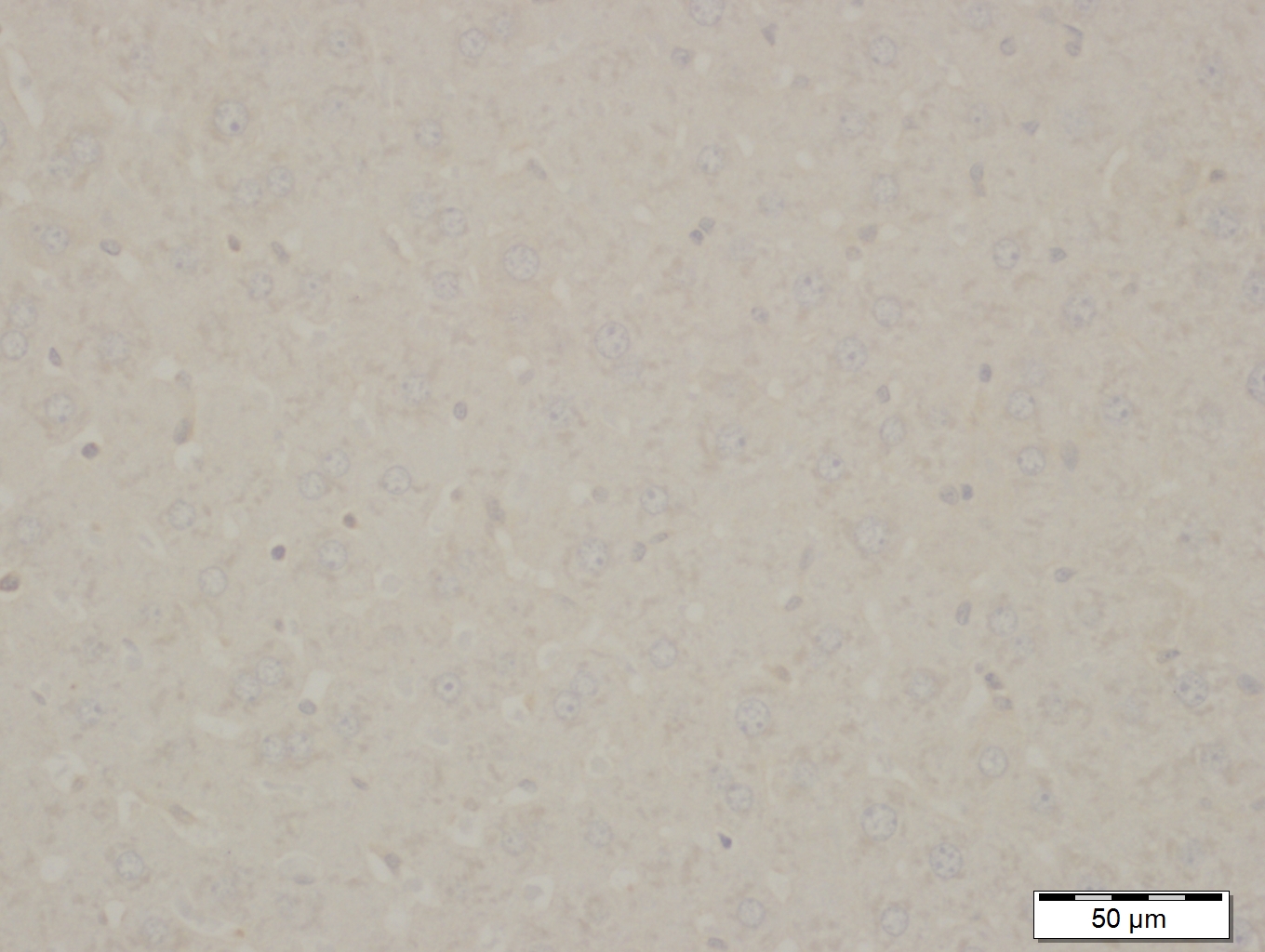

Supplement: Supplementary file 1 [file pharmaceuticals-18-00828-s001.zip › H&E and Immune images/Liver-TNF-KB-Sumayya/Liver-Cont-TNFKB-X400-7 .jpg]

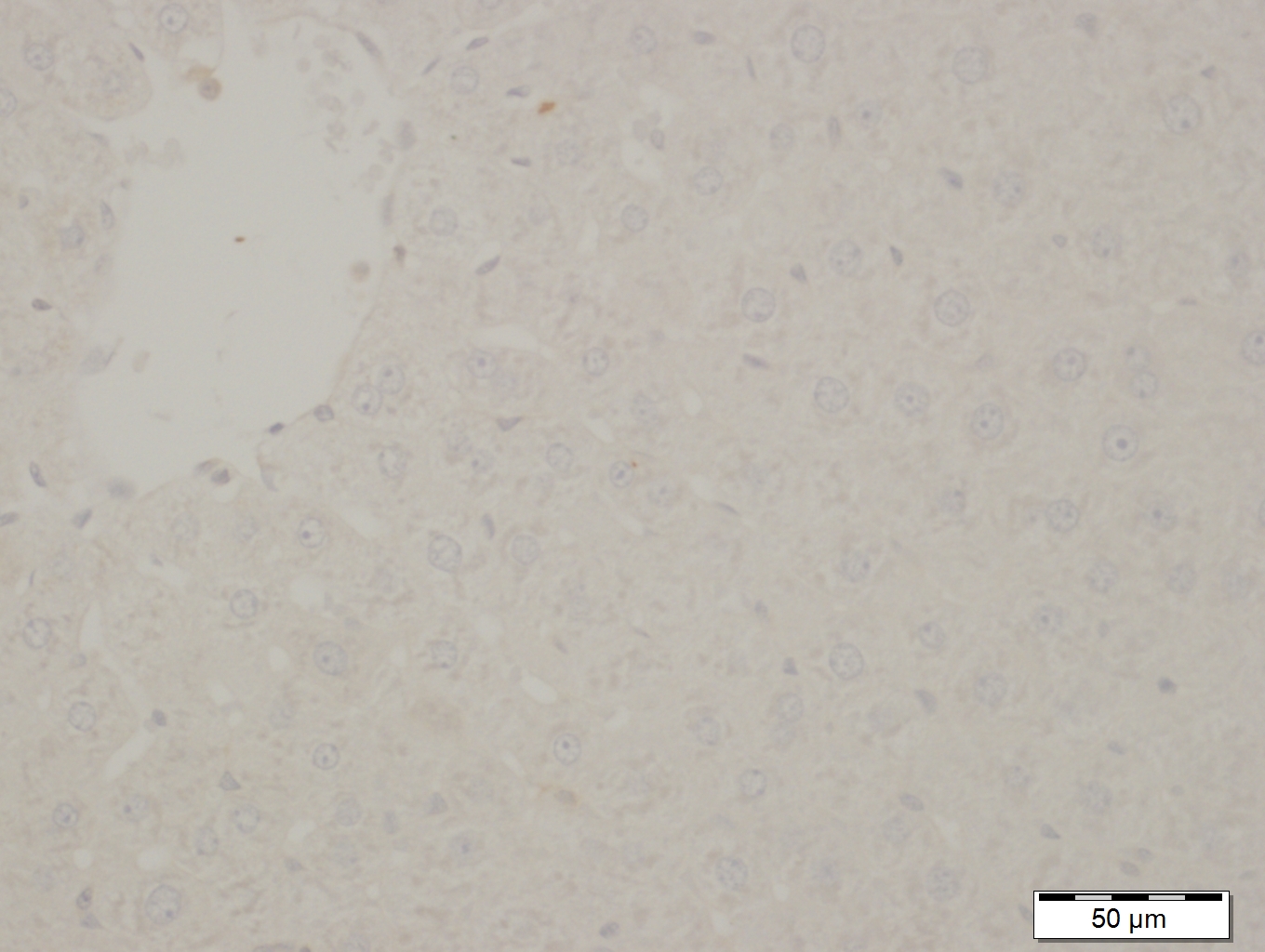

Supplement: Supplementary file 1 [file pharmaceuticals-18-00828-s001.zip › H&E and Immune images/Liver-TNF-KB-Sumayya/Liver-Cont-TNFKB-X400-8 .jpg]

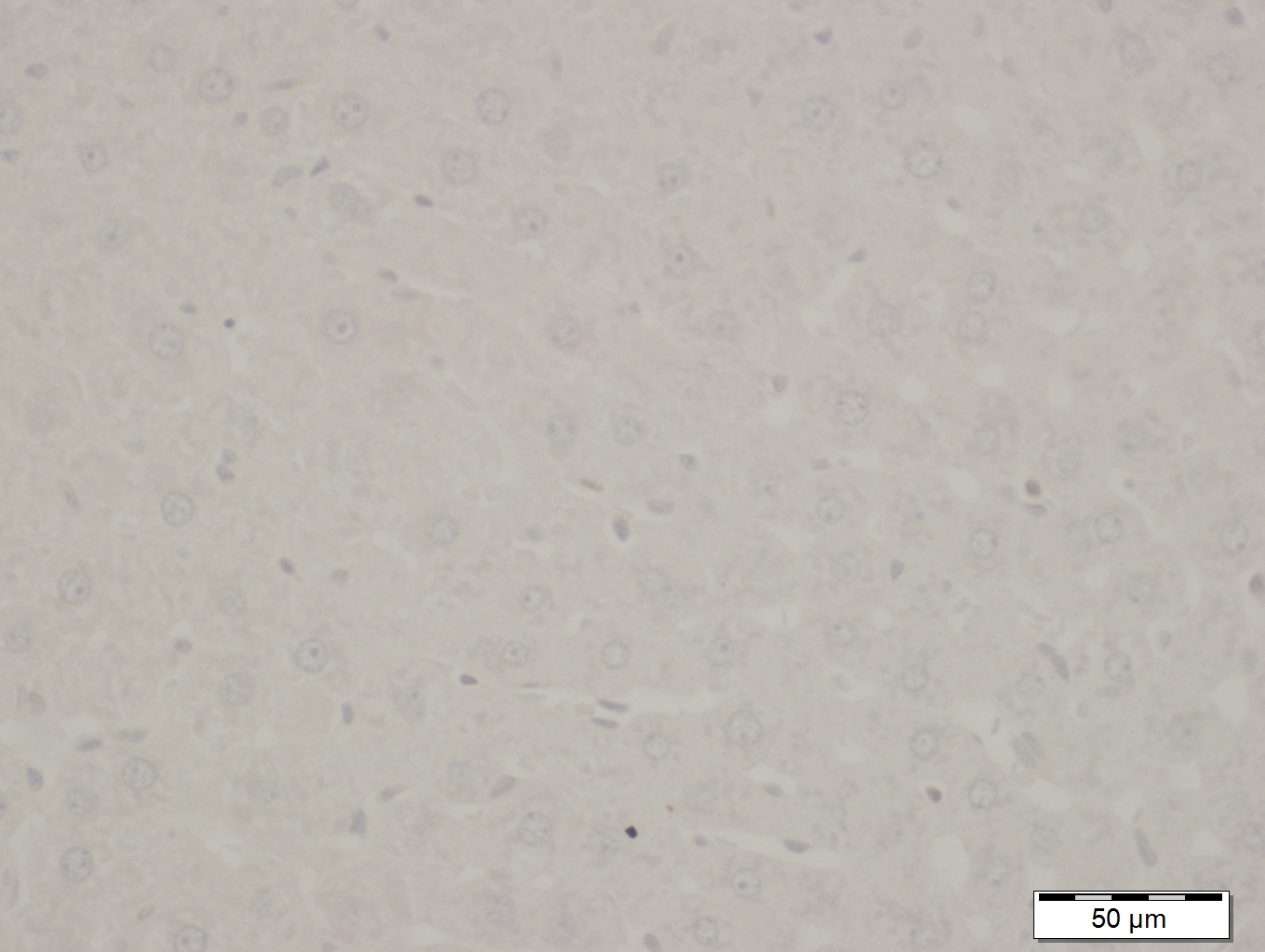

Supplement: Supplementary file 1 [file pharmaceuticals-18-00828-s001.zip › H&E and Immune images/Liver-TNF-KB-Sumayya/Liver-Cont-TNFKB-X400-9 .jpg]

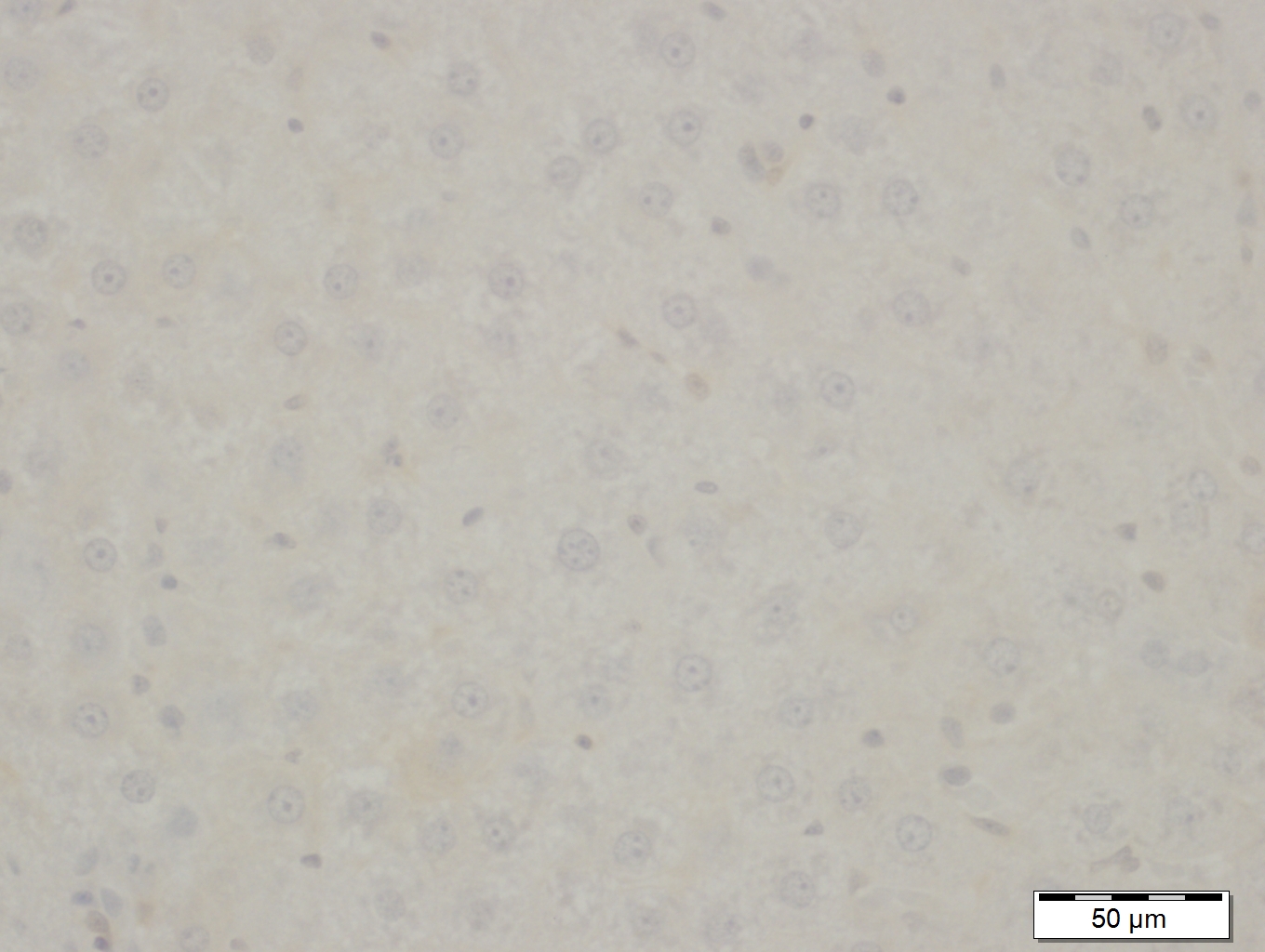

Supplement: Supplementary file 1 [file pharmaceuticals-18-00828-s001.zip › H&E and Immune images/Liver-TNF-KB-Sumayya/Liver-IAA-TNFKB-X400-1 .jpg]

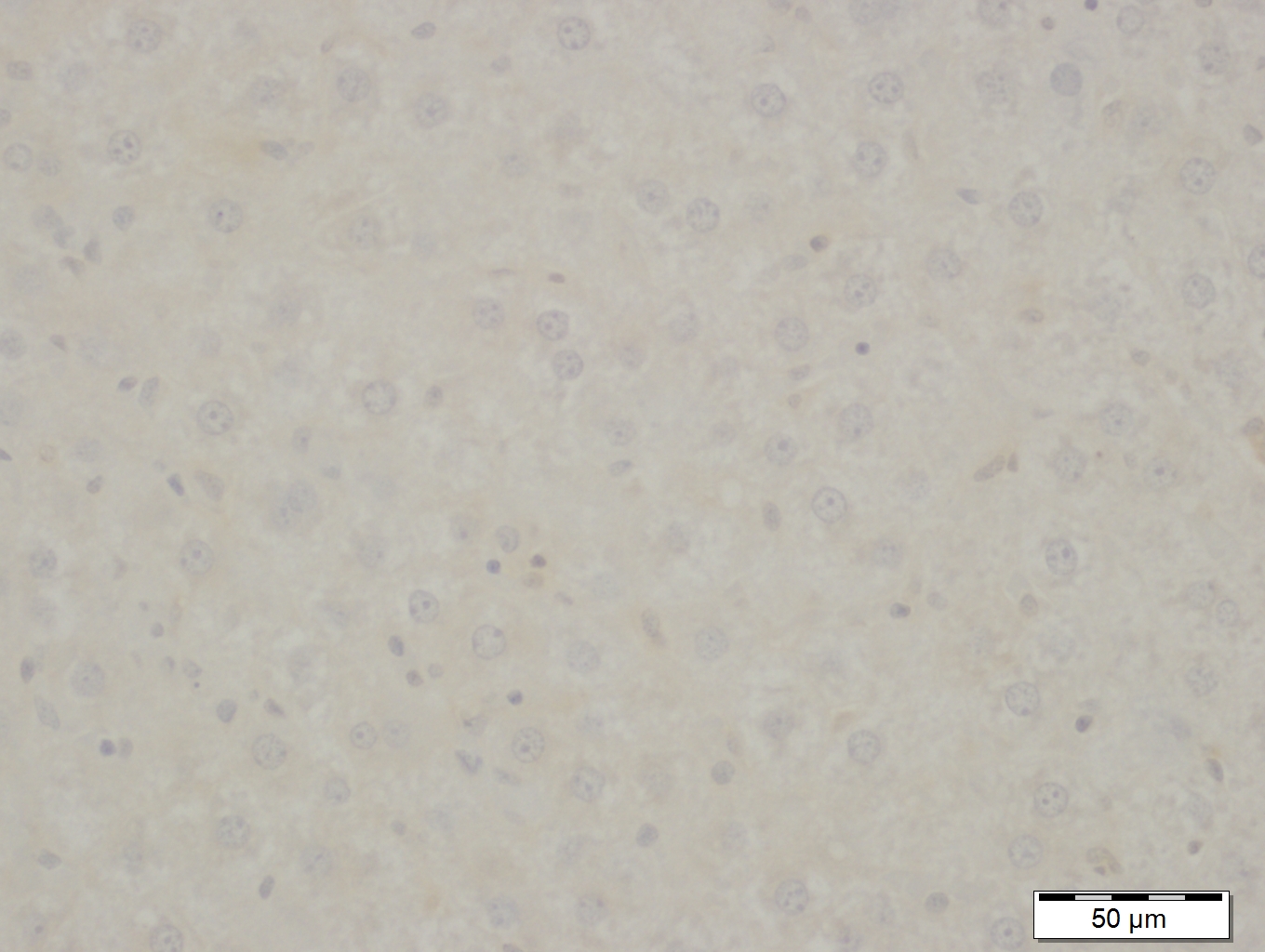

Supplement: Supplementary file 1 [file pharmaceuticals-18-00828-s001.zip › H&E and Immune images/Liver-TNF-KB-Sumayya/Liver-IAA-TNFKB-X400-2 .jpg]

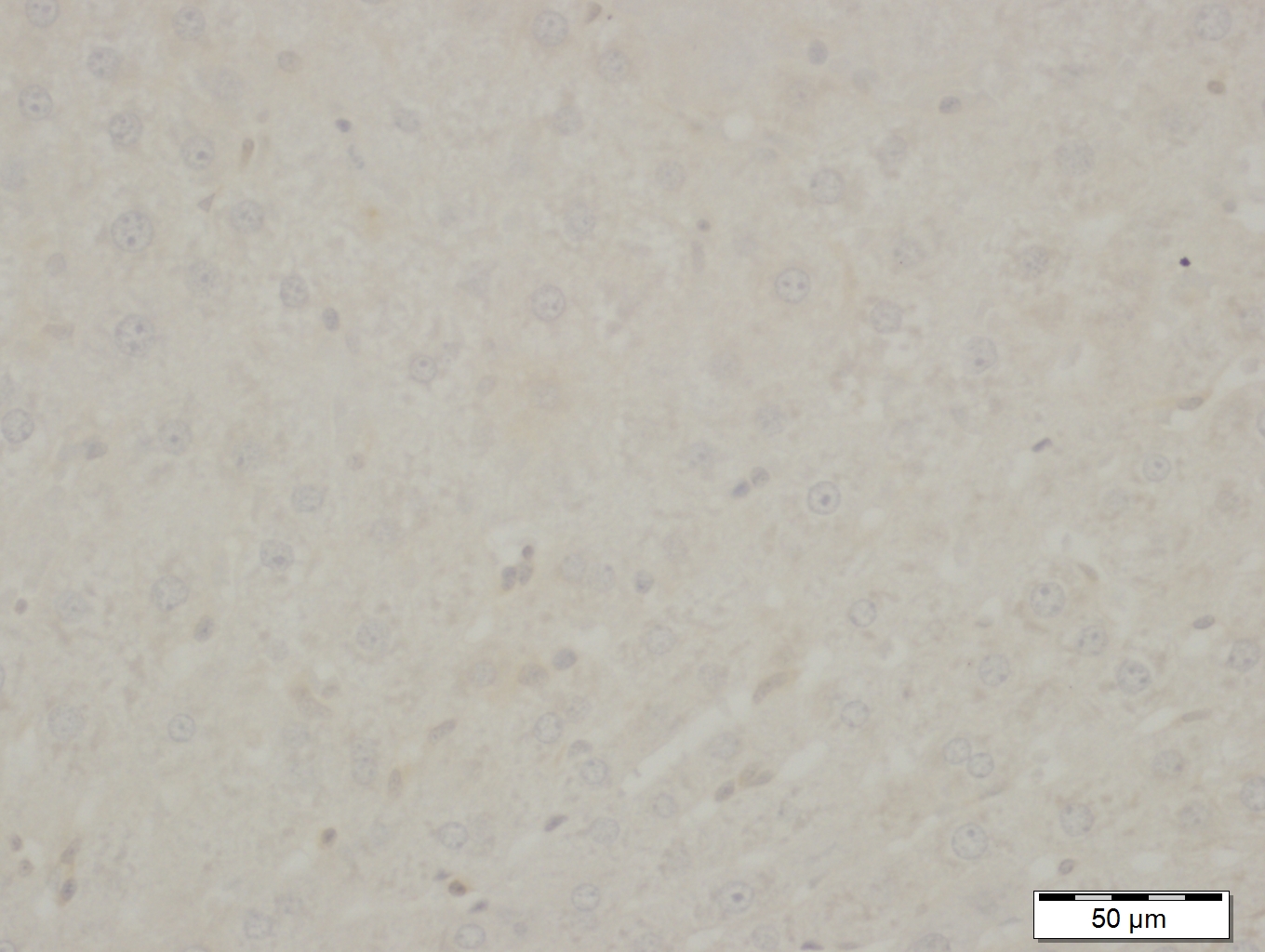

Supplement: Supplementary file 1 [file pharmaceuticals-18-00828-s001.zip › H&E and Immune images/Liver-TNF-KB-Sumayya/Liver-IAA-TNFKB-X400-3 .jpg]

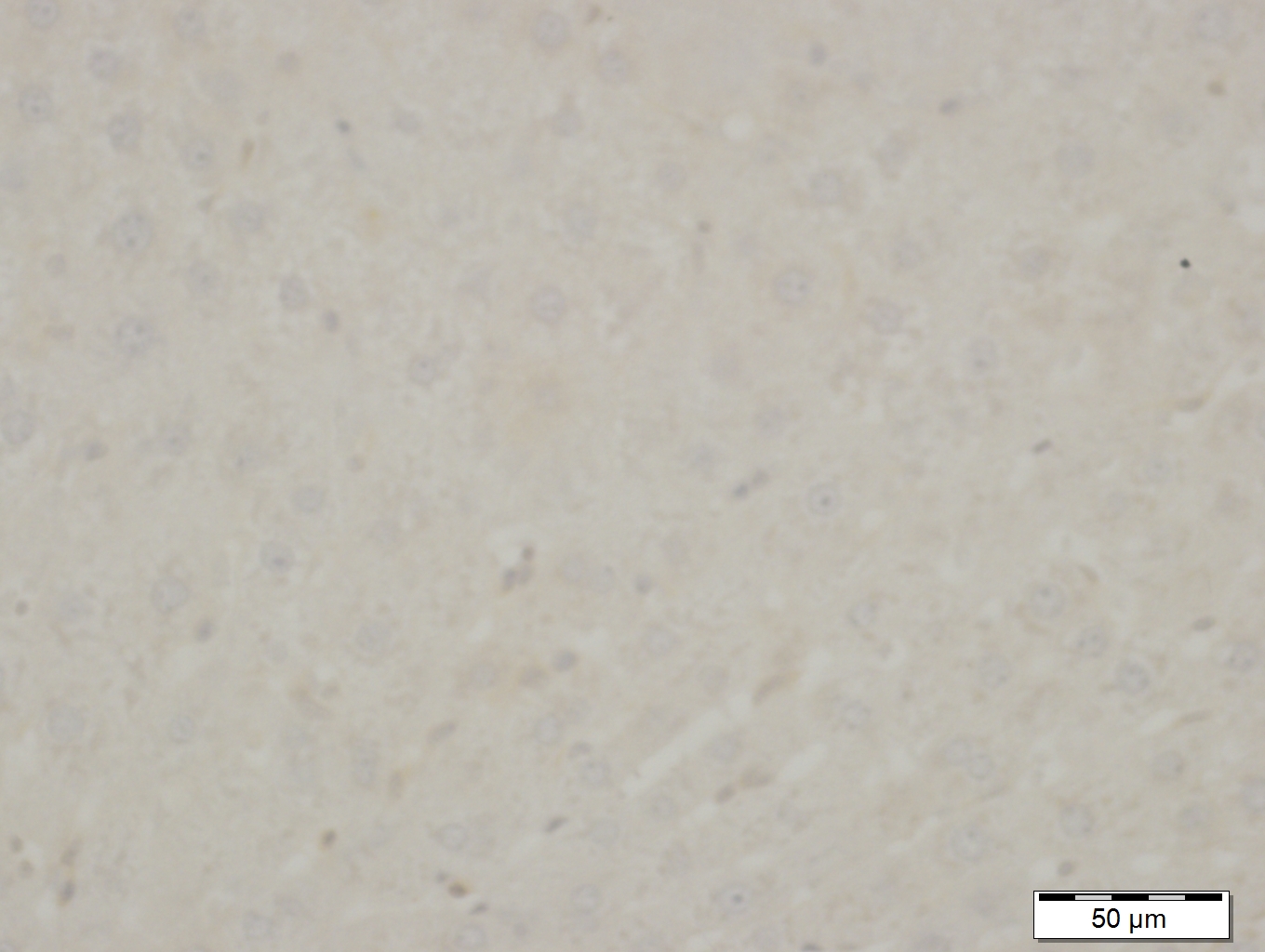

Supplement: Supplementary file 1 [file pharmaceuticals-18-00828-s001.zip › H&E and Immune images/Liver-TNF-KB-Sumayya/Liver-IAA-TNFKB-X400-4 .jpg]

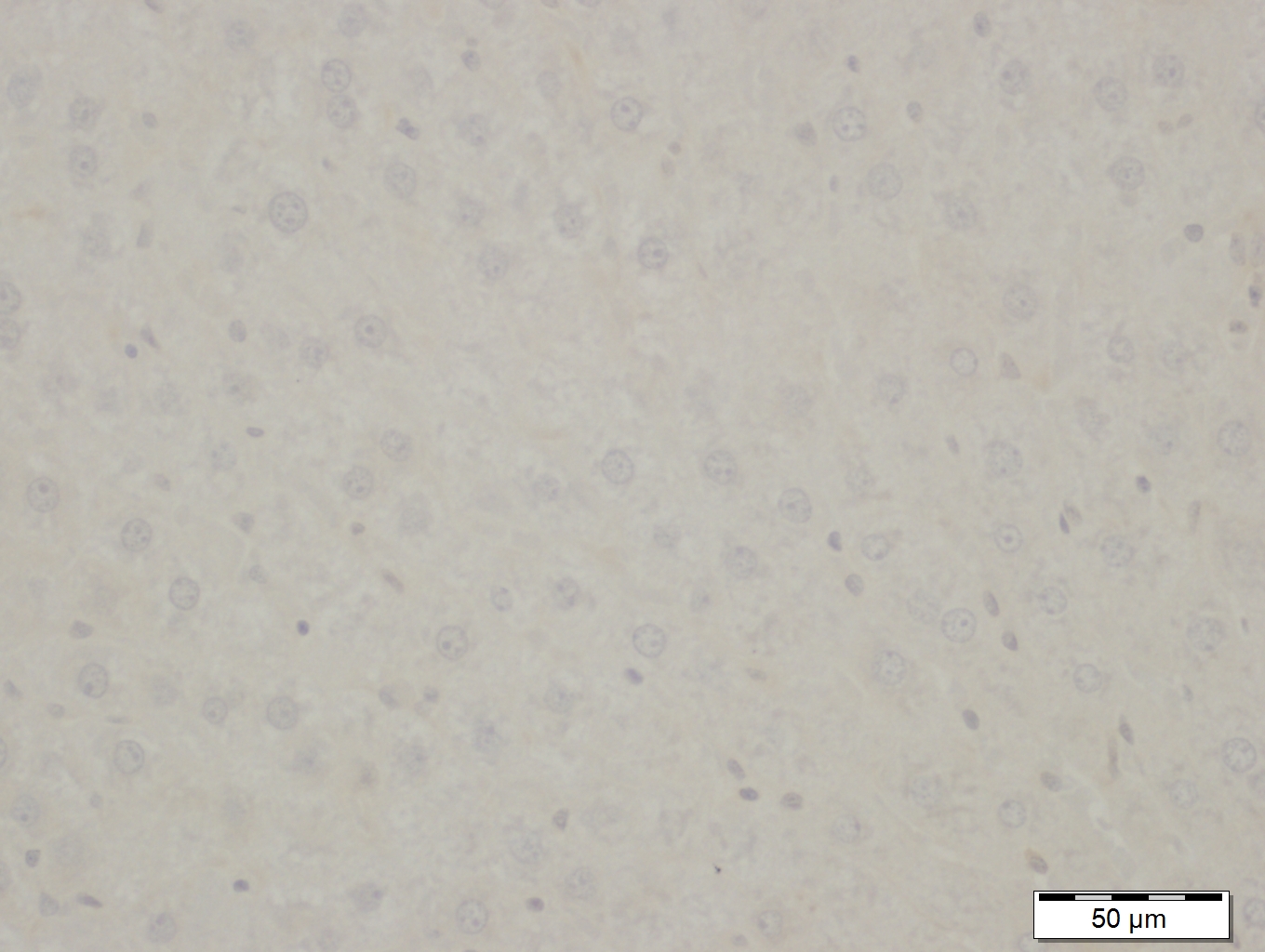

Supplement: Supplementary file 1 [file pharmaceuticals-18-00828-s001.zip › H&E and Immune images/Liver-TNF-KB-Sumayya/Liver-IAA-TNFKB-X400-5 .jpg]

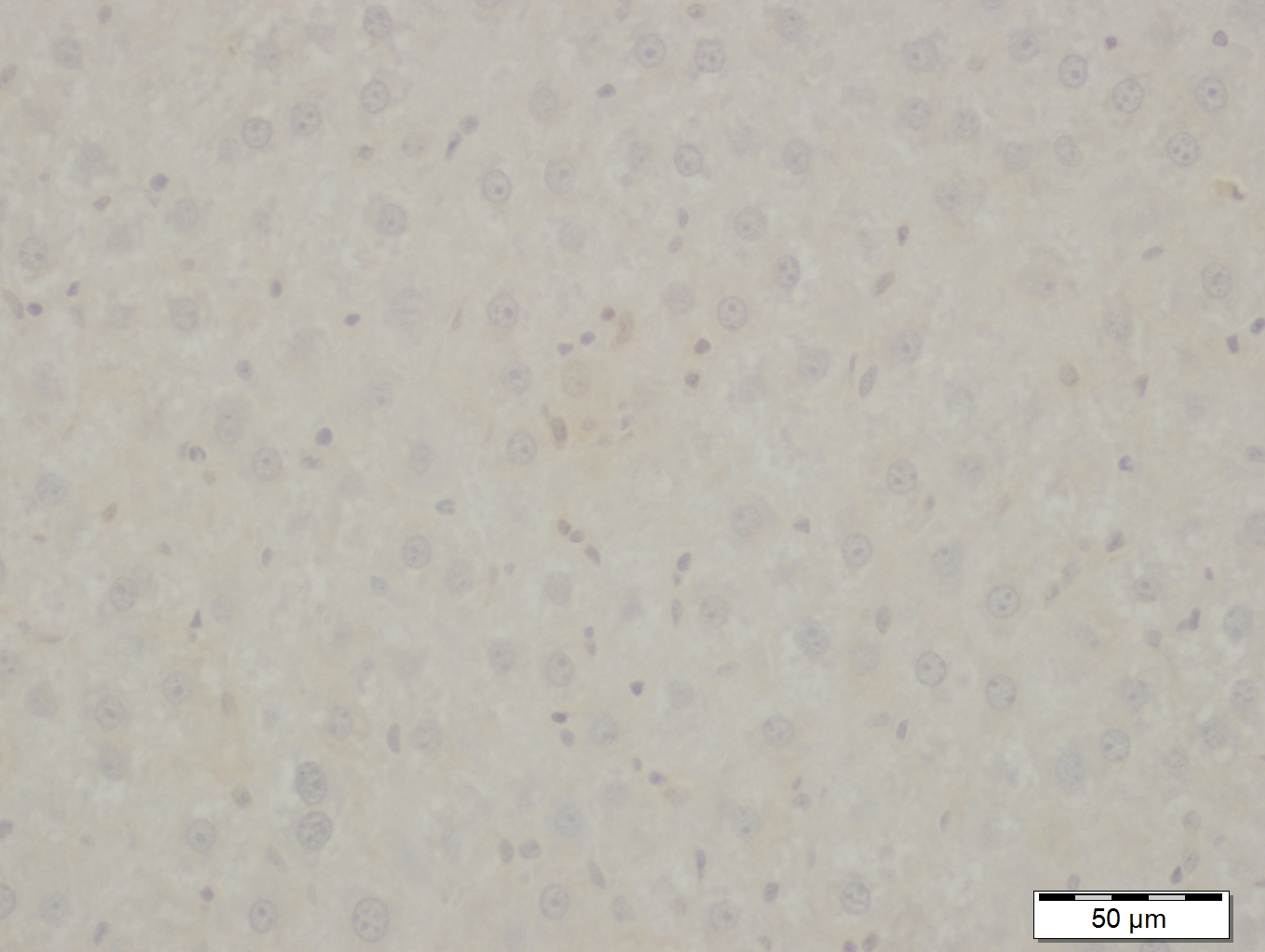

Supplement: Supplementary file 1 [file pharmaceuticals-18-00828-s001.zip › H&E and Immune images/Liver-TNF-KB-Sumayya/Liver-IAA-TNFKB-X400-6 .jpg]

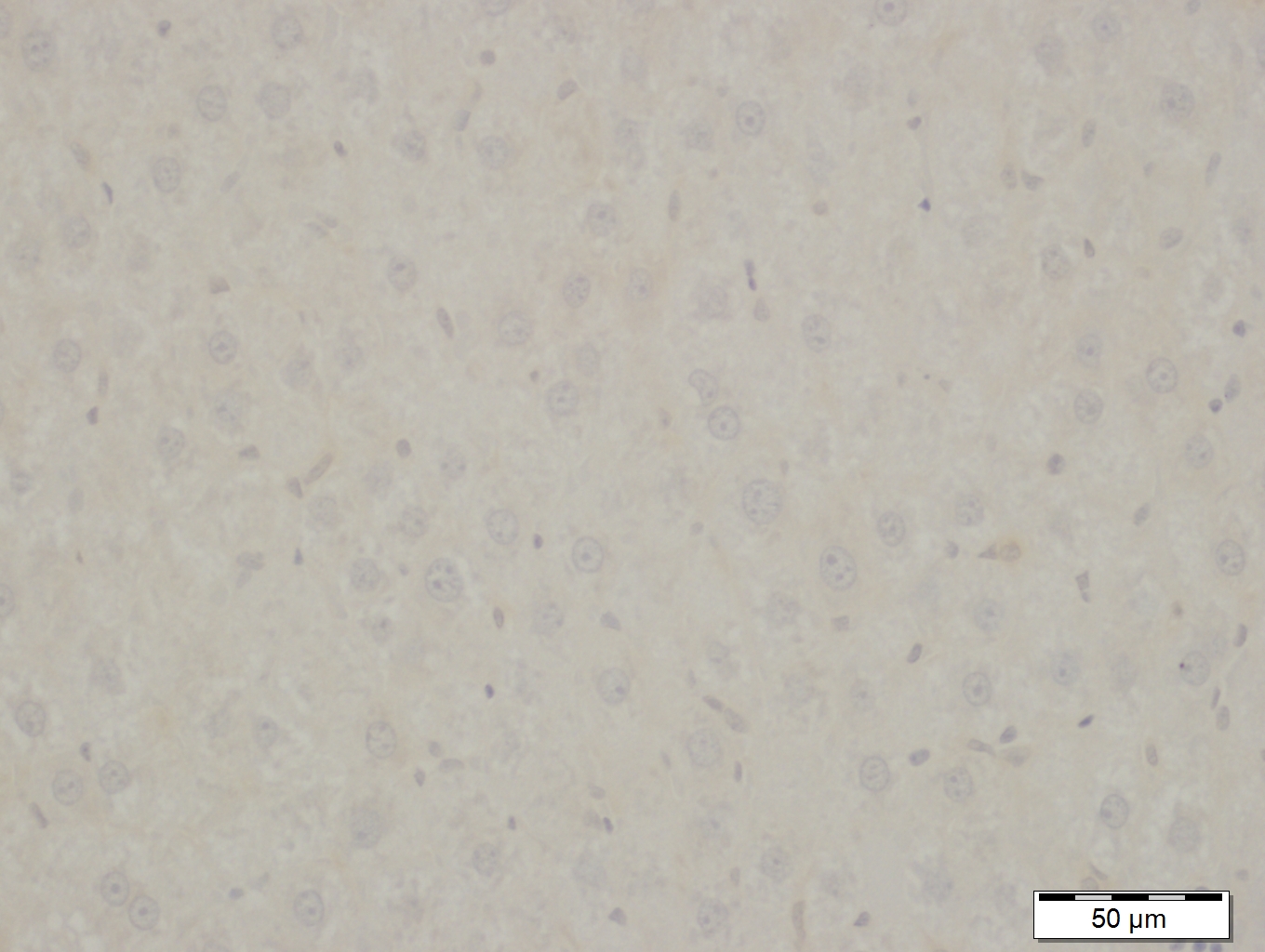

Supplement: Supplementary file 1 [file pharmaceuticals-18-00828-s001.zip › H&E and Immune images/Liver-TNF-KB-Sumayya/Liver-IAA-TNFKB-X400-7 .jpg]

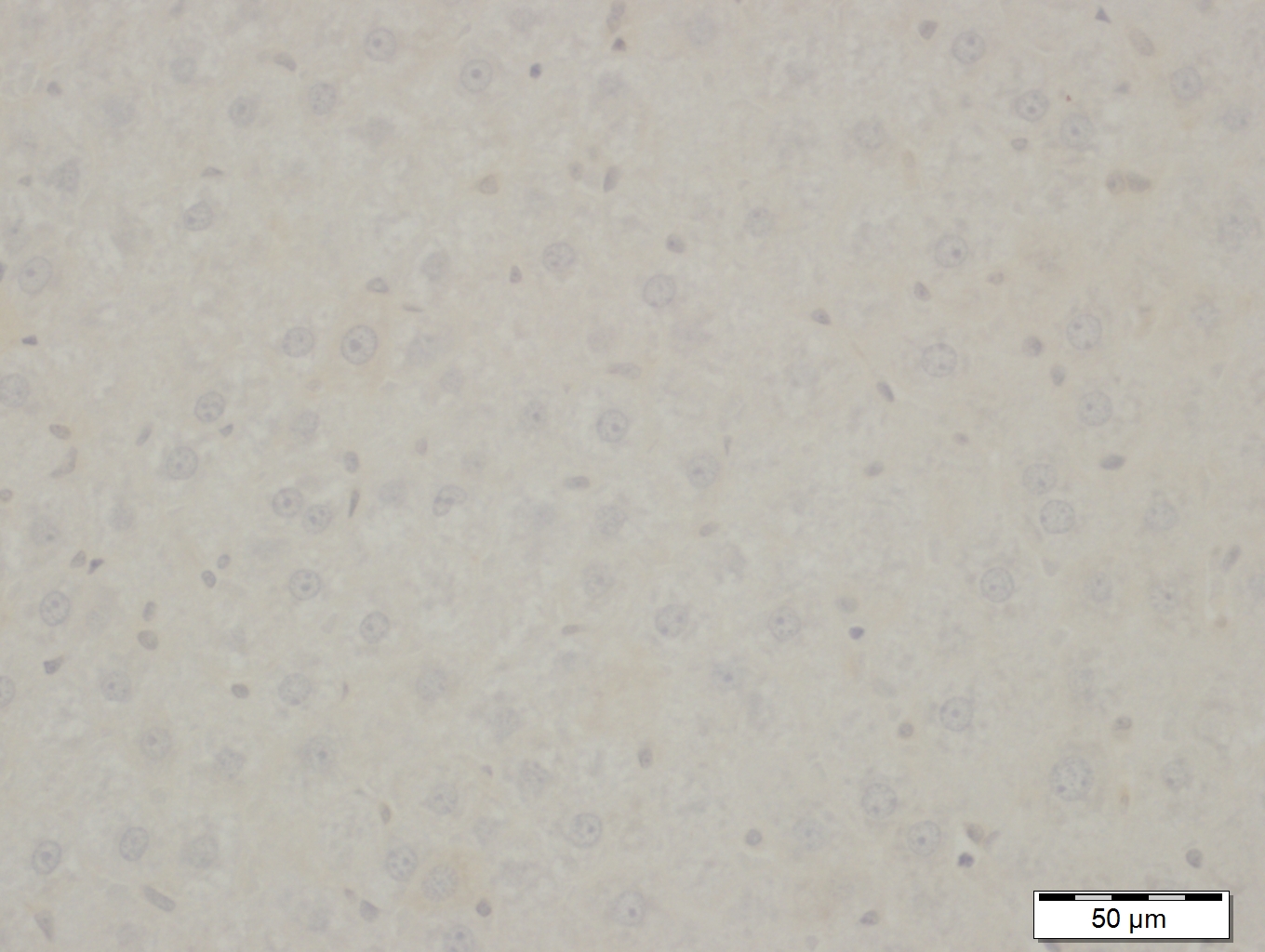

Supplement: Supplementary file 1 [file pharmaceuticals-18-00828-s001.zip › H&E and Immune images/Liver-TNF-KB-Sumayya/Liver-IAA-TNFKB-X400-8 .jpg]

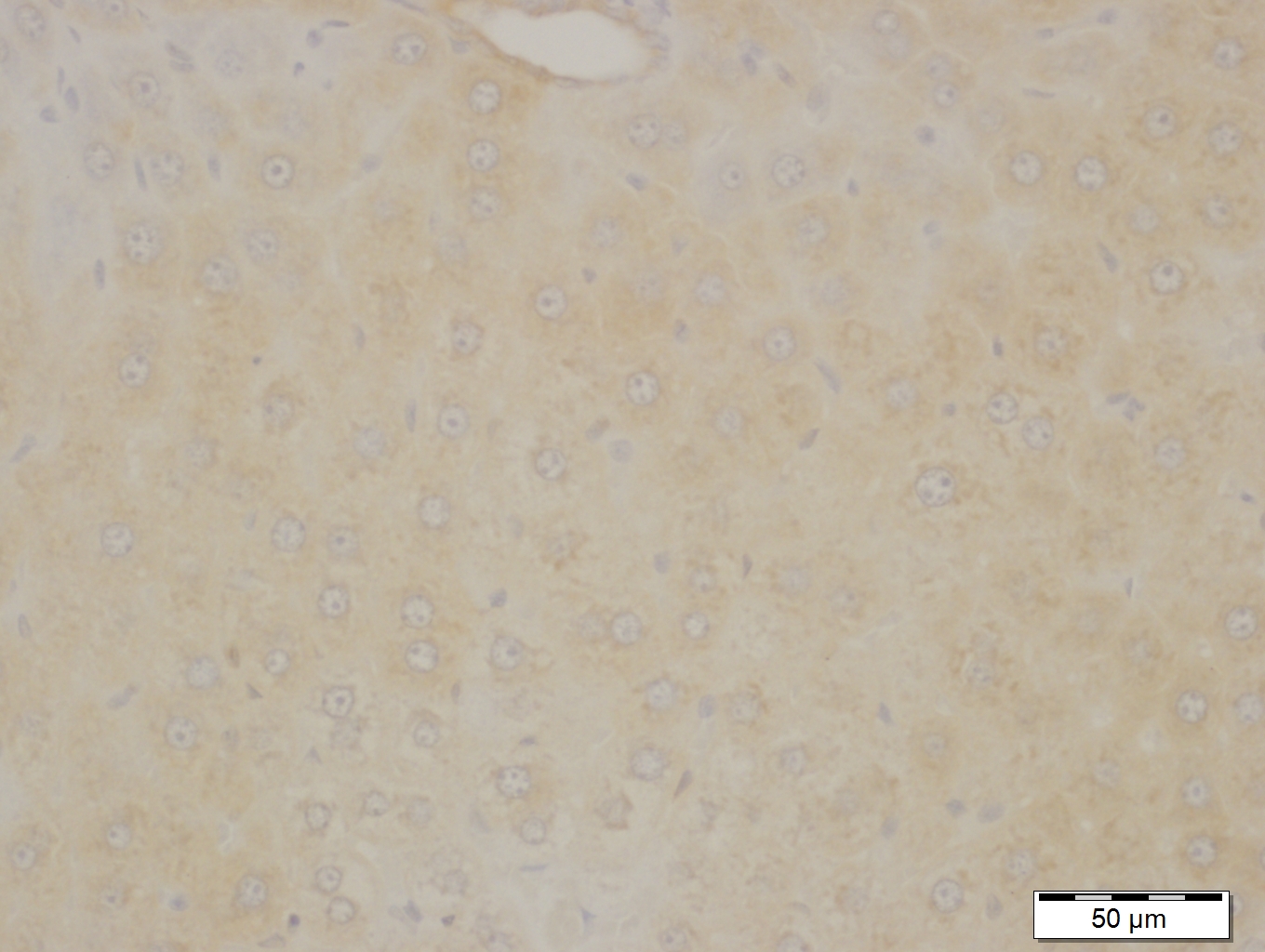

Supplement: Supplementary file 1 [file pharmaceuticals-18-00828-s001.zip › H&E and Immune images/Liver-TNF-KB-Sumayya/Liver-MTX+IAA-TNFKB-X400-1 .jpg]

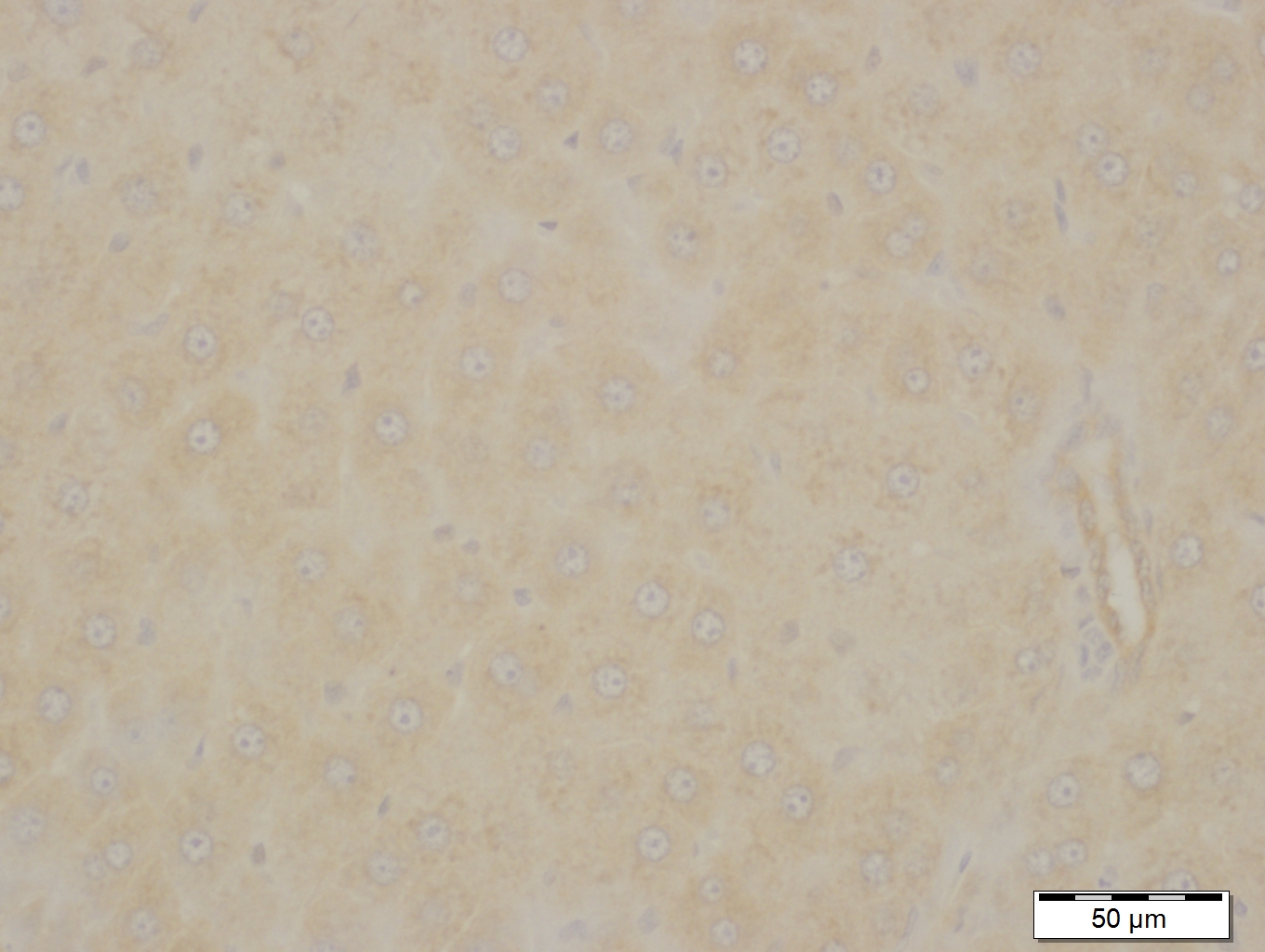

Supplement: Supplementary file 1 [file pharmaceuticals-18-00828-s001.zip › H&E and Immune images/Liver-TNF-KB-Sumayya/Liver-MTX+IAA-TNFKB-X400-2 .jpg]

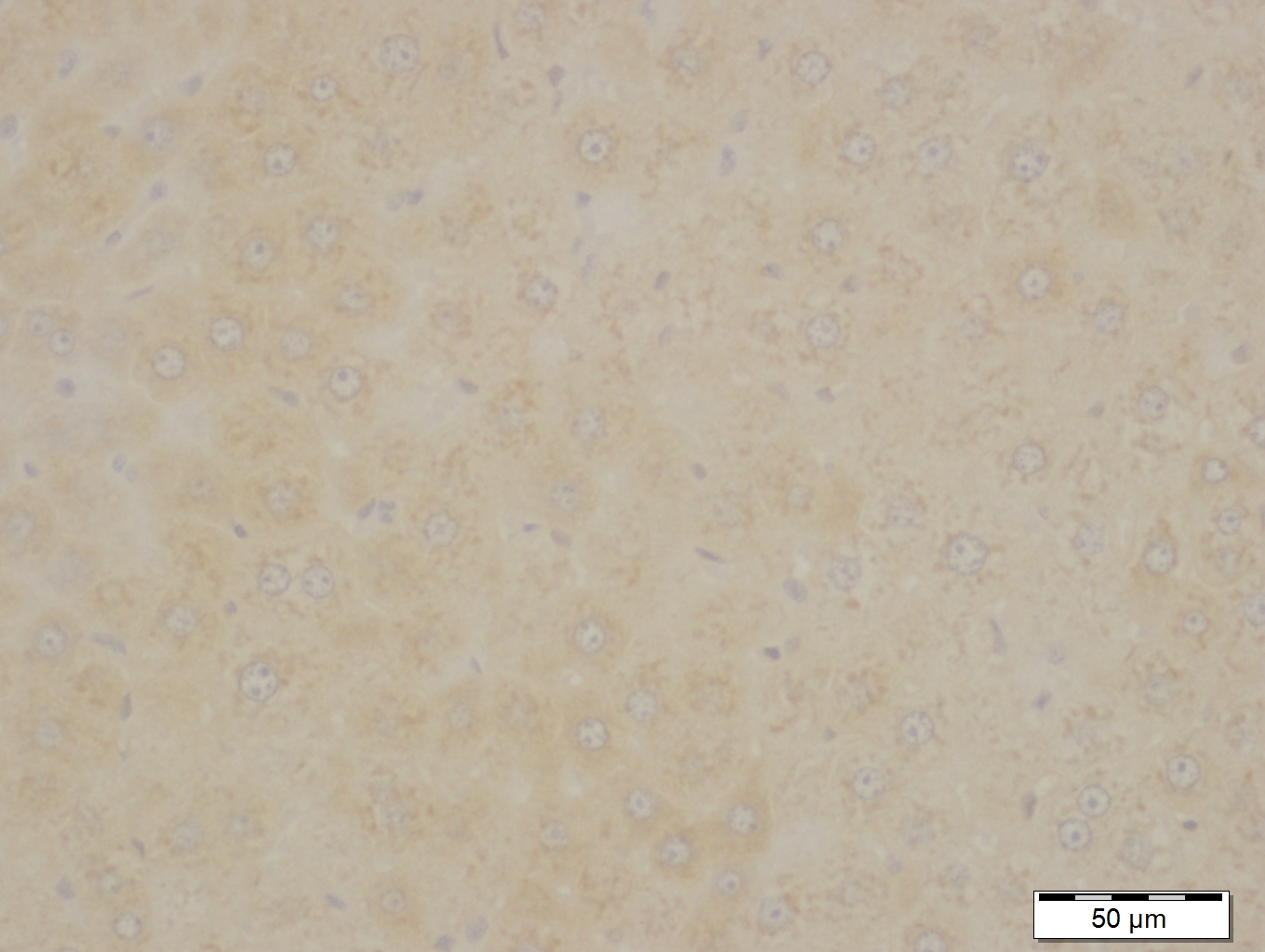

Supplement: Supplementary file 1 [file pharmaceuticals-18-00828-s001.zip › H&E and Immune images/Liver-TNF-KB-Sumayya/Liver-MTX+IAA-TNFKB-X400-3 .jpg]

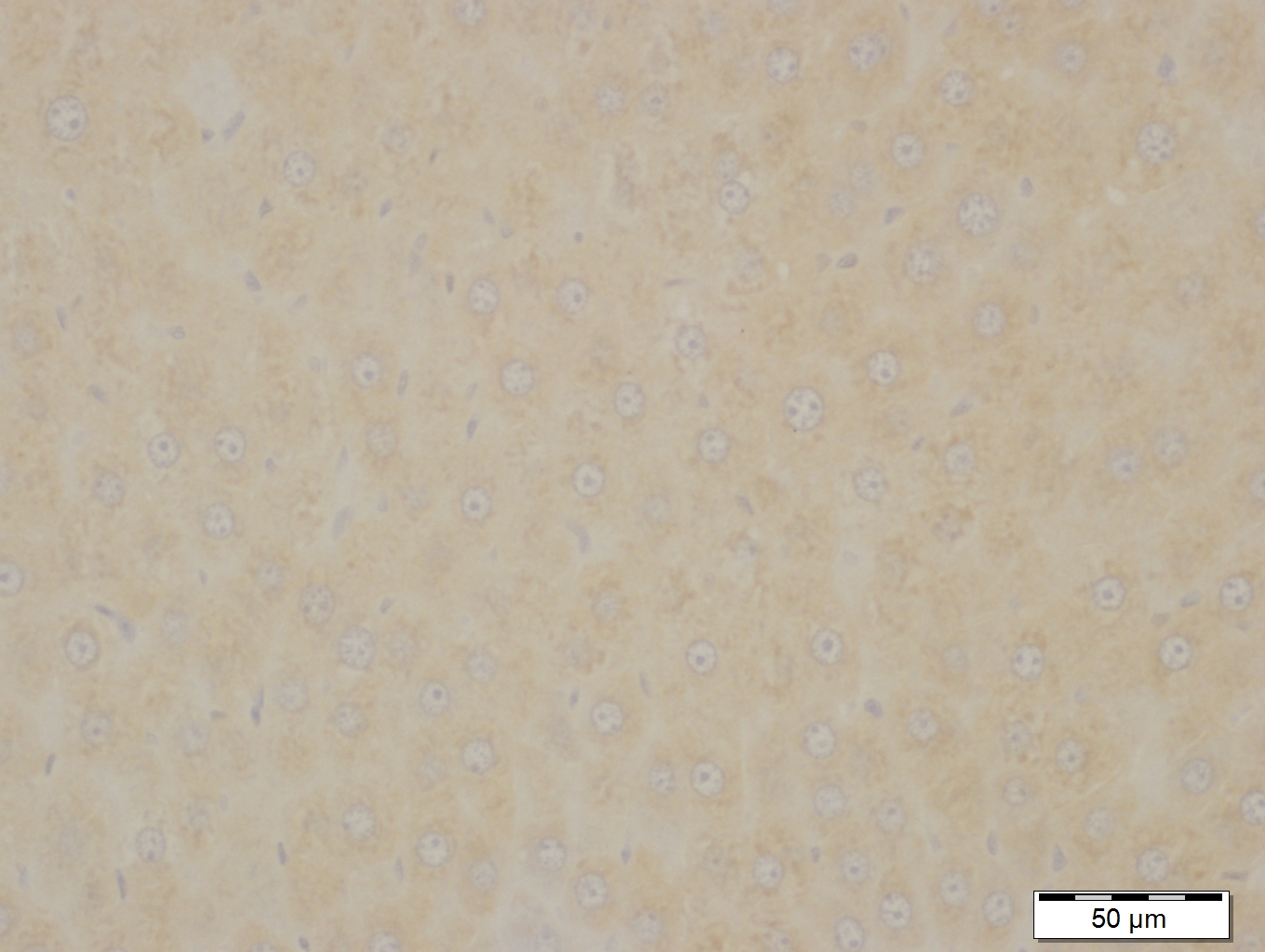

Supplement: Supplementary file 1 [file pharmaceuticals-18-00828-s001.zip › H&E and Immune images/Liver-TNF-KB-Sumayya/Liver-MTX+IAA-TNFKB-X400-4 .jpg]

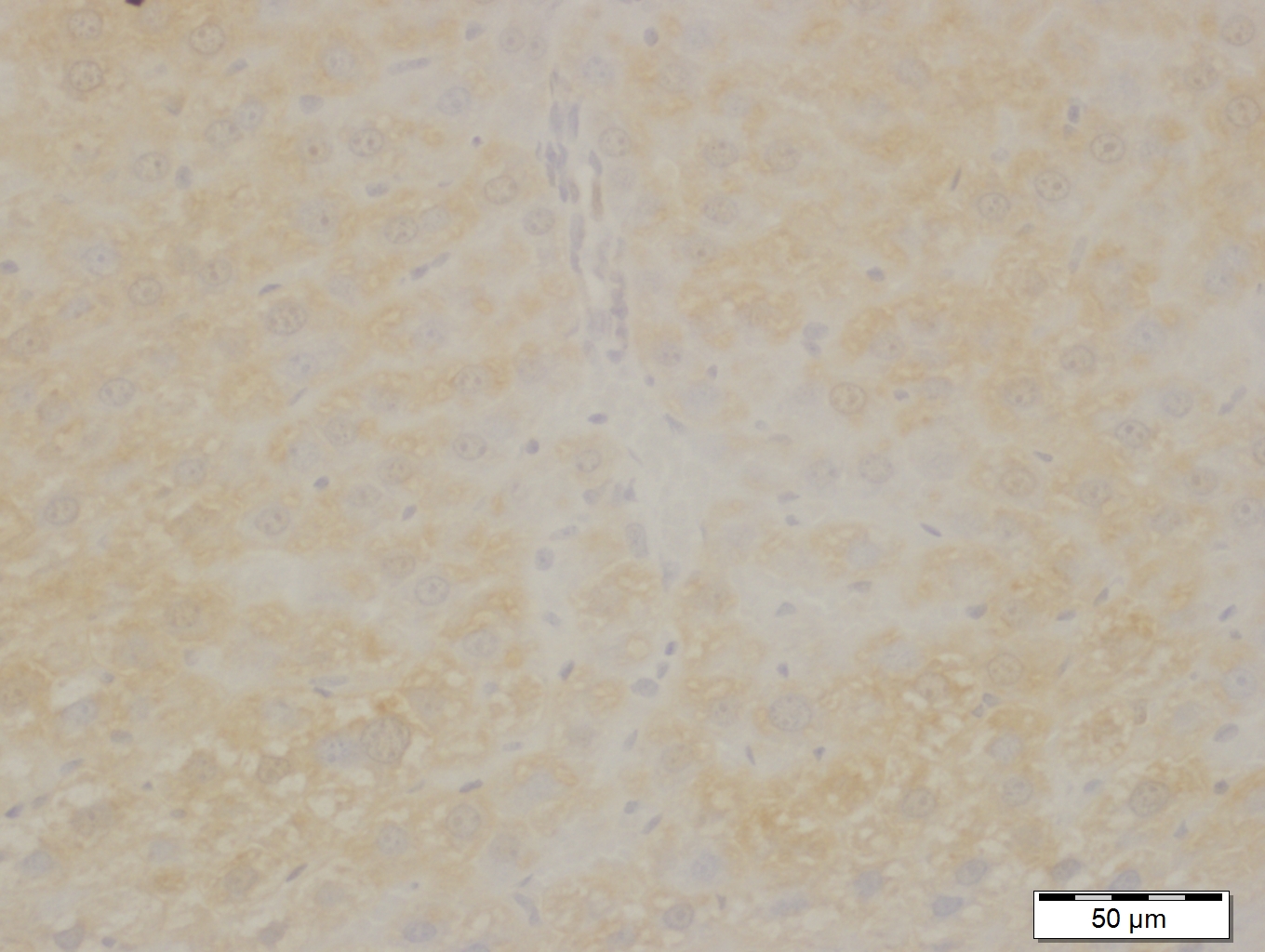

Supplement: Supplementary file 1 [file pharmaceuticals-18-00828-s001.zip › H&E and Immune images/Liver-TNF-KB-Sumayya/Liver-MTX+IAA-TNFKB-X400-5 .jpg]

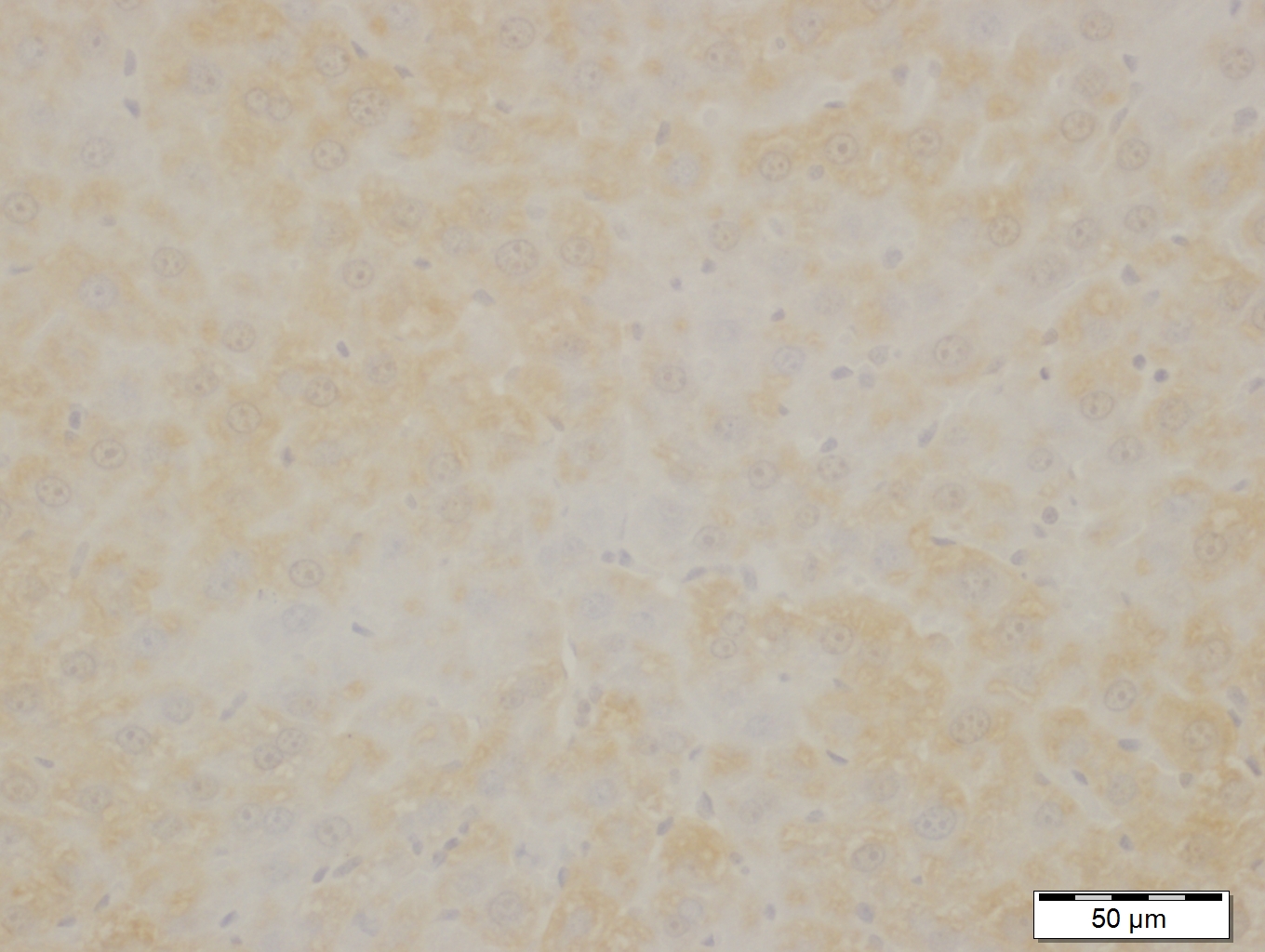

Supplement: Supplementary file 1 [file pharmaceuticals-18-00828-s001.zip › H&E and Immune images/Liver-TNF-KB-Sumayya/Liver-MTX+IAA-TNFKB-X400-6 .jpg]

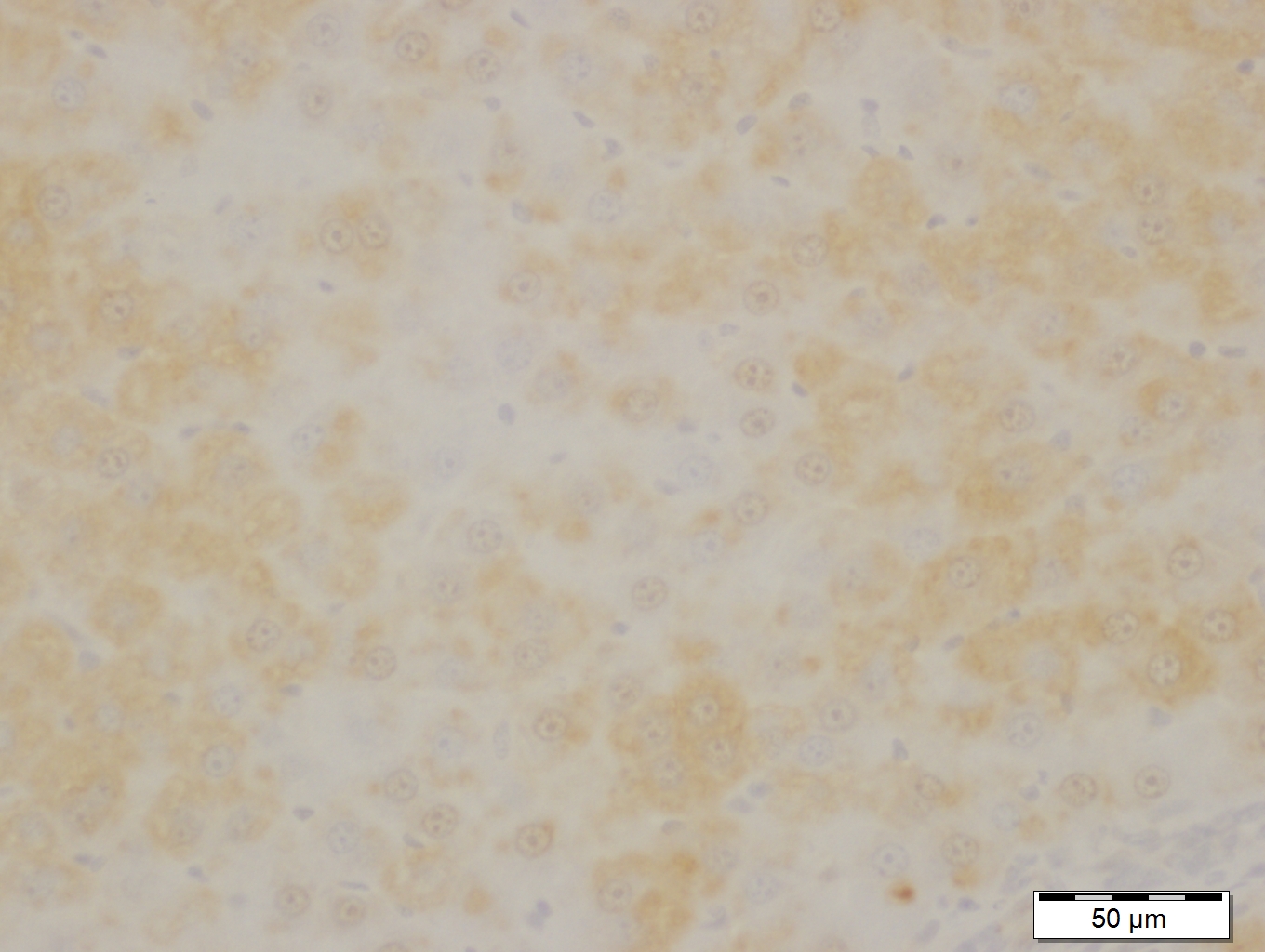

Supplement: Supplementary file 1 [file pharmaceuticals-18-00828-s001.zip › H&E and Immune images/Liver-TNF-KB-Sumayya/Liver-MTX+IAA-TNFKB-X400-7 .jpg]

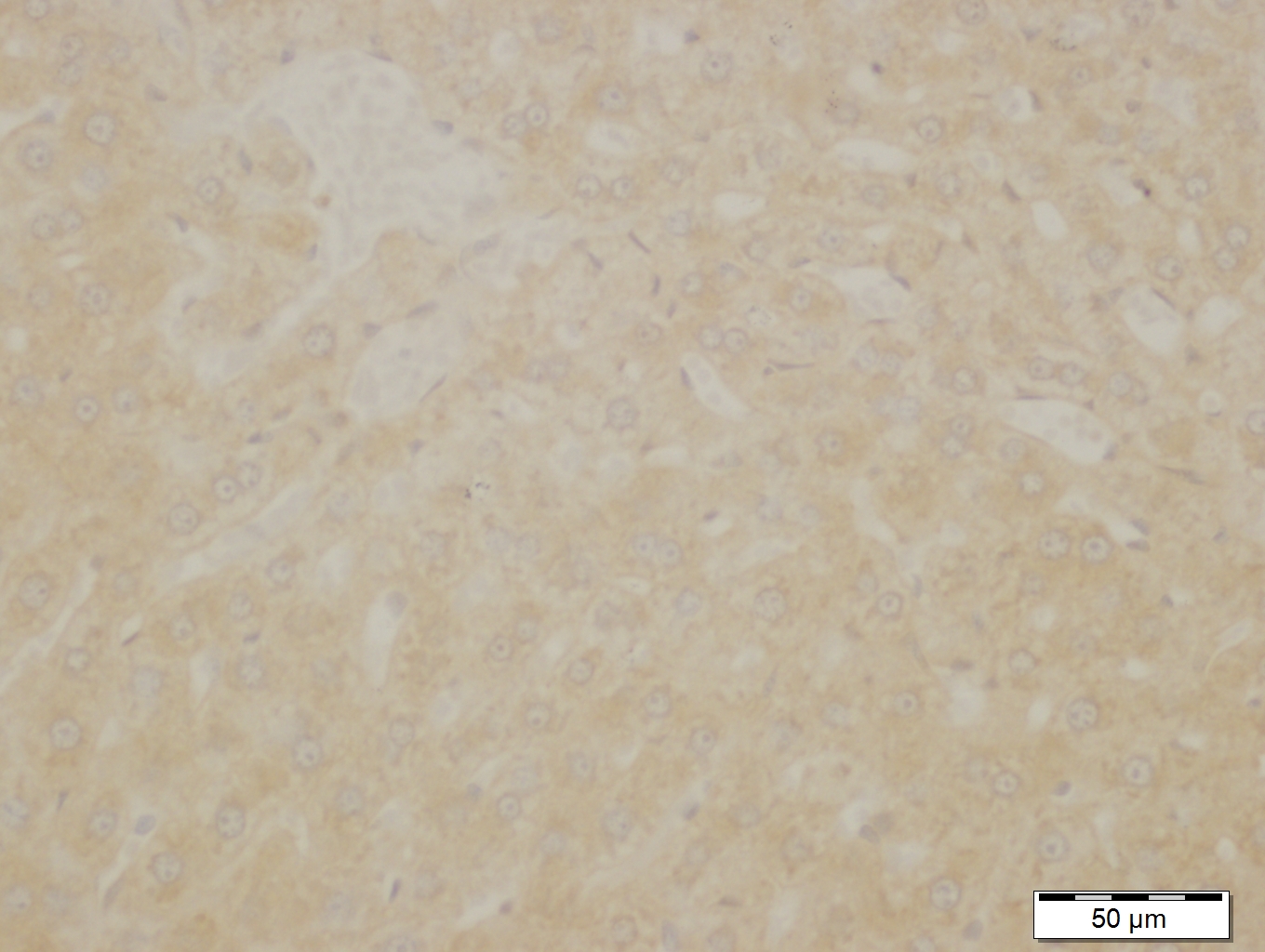

Supplement: Supplementary file 1 [file pharmaceuticals-18-00828-s001.zip › H&E and Immune images/Liver-TNF-KB-Sumayya/Liver-MTX+IAA-TNFKB-X400-8 .jpg]

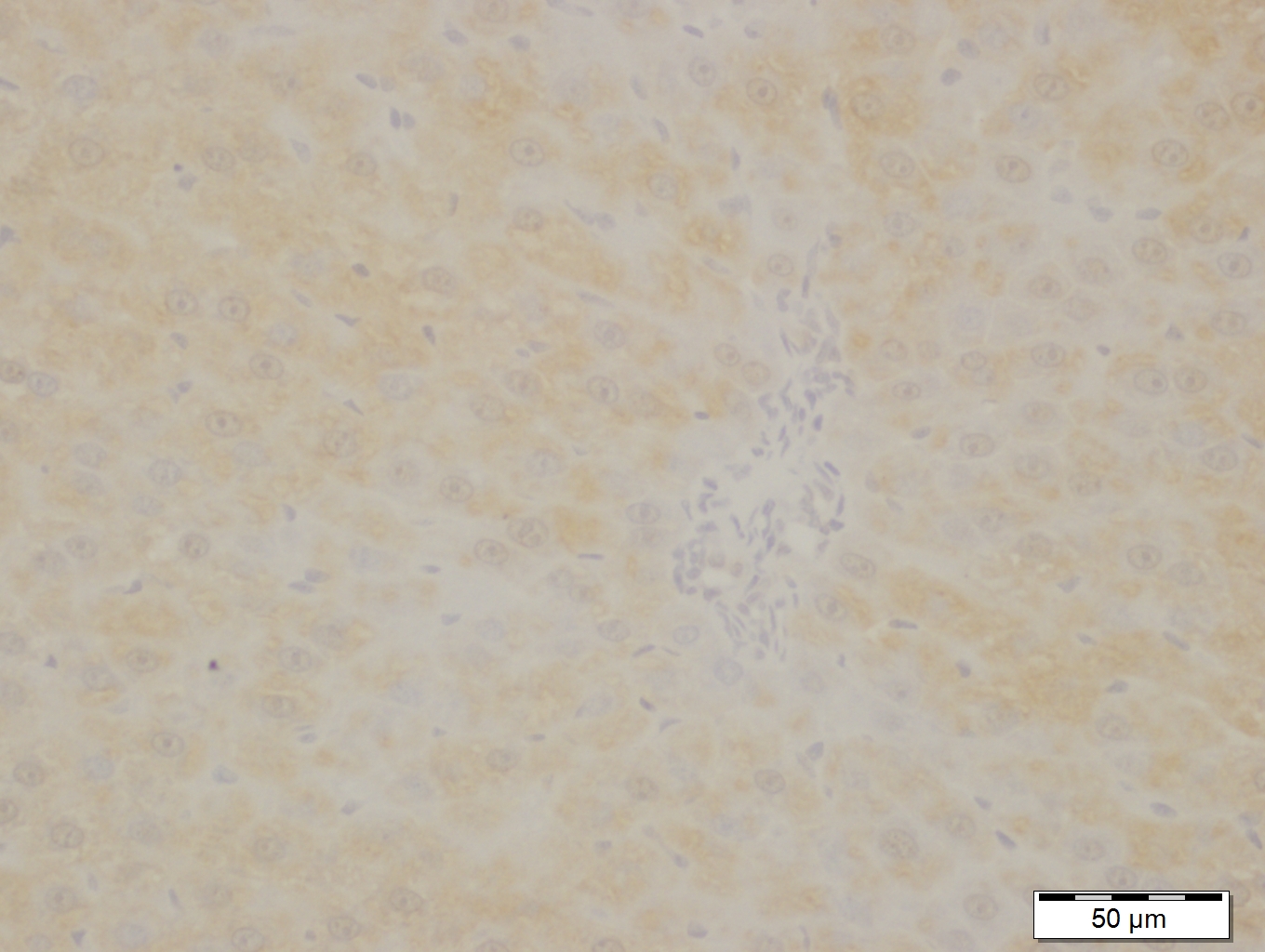

Supplement: Supplementary file 1 [file pharmaceuticals-18-00828-s001.zip › H&E and Immune images/Liver-TNF-KB-Sumayya/Liver-MTX+IAA-TNFKB-X400-9 .jpg]

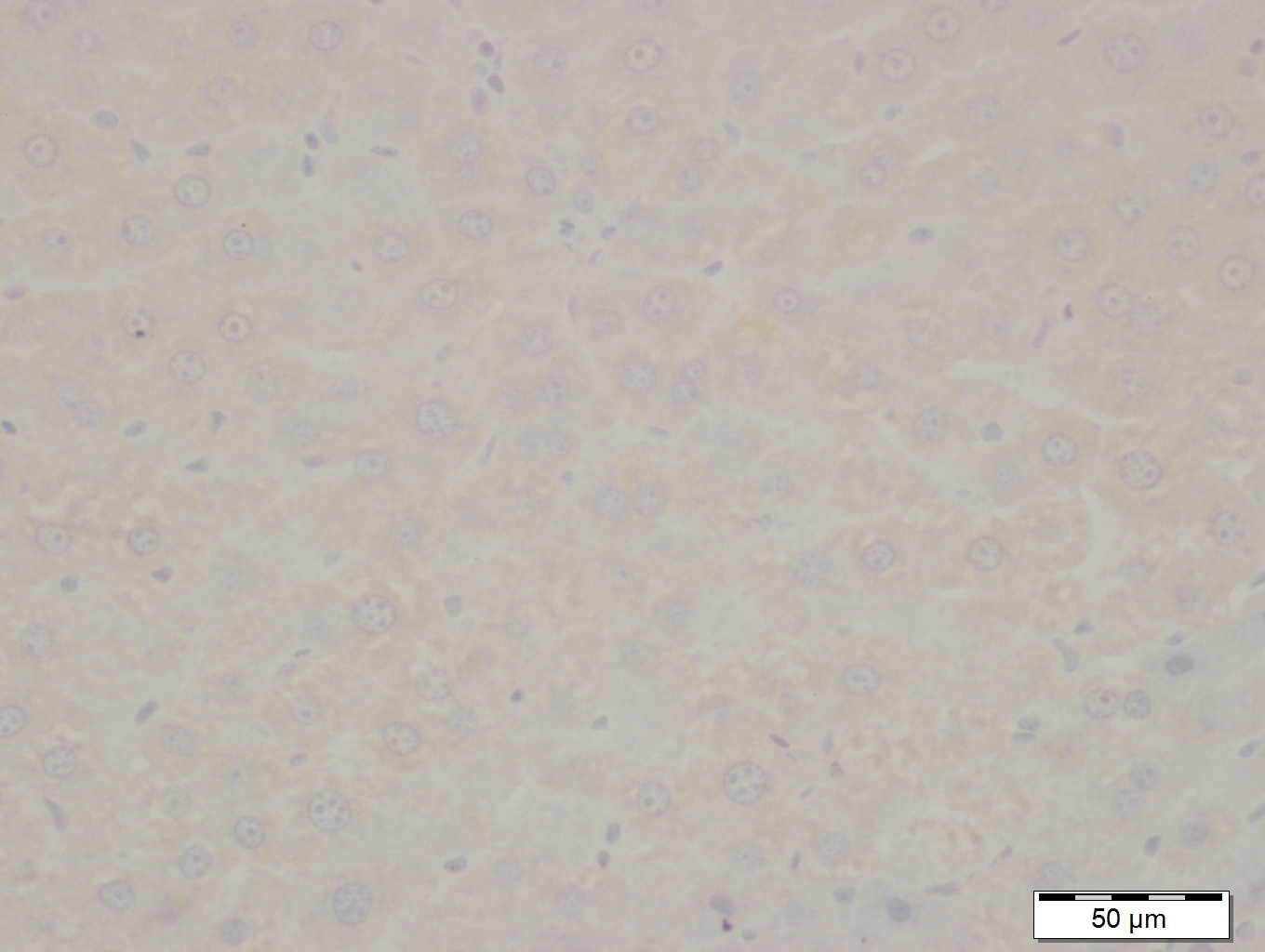

Supplement: Supplementary file 1 [file pharmaceuticals-18-00828-s001.zip › H&E and Immune images/Liver-TNF-KB-Sumayya/Liver-MTX+QUR-TNFKB-X400-1 .jpg]

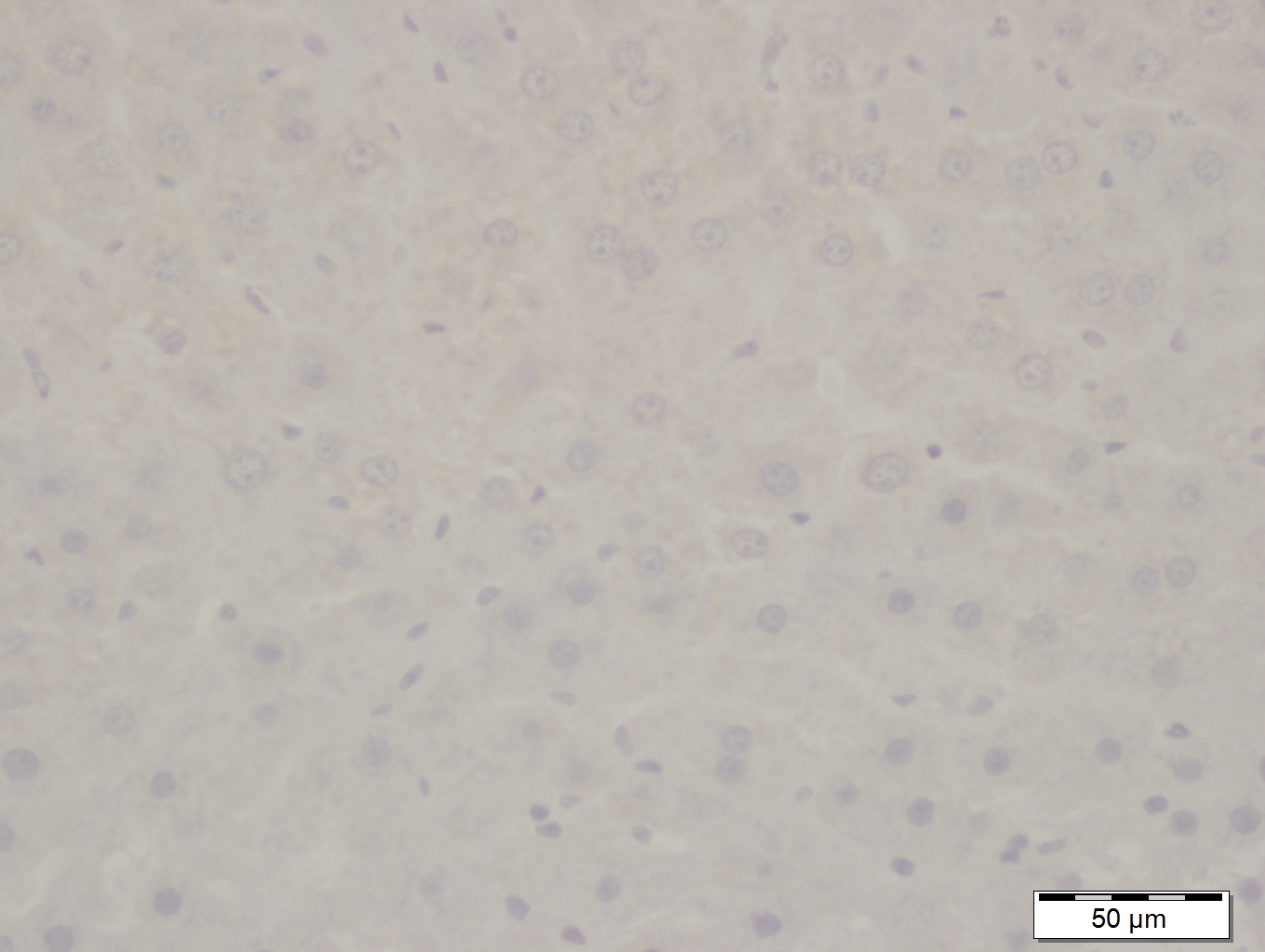

Supplement: Supplementary file 1 [file pharmaceuticals-18-00828-s001.zip › H&E and Immune images/Liver-TNF-KB-Sumayya/Liver-MTX+QUR-TNFKB-X400-3 .jpg]

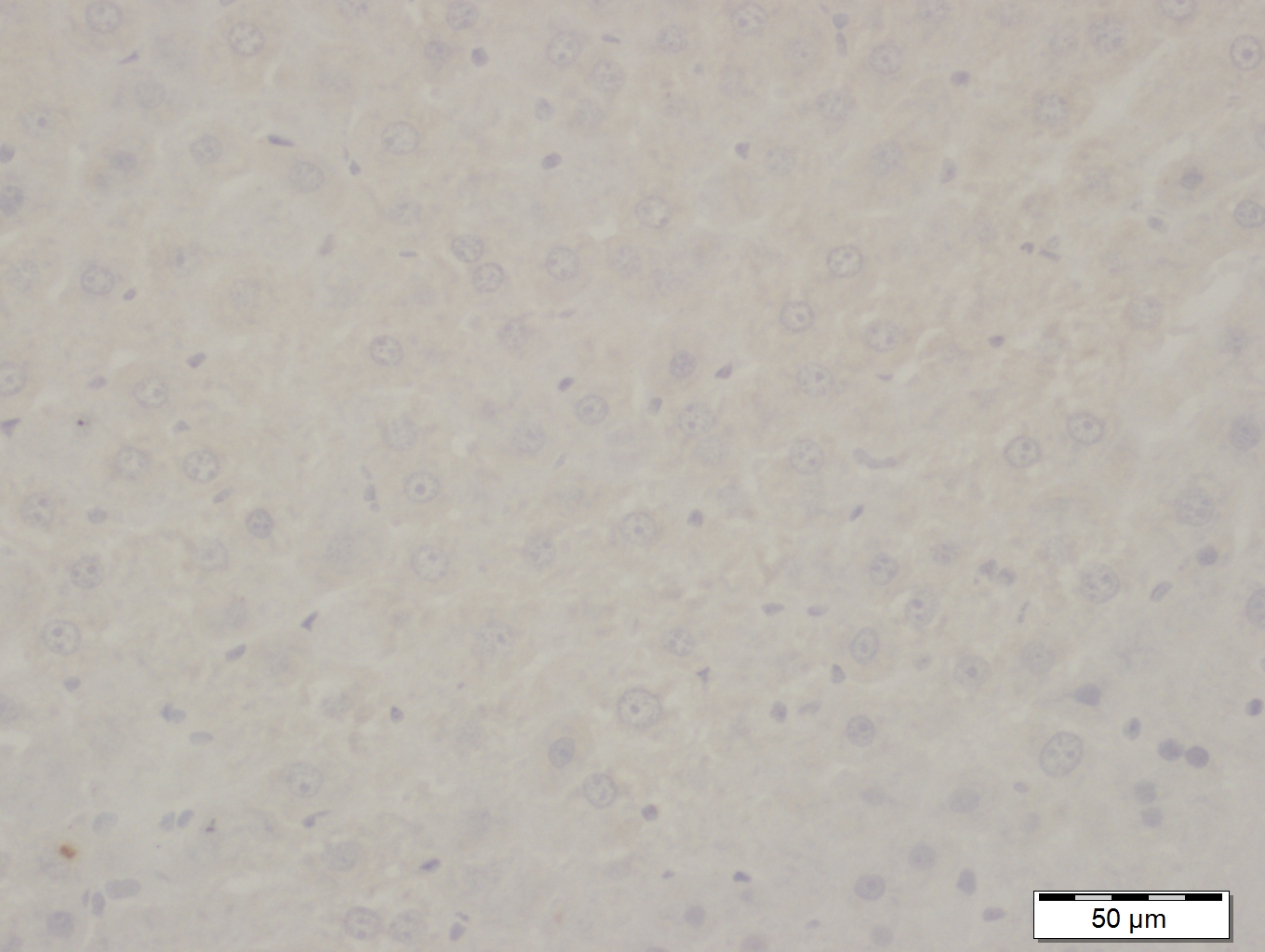

Supplement: Supplementary file 1 [file pharmaceuticals-18-00828-s001.zip › H&E and Immune images/Liver-TNF-KB-Sumayya/Liver-MTX+QUR-TNFKB-X400-4 .jpg]

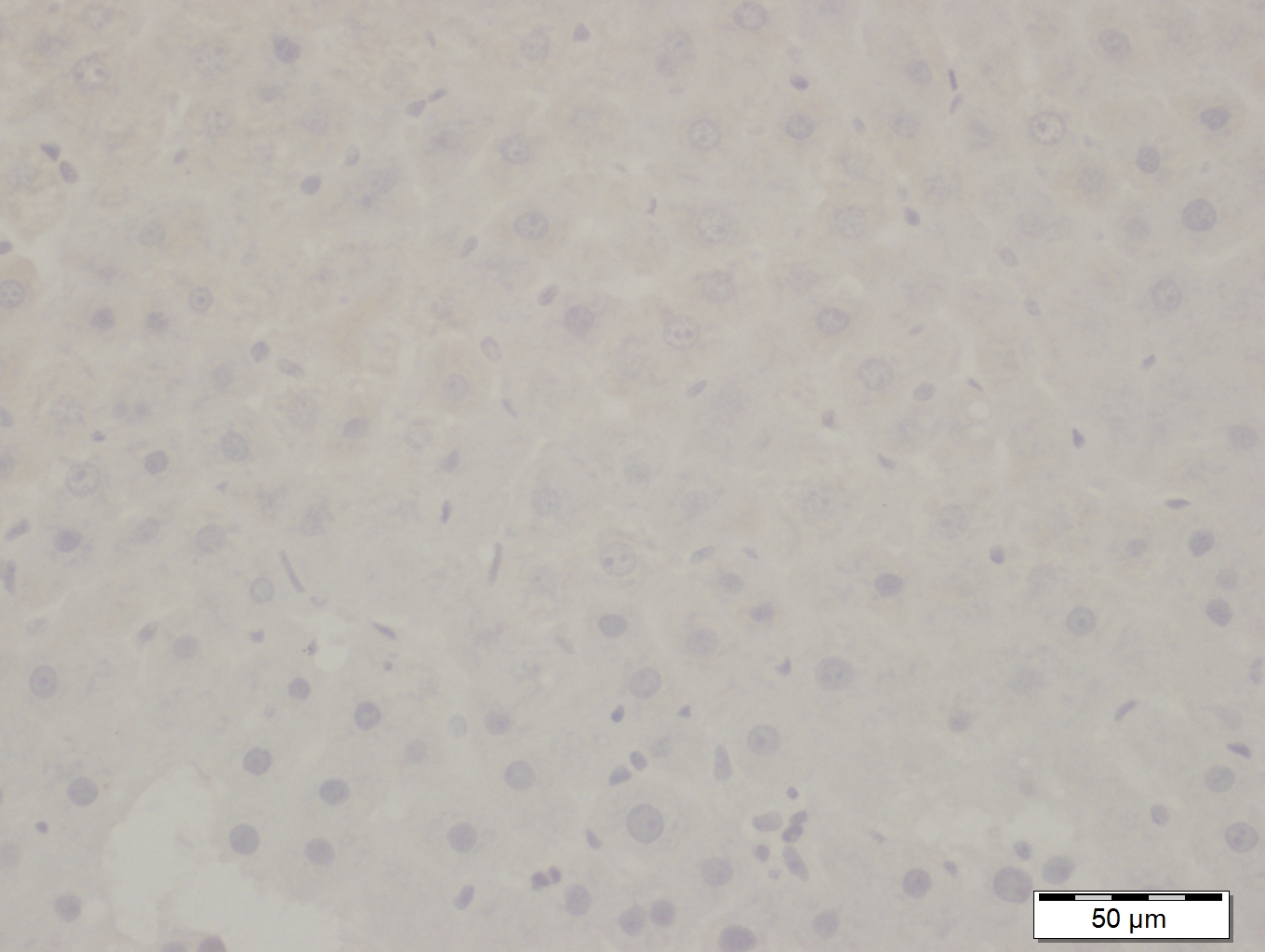

Supplement: Supplementary file 1 [file pharmaceuticals-18-00828-s001.zip › H&E and Immune images/Liver-TNF-KB-Sumayya/Liver-MTX+QUR-TNFKB-X400-5 .jpg]

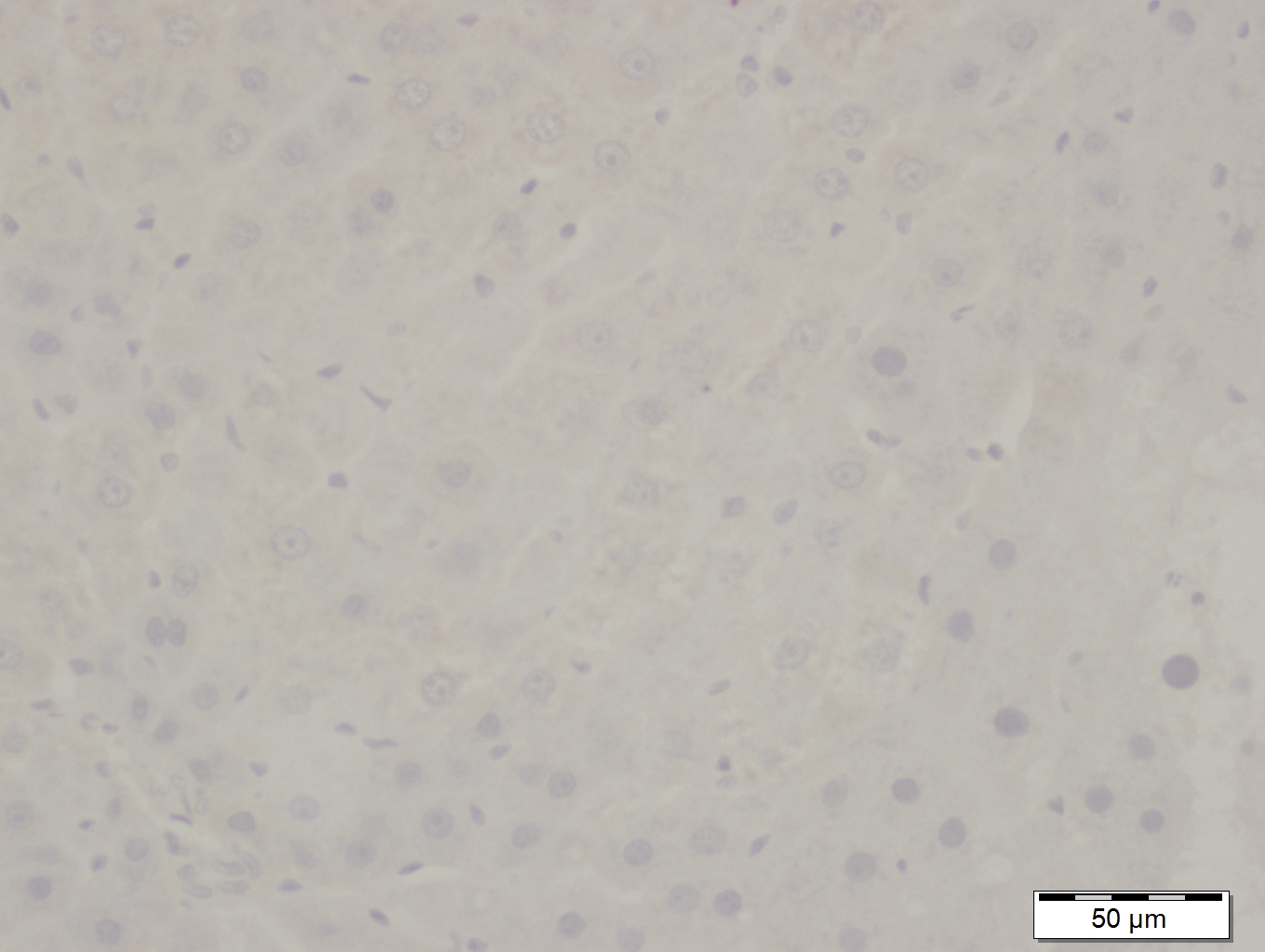

Supplement: Supplementary file 1 [file pharmaceuticals-18-00828-s001.zip › H&E and Immune images/Liver-TNF-KB-Sumayya/Liver-MTX+QUR-TNFKB-X400-6 .jpg]

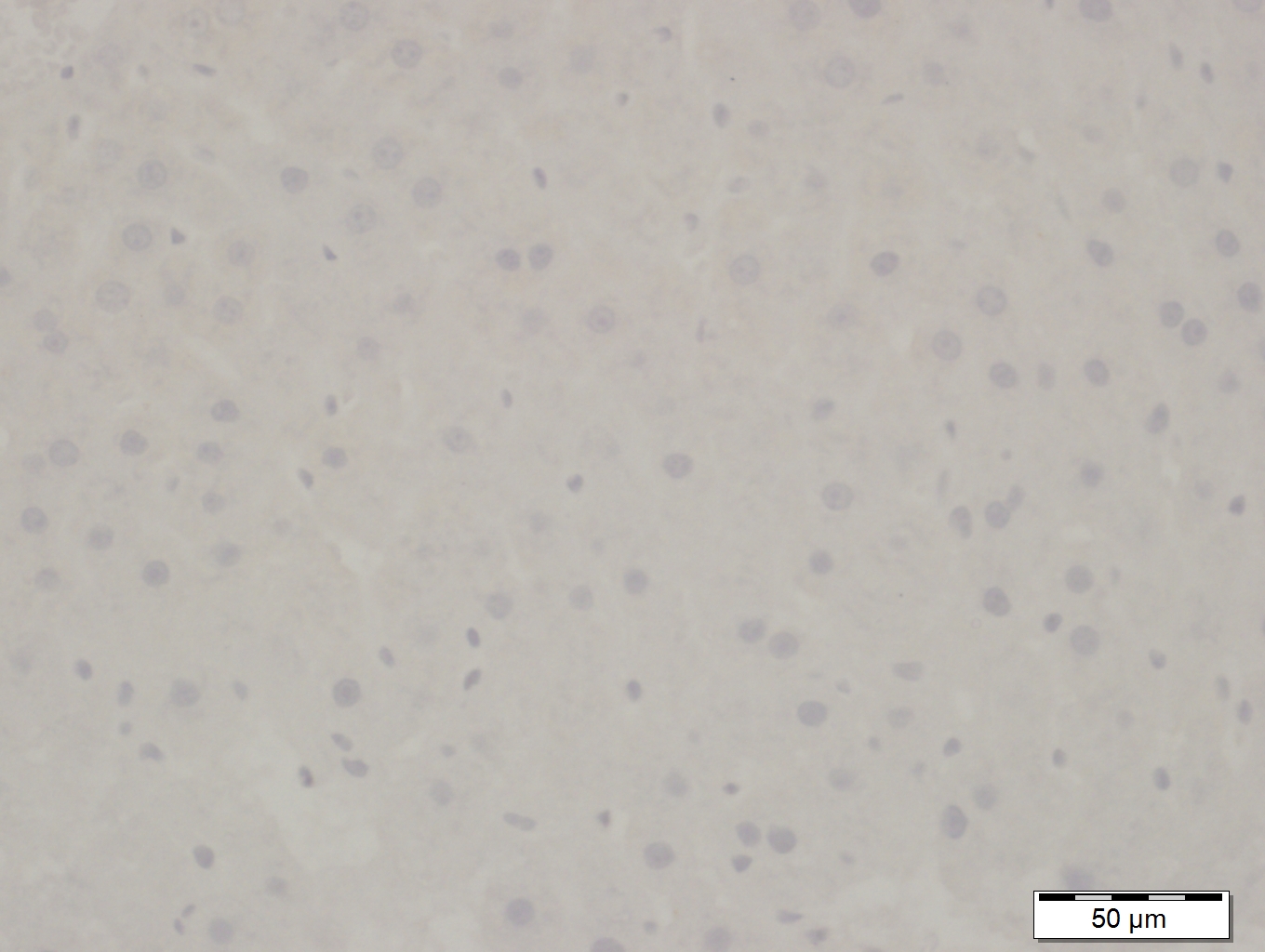

Supplement: Supplementary file 1 [file pharmaceuticals-18-00828-s001.zip › H&E and Immune images/Liver-TNF-KB-Sumayya/Liver-MTX+QUR-TNFKB-X400-7 .jpg]

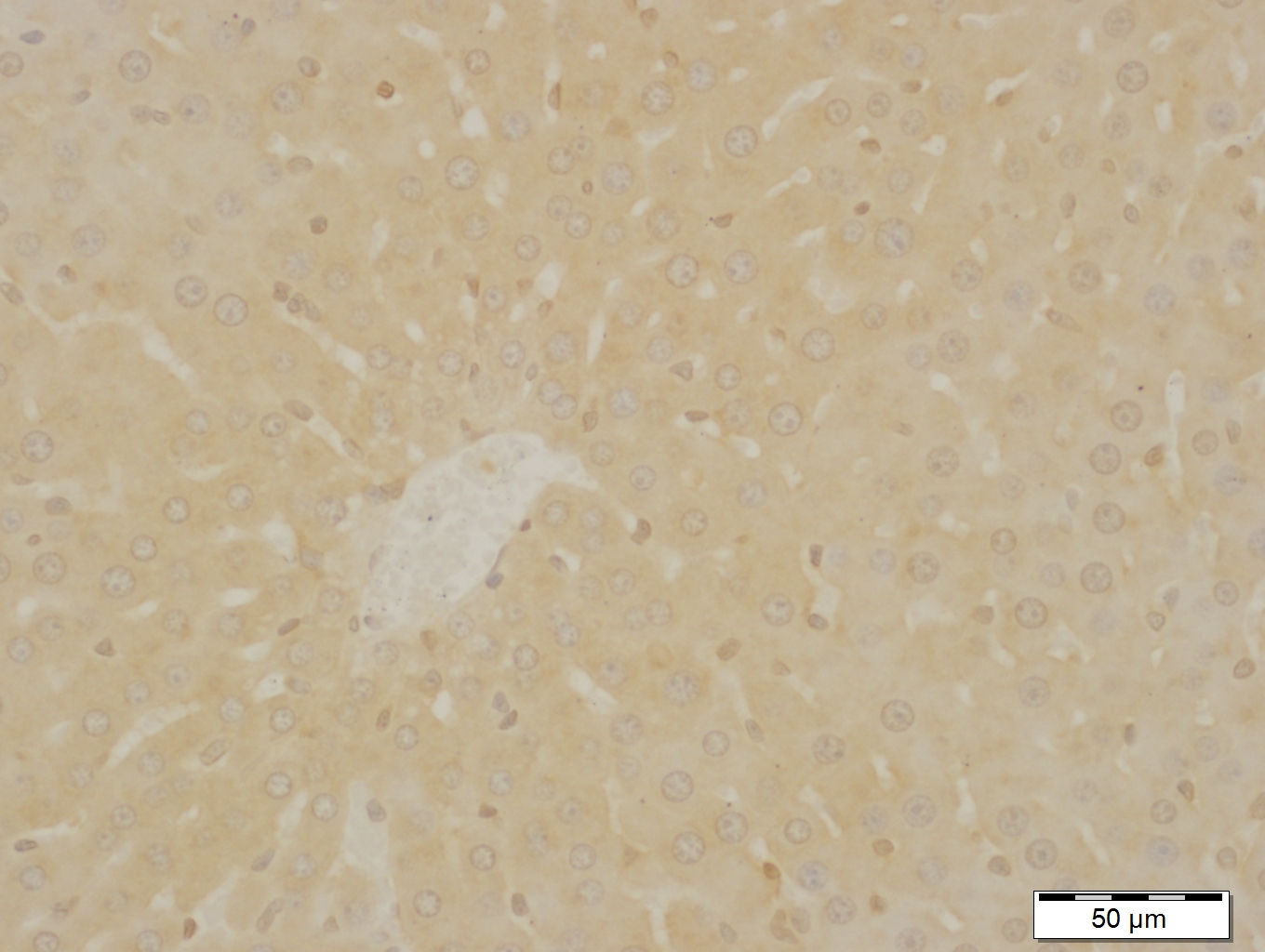

Supplement: Supplementary file 1 [file pharmaceuticals-18-00828-s001.zip › H&E and Immune images/Liver-TNF-KB-Sumayya/Liver-MTX-TNFKB-X400-1 .jpg]

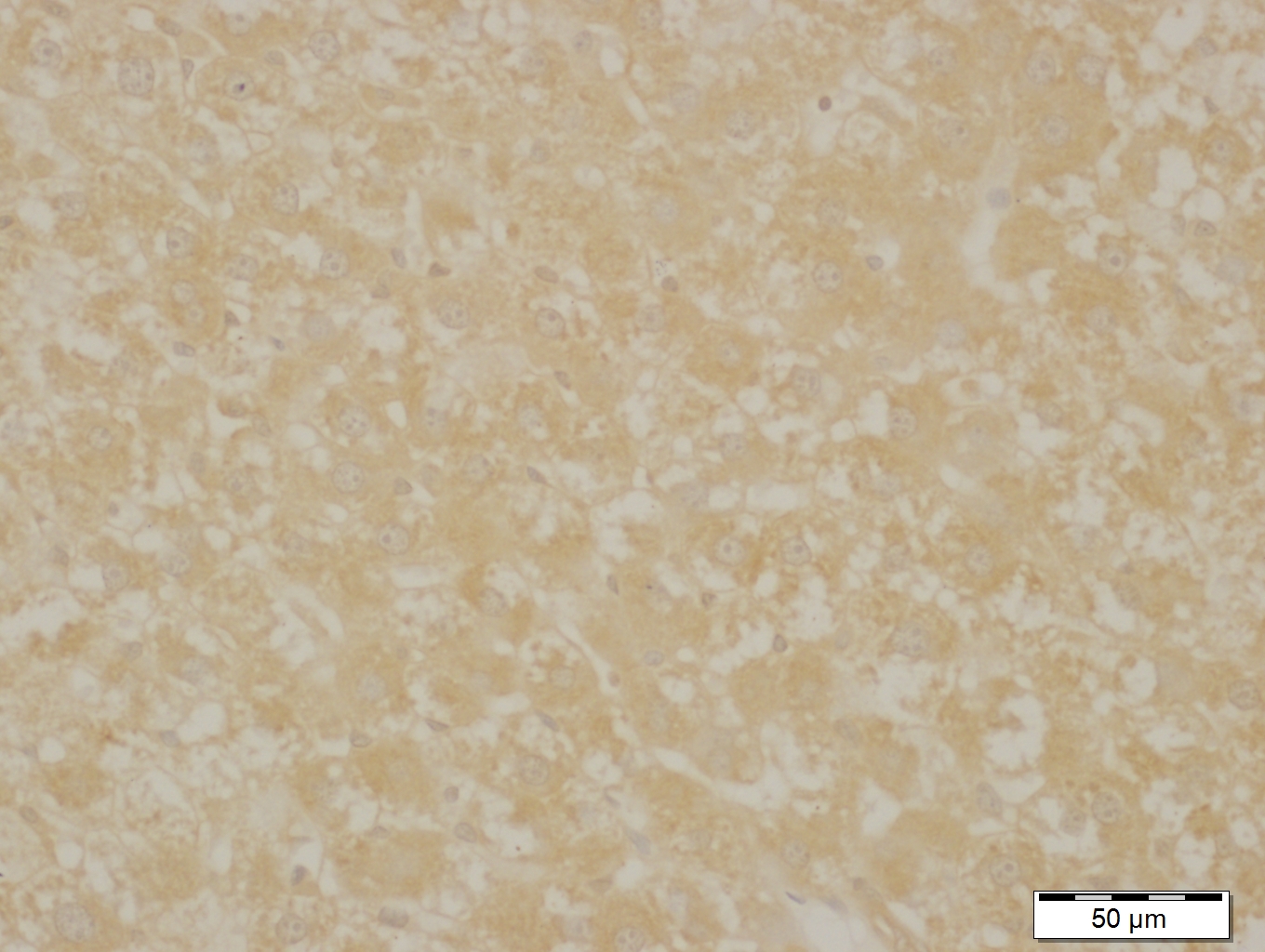

Supplement: Supplementary file 1 [file pharmaceuticals-18-00828-s001.zip › H&E and Immune images/Liver-TNF-KB-Sumayya/Liver-MTX-TNFKB-X400-11 .jpg]

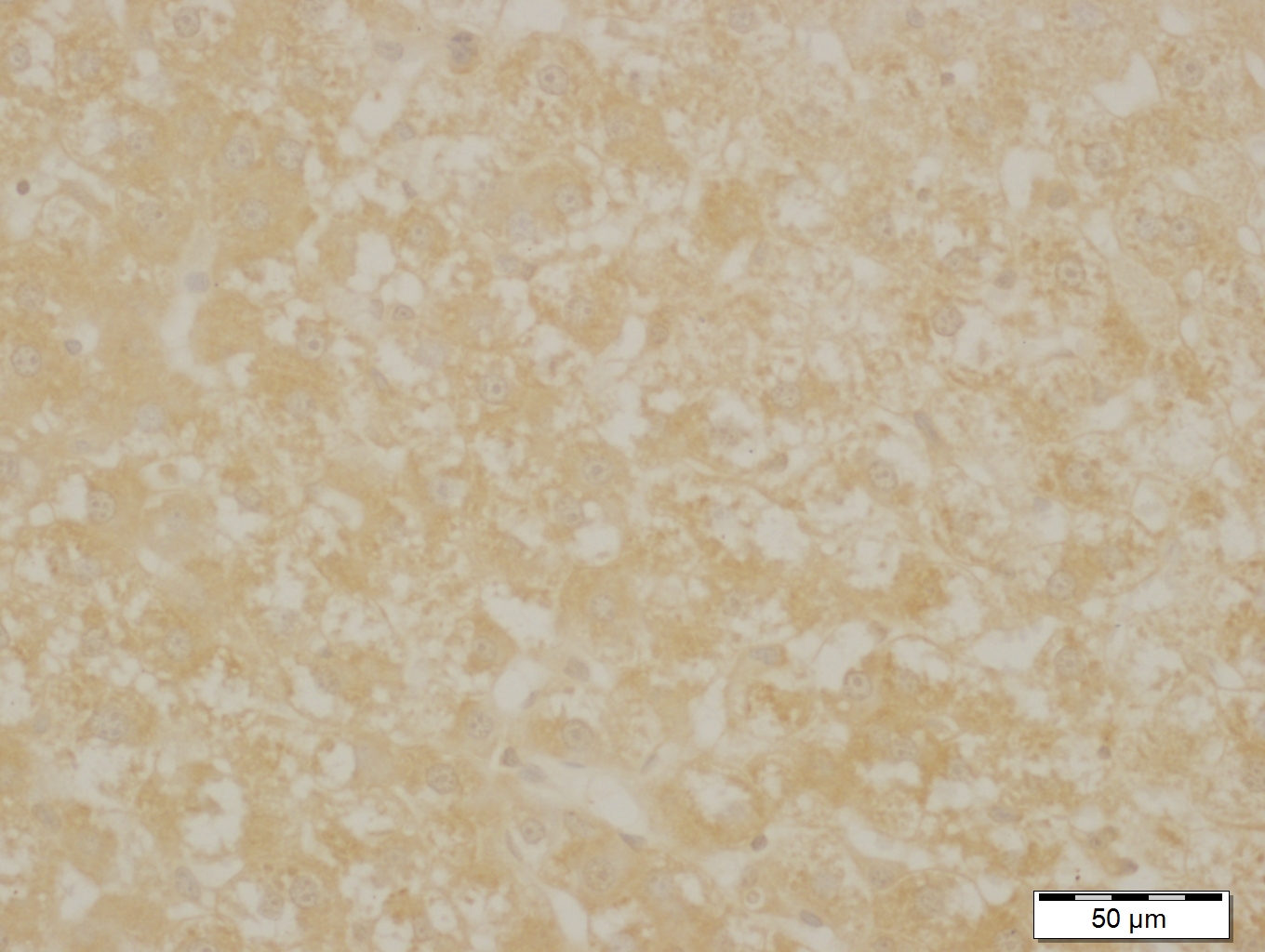

Supplement: Supplementary file 1 [file pharmaceuticals-18-00828-s001.zip › H&E and Immune images/Liver-TNF-KB-Sumayya/Liver-MTX-TNFKB-X400-12 .jpg]

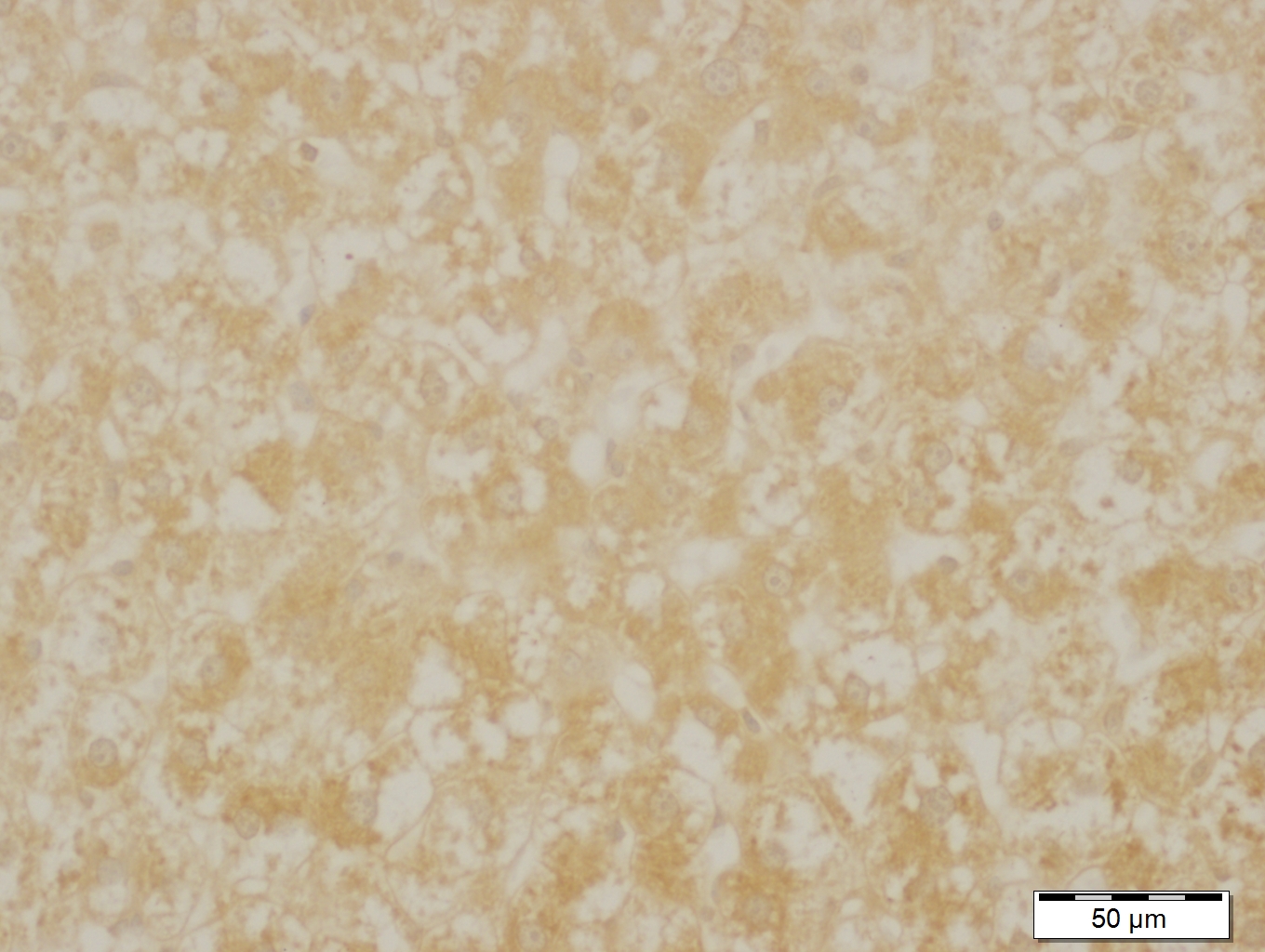

Supplement: Supplementary file 1 [file pharmaceuticals-18-00828-s001.zip › H&E and Immune images/Liver-TNF-KB-Sumayya/Liver-MTX-TNFKB-X400-13 .jpg]

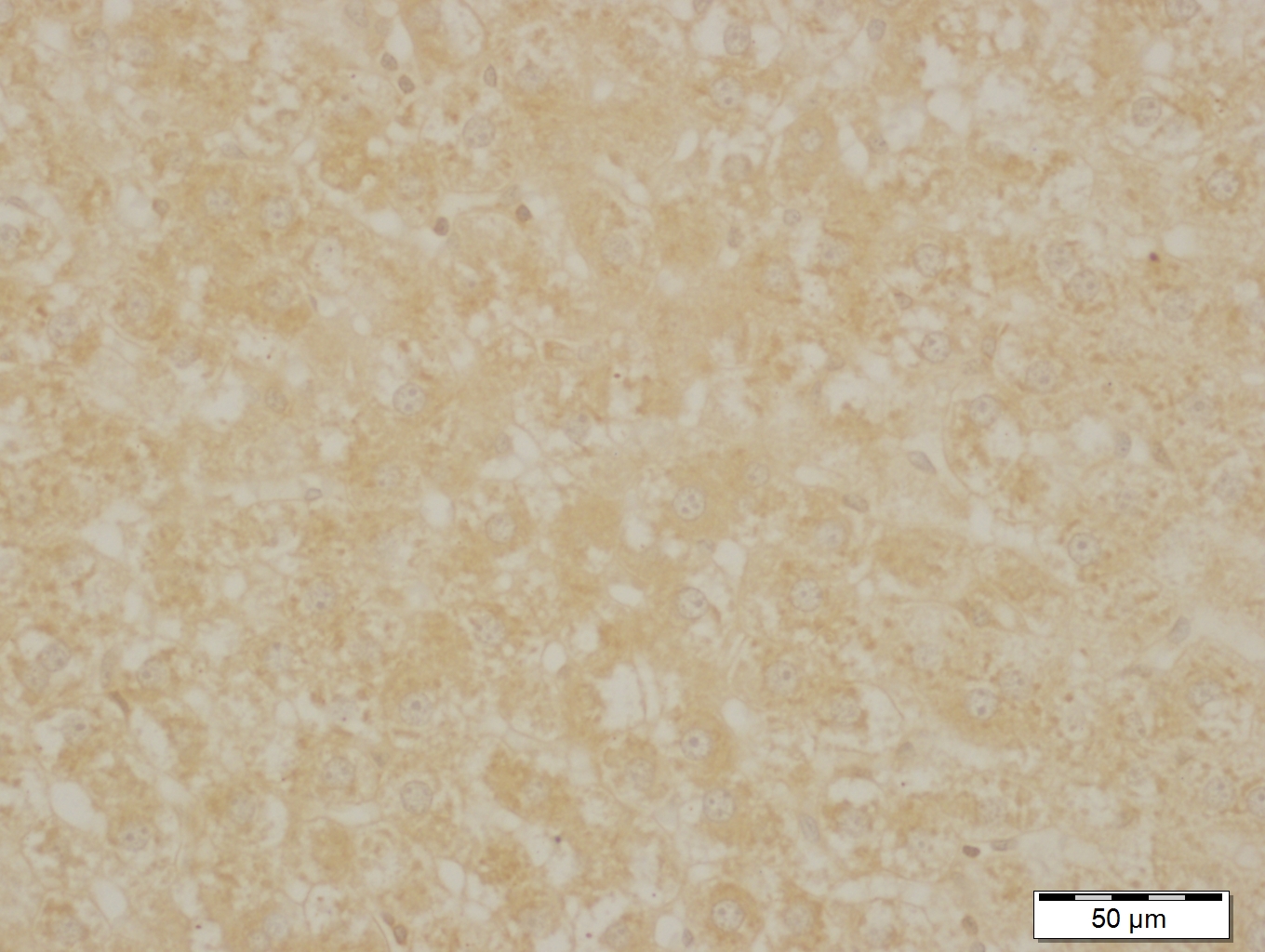

Supplement: Supplementary file 1 [file pharmaceuticals-18-00828-s001.zip › H&E and Immune images/Liver-TNF-KB-Sumayya/Liver-MTX-TNFKB-X400-14 .jpg]

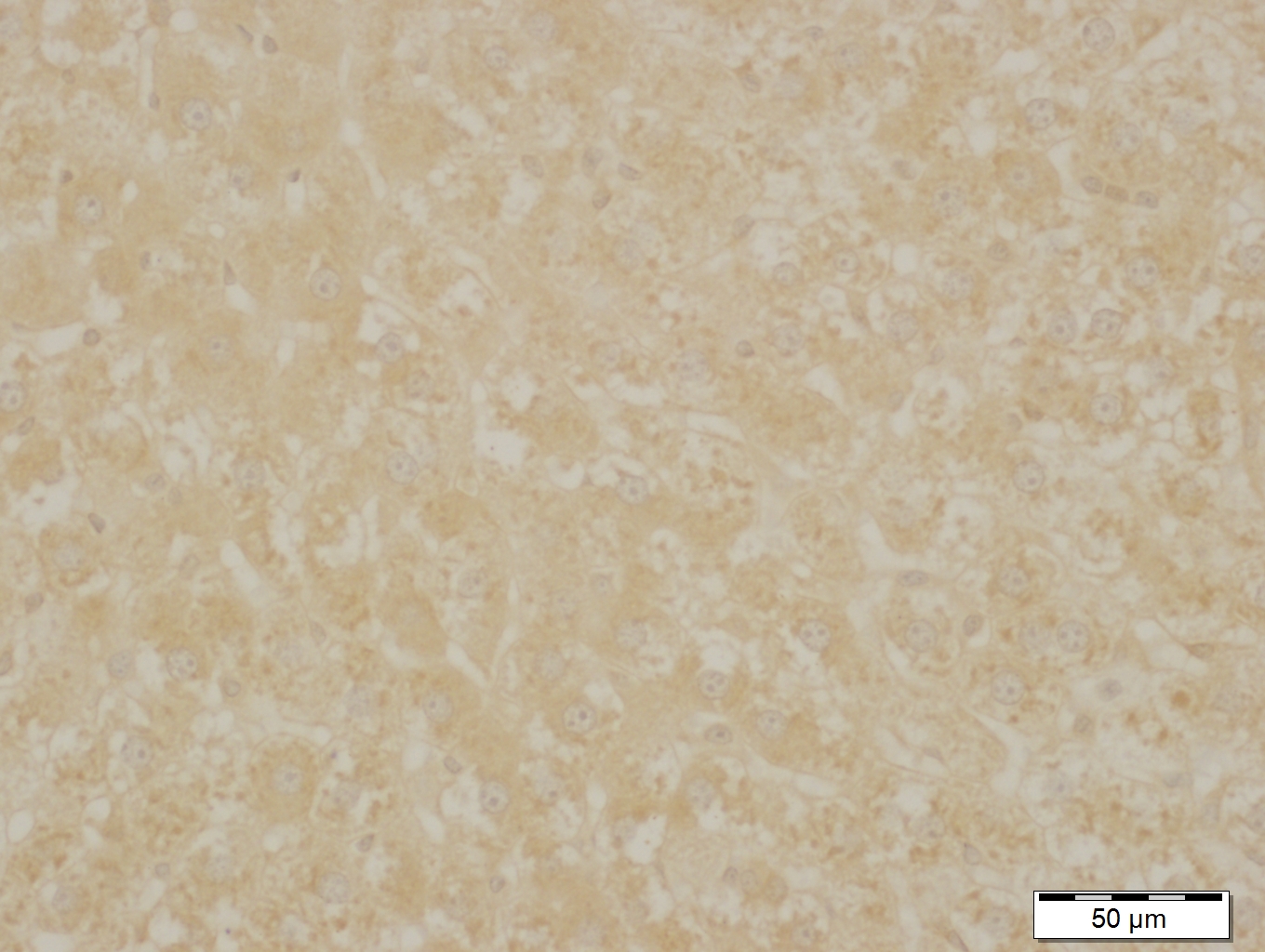

Supplement: Supplementary file 1 [file pharmaceuticals-18-00828-s001.zip › H&E and Immune images/Liver-TNF-KB-Sumayya/Liver-MTX-TNFKB-X400-15 .jpg]

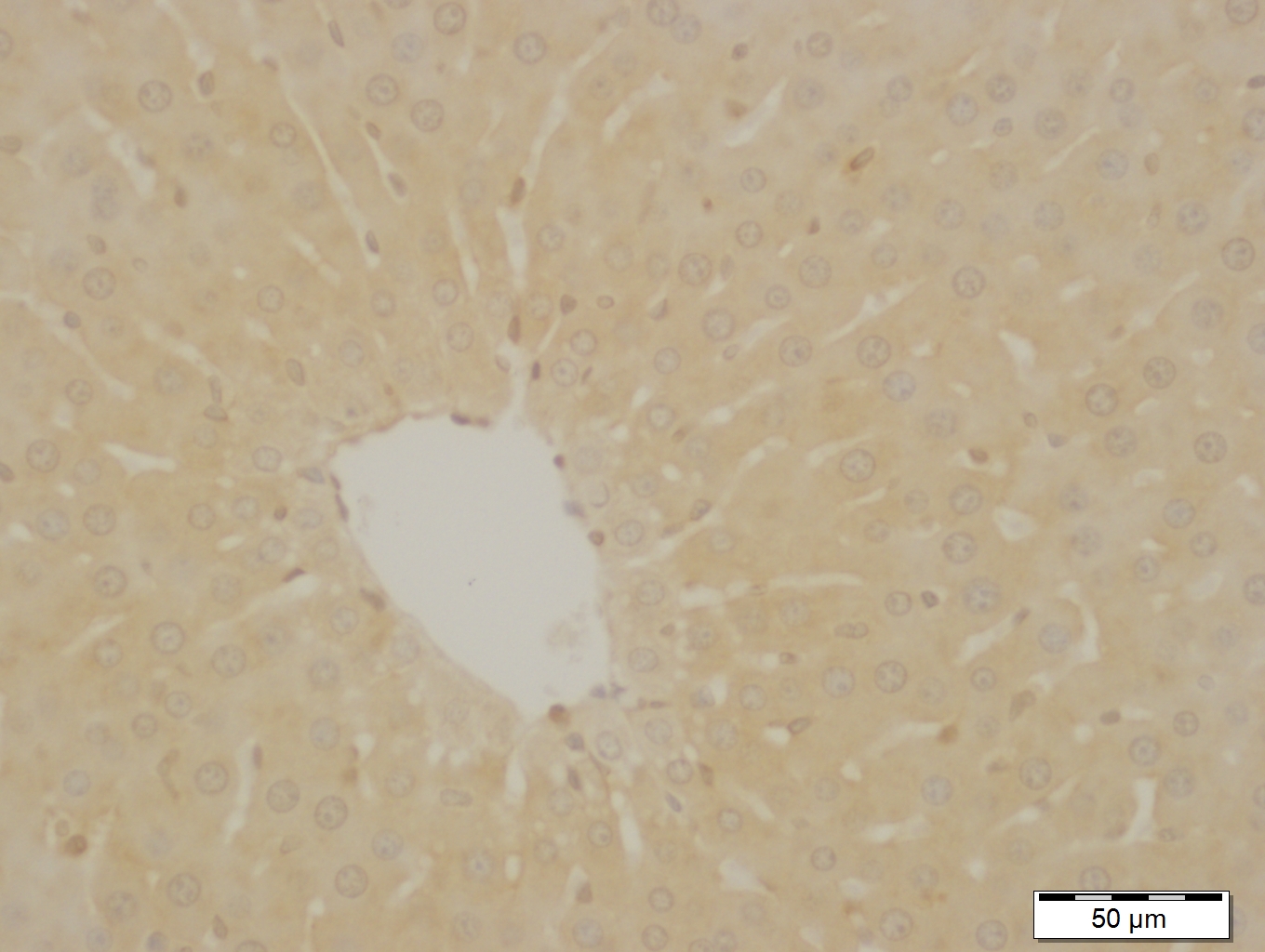

Supplement: Supplementary file 1 [file pharmaceuticals-18-00828-s001.zip › H&E and Immune images/Liver-TNF-KB-Sumayya/Liver-MTX-TNFKB-X400-2 .jpg]

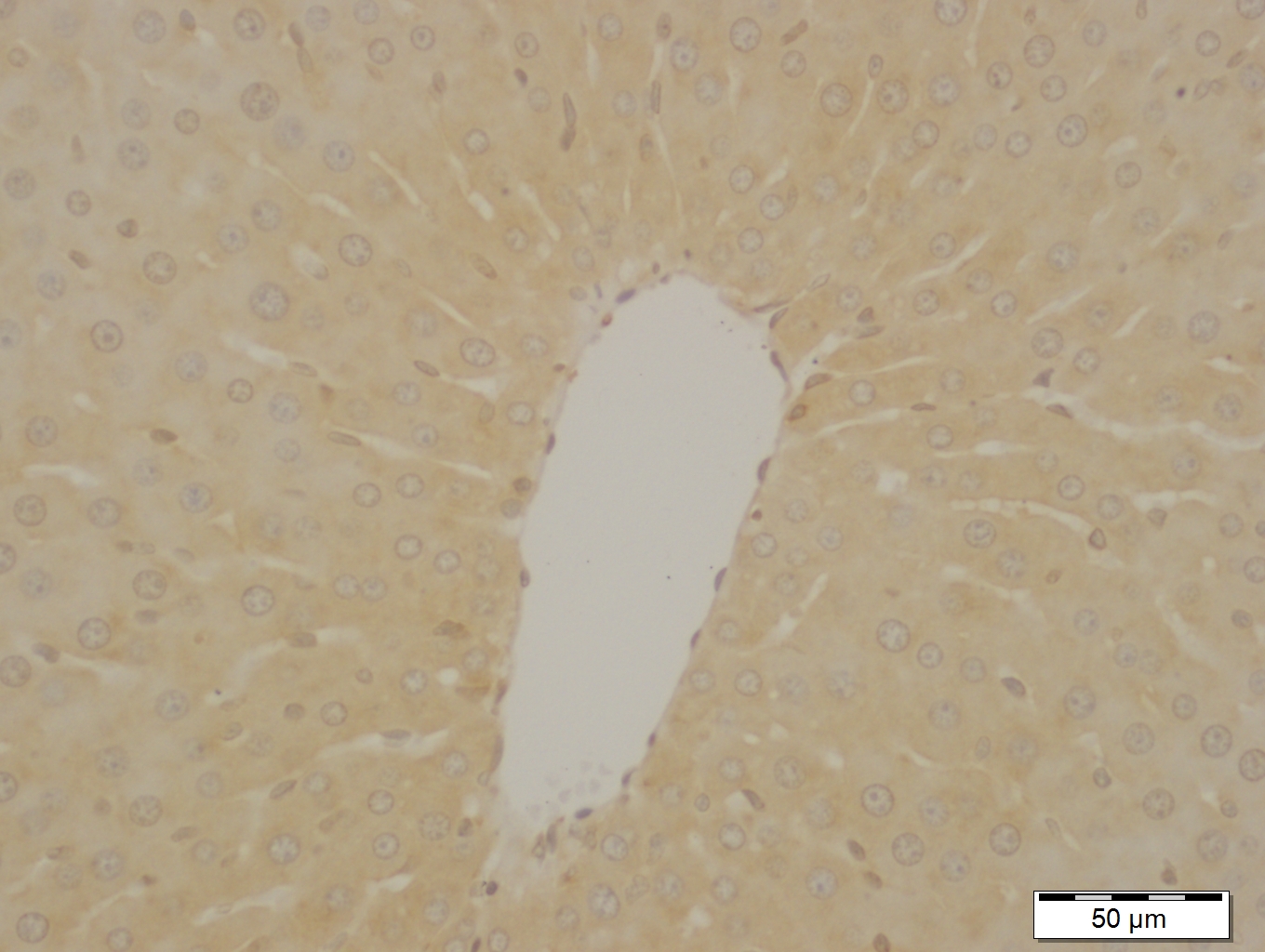

Supplement: Supplementary file 1 [file pharmaceuticals-18-00828-s001.zip › H&E and Immune images/Liver-TNF-KB-Sumayya/Liver-MTX-TNFKB-X400-3 .jpg]

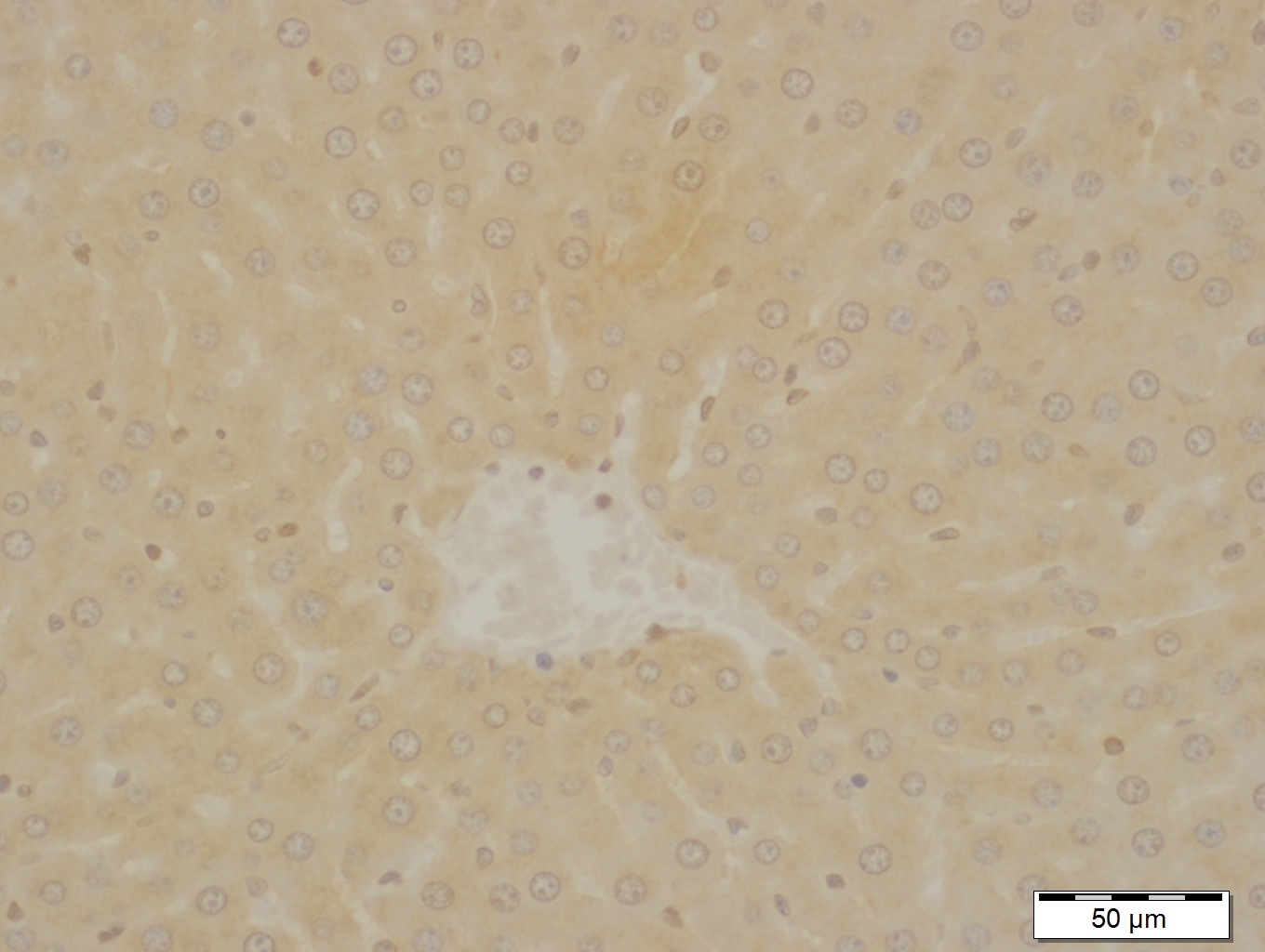

Supplement: Supplementary file 1 [file pharmaceuticals-18-00828-s001.zip › H&E and Immune images/Liver-TNF-KB-Sumayya/Liver-MTX-TNFKB-X400-4 .jpg]

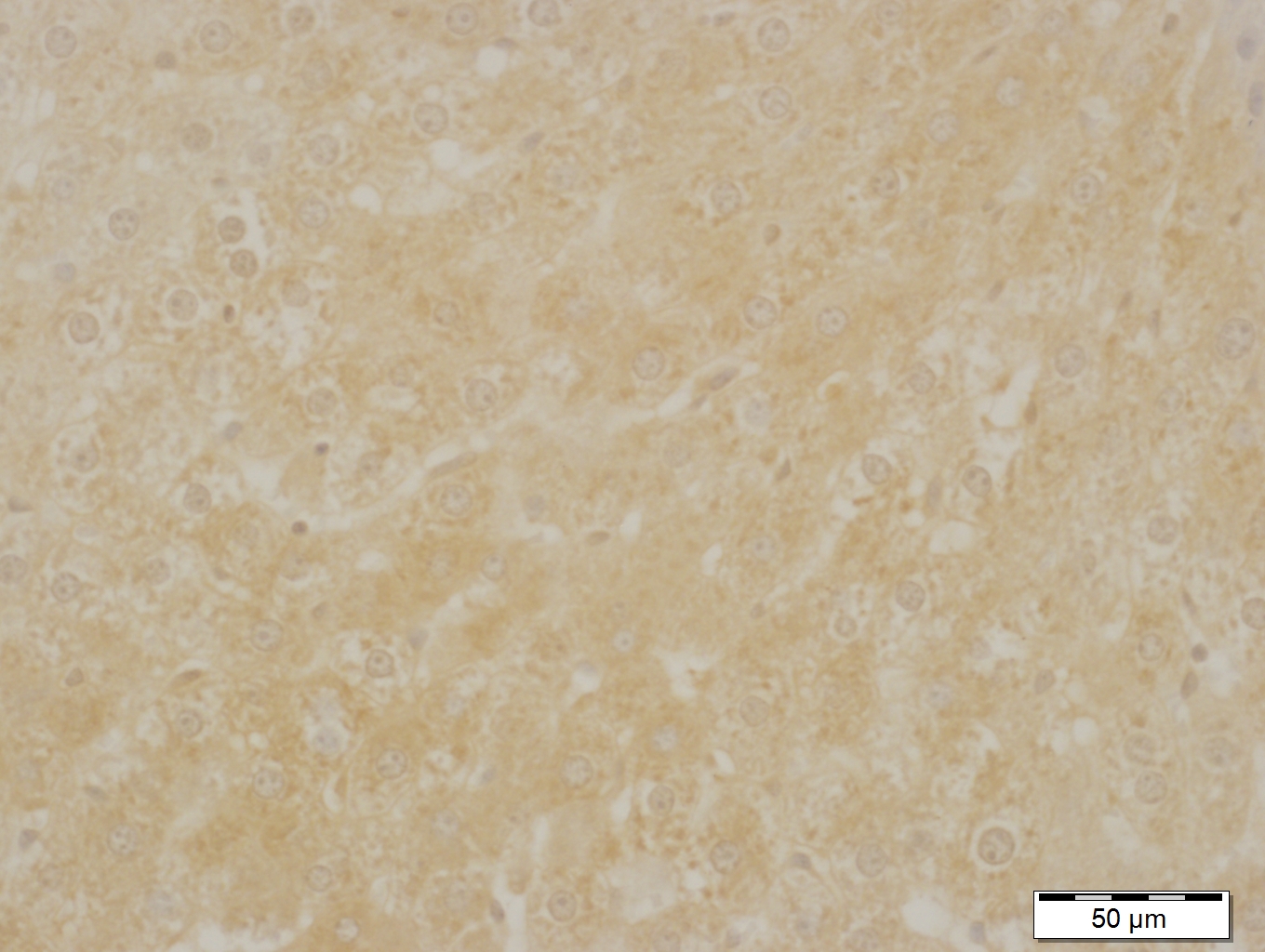

Supplement: Supplementary file 1 [file pharmaceuticals-18-00828-s001.zip › H&E and Immune images/Liver-TNF-KB-Sumayya/Liver-MTX-TNFKB-X400-5 .jpg]

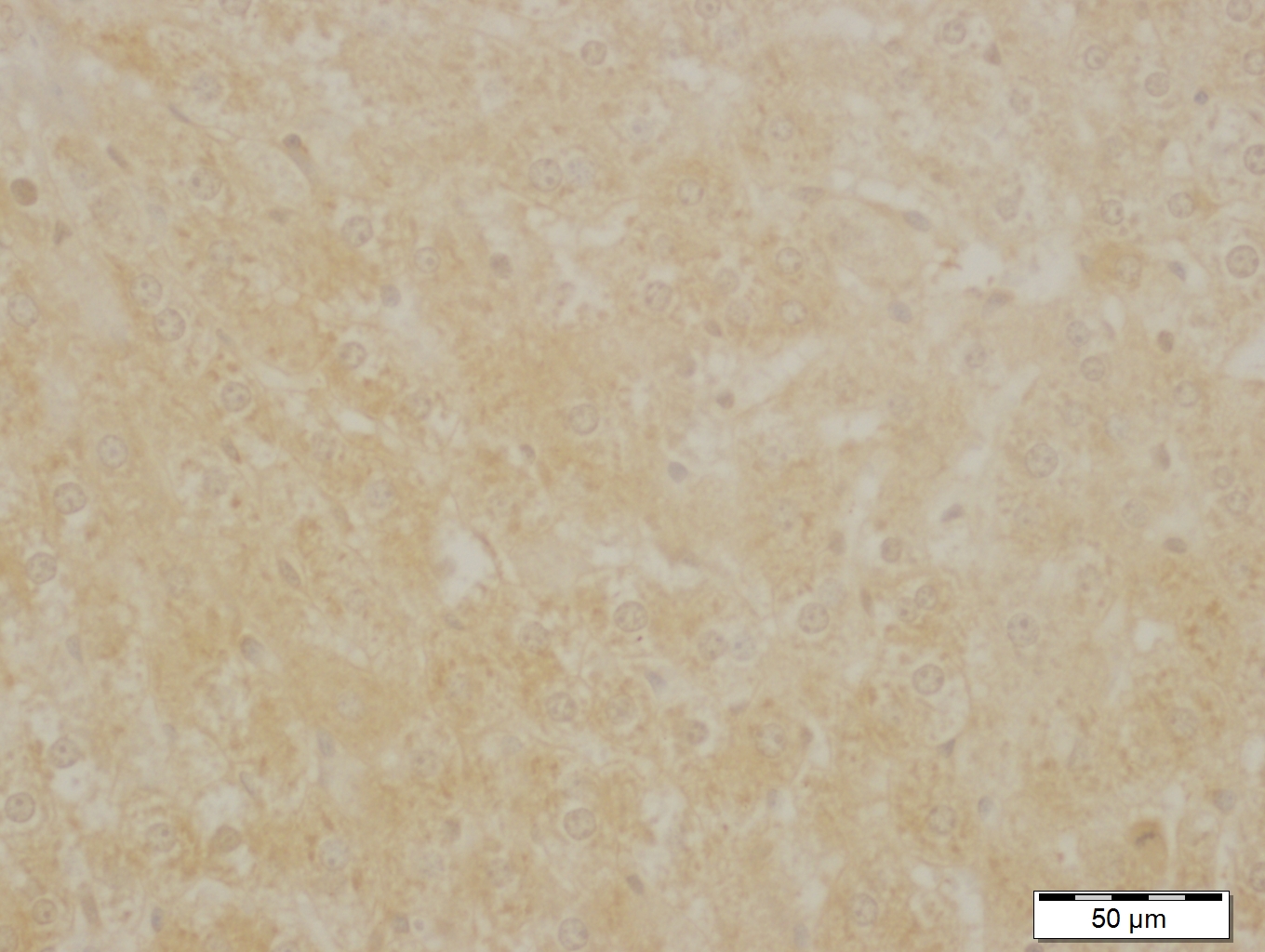

Supplement: Supplementary file 1 [file pharmaceuticals-18-00828-s001.zip › H&E and Immune images/Liver-TNF-KB-Sumayya/Liver-MTX-TNFKB-X400-6 .jpg]

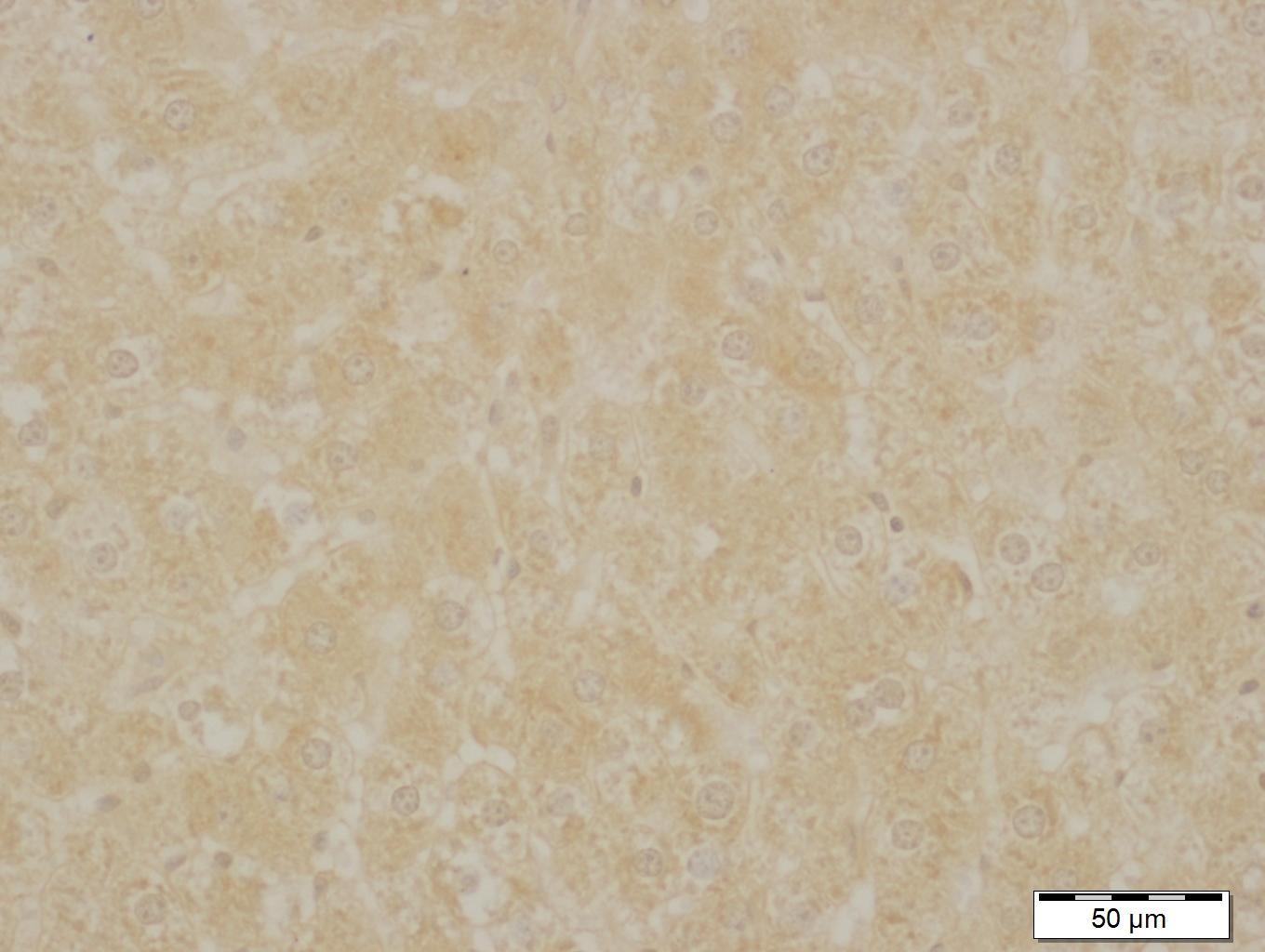

Supplement: Supplementary file 1 [file pharmaceuticals-18-00828-s001.zip › H&E and Immune images/Liver-TNF-KB-Sumayya/Liver-MTX-TNFKB-X400-7 .jpg]

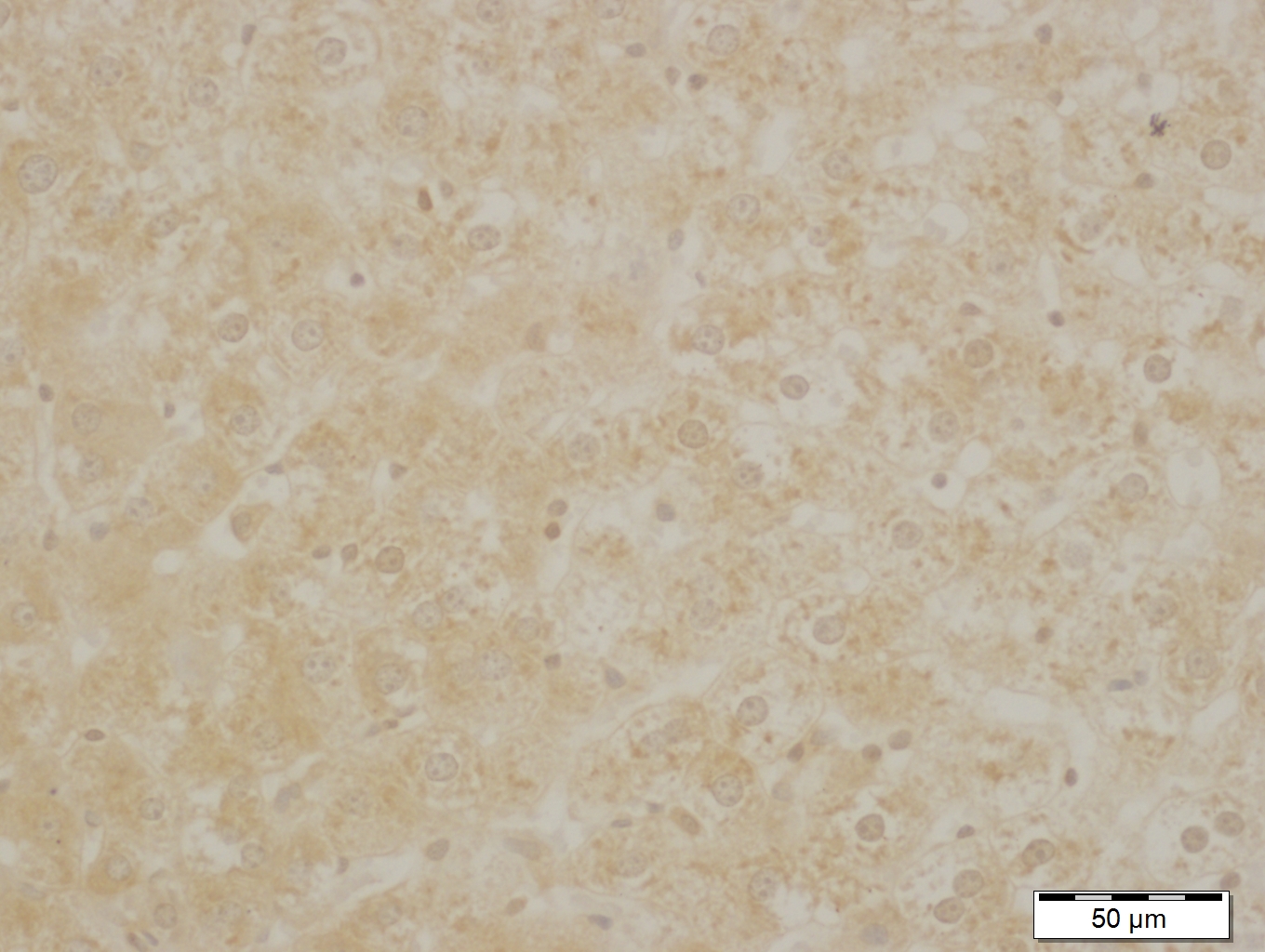

Supplement: Supplementary file 1 [file pharmaceuticals-18-00828-s001.zip › H&E and Immune images/Liver-TNF-KB-Sumayya/Liver-MTX-TNFKB-X400-9 .jpg]
